# Digital music intervention and music therapy across the dementia care continuum: Randomized controlled trial

**DOI:** 10.1002/trc2.70317

**Published:** 2026-08-31

**Authors:** Lotta M. Kaila, Ilja Salakka, Anni Pitkäniemi, Sari Laitinen, Milla Holma, Aleksi J. Sihvonen, Teppo Särkämö

**Affiliations:** ^1^ Centre of Excellence in Music, Mind, Body and Brain Research University of Helsinki & University of Jyväskylä Helsinki Finland; ^2^ Cognitive Brain Research Unit (CBRU), Department of Psychology Faculty of Medicine University of Helsinki Helsinki Finland; ^3^ Espoo Hospital Espoo Finland; ^4^ Musiikkiterapiaosuuskunta (Music Therapy Cooperative) InstruMental Helsinki Finland; ^5^ Brain Centre Helsinki University Hospital Helsinki Finland

**Keywords:** Alzheimer's disease, autobiographical memory, care home, cognitive outcomes, community dwelling, dementia, dementia care continuum, digital music intervention, emotional outcomes, music therapy, non‐pharmacological intervention, randomized controlled trial

## Abstract

**INTRODUCTION:**

Music interventions show promise in dementia care and digital approaches may increase availability and accessibility. We investigated the efficacy of a digital music intervention (DMI) and conventional music therapy (CMT) compared to standard care (SC) across the dementia care continuum.

**METHODS:**

In an exploratory parallel‐group randomized controlled trial, community‐dwelling (HOME, *n *= 91) and care home–dwelling (CARE, *n *= 75) persons living with dementia (*n *= 166) were randomized to DMI, CMT, or SC. Group‐based music interventions spanned 10 weeks (2 × 60‐minute sessions/week) and included receptive (listening, reminiscing) and expressive (singing, movement) activity. DMI comprised pre‐recorded video sessions followed on screen with a non‐therapist facilitator; CMT was delivered live by professional music therapists. Outcomes were assessed at baseline (T1), 3 months (T2), and 6 months (T3), measuring cognition, mood, quality of life, and caregiver well‐being. Primary outcomes were changes in general cognition (Montreal Cognitive Assessment, Consortium to Establish a Registry for Alzheimer's Disease Chandler total; T1–T2). Primary and sensitivity analyses used linear mixed‐effects models in whole, HOME, and CARE samples.

**RESULTS:**

No significant effects of CMT or DMI were found for primary general cognition outcomes in primary or sensitivity analyses. Across exploratory secondary outcomes, compared to SC, CMT improved naming (T1–T2) and verbal learning (T1–T3) in the whole sample and executive function in HOME (T1–T2). Compared to a decline in SC, prompted autobiographical memory was maintained by DMI and CMT in the whole sample (T1–T2) and by DMI in CARE (T1–T2). Sensitivity analyses largely supported these findings. No significant differences were observed between DMI and CMT.

**DISCUSSION:**

CMT and DMI did not yield effects on primary general cognition outcomes. CMT showed modest but coherent effects on exploratory secondary cognitive outcomes across the dementia care continuum. The only exploratory secondary finding for DMI was maintenance of autobiographical memory against a decline in SC. These findings support existing evidence yet call for further research to optimize interventions.

## Highlights

- This study compared digital and conventional music interventions to standard care in dementia.
- No intervention effects were found on primary general cognitive outcomes.
- Music therapy showed selective cognitive–emotional benefits across the care continuum.
- Autobiographical memory was maintained by both interventions across the care continuum.
- Exploratory secondary results support existing evidence on music in dementia care.

## 1 INTRODUCTION

Dementia, primarily caused by Alzheimer's disease (AD), continues to rise in prevalence with aging populations, increasing burden for health‐care systems, society, and caregivers.^1^ With the first disease‐modifying treatments arriving for dementias, pharmacological interventions remain focused on managing cognitive, behavioral, emotional, and social symptoms, often with adverse effects.^2^ Non‐pharmacological interventions, therefore, may offer less invasive and more person‐centered and cost‐effective approaches to treatment. Notably, current guidelines recommend non‐pharmacological interventions particularly for emotional and psychosocial symptoms in early‐stage dementia.^2^

Engaging widespread reward, cognitive, and emotional neural networks,^3^ music is a compelling approach to non‐pharmacological intervention. As medial prefrontal areas related to musical memory and emotions are relatively well preserved through AD pathology,^4^, ^5^ music can support autobiographical memory (AM) in dementia, crucial for the quality of life (QOL) of persons living with dementia.^6^, ^7^ Music‐based interventions have also shown to enhance cognitive (general cognition, episodic memory, executive function), emotional (anxiety, agitation, depression), and psychosocial (QOL, caregiver burden) functioning in dementia.^8^, ^9^, ^10^, ^11^, ^12^ While music interventions show promise in dementia care, current evidence remains moderate to low in quality.^12^ Many music‐based randomized controlled trials (RCTs) are implemented in residential care for persons living with either mild/moderate or moderate/severe dementia, thereby addressing only specific segments of the dementia care continuum. Like other non‐pharmacological approaches, live music interventions rely on scarce trained health‐care professionals^2^ and may be inaccessible or inapplicable for persons with limited mobility or during exceptional circumstances (e.g., COVID‐19 pandemic).

**RESEARCH IN CONTEXT**

**Systematic Review**: Literature on music‐based interventions in dementia and emerging digital adaptations was reviewed using PubMed and related databases. Current evidence, moderate/low in quality, demonstrates cognitive, emotional, and psychosocial benefits of music interventions. Studies typically focus on specific care settings and lack comparison between digital interventions and conventional therapist‐led interventions.

**Interpretation**: Digital music intervention (DMI) and conventional music therapy (CMT) did not yield effects on primary general cognition outcomes. Concerning secondary outcomes, both CMT and DMI maintained autobiographical memory against a decline in standard care, with additional CMT‐related benefits on naming, verbal learning, executive function, and anxiety. CMT seems to have a greater number of benefits in dementia, while DMI primarily offers availability and accessibility.

**Future Directions**: Further research is needed to optimize music interventions in dementia. Research is also warranted to investigate music‐evoked autobiographical memory as a potential mechanism for well‐being in dementia, and the relationship between demographic, clinical, and musical background and intervention efficacy.

Digital adaptations could improve the availability and accessibility of music interventions by reducing dependence on restricting factors such as time or location. Recent digital intervention studies using a music component have yielded promising results on cognitive, motor, and emotional benefits for persons living with dementia.^13^, ^14^, ^15^, ^16^ These interventions, however, were implemented by trained professionals using an online videoconferencing platform and included only few typical components of music therapy in dementia care (music listening, singing, playing/musical movement). Most studies did not compare the digital intervention to a conventional intervention or explore effects across the dementia care continuum.

This RCT investigated the efficacy of a group‐based digital music intervention (DMI) and group‐based conventional music therapy (CMT) across the dementia care continuum, compared to standard care (SC). The DMI comprised pre‐recorded music‐based sessions followed on screen with a non‐therapist facilitator encouraging participation. We aimed to explore the effects of DMI and CMT on cognitive, emotional, and psychosocial functioning of community‐ and care home–dwelling persons living with dementia. Given that music interventions delivered in person and digitally can have beneficial effects in dementia care,^8^, ^9^, ^10^, ^11^, ^12^, ^13^, ^14^, ^15^, ^16^ we expected that both CMT and DMI would show benefits on general cognition primarily and secondarily on cognitive, emotional, and psychosocial outcomes and well‐being of caregivers, compared to SC. In addition, given that non‐pharmacological interventions may be most effective for early‐stage symptoms,^2^ we hypothesized that DMI and CMT would show greater effects for community‐dwelling than care home–dwelling participants.

## 2 METHODS

### 2.1 Participants and study design

Participants were persons living with dementia (total *n *= 166), living either in care homes (CARE subsample, *n *= 75) or at home (HOME subsample, *n *= 91). Inclusion criteria comprised: (1) at least mild cognitive impairment (Clinical Dementia Rating [CDR] score ≥ 0.5) caused by a diagnosed progressive neurocognitive disorder, (2) age ≥ 60 years, (3) fluency in Finnish, (4) no history of severe psychiatric disorders (prior to dementia) or substance abuse, (5) no changes to psychotropic medication within 3 months, and (6) sufficient sensory and cognitive functioning to enable participation in intervention groups. The study was approved by the ethics committee of the Hospital District of Helsinki (HUS/5459/2024). All participants gave informed consent, either in written format or orally (witnessed by researcher and care staff member). To ensure its maintenance, consent was verified orally before all outcome assessments.

**FIGURE 1:**
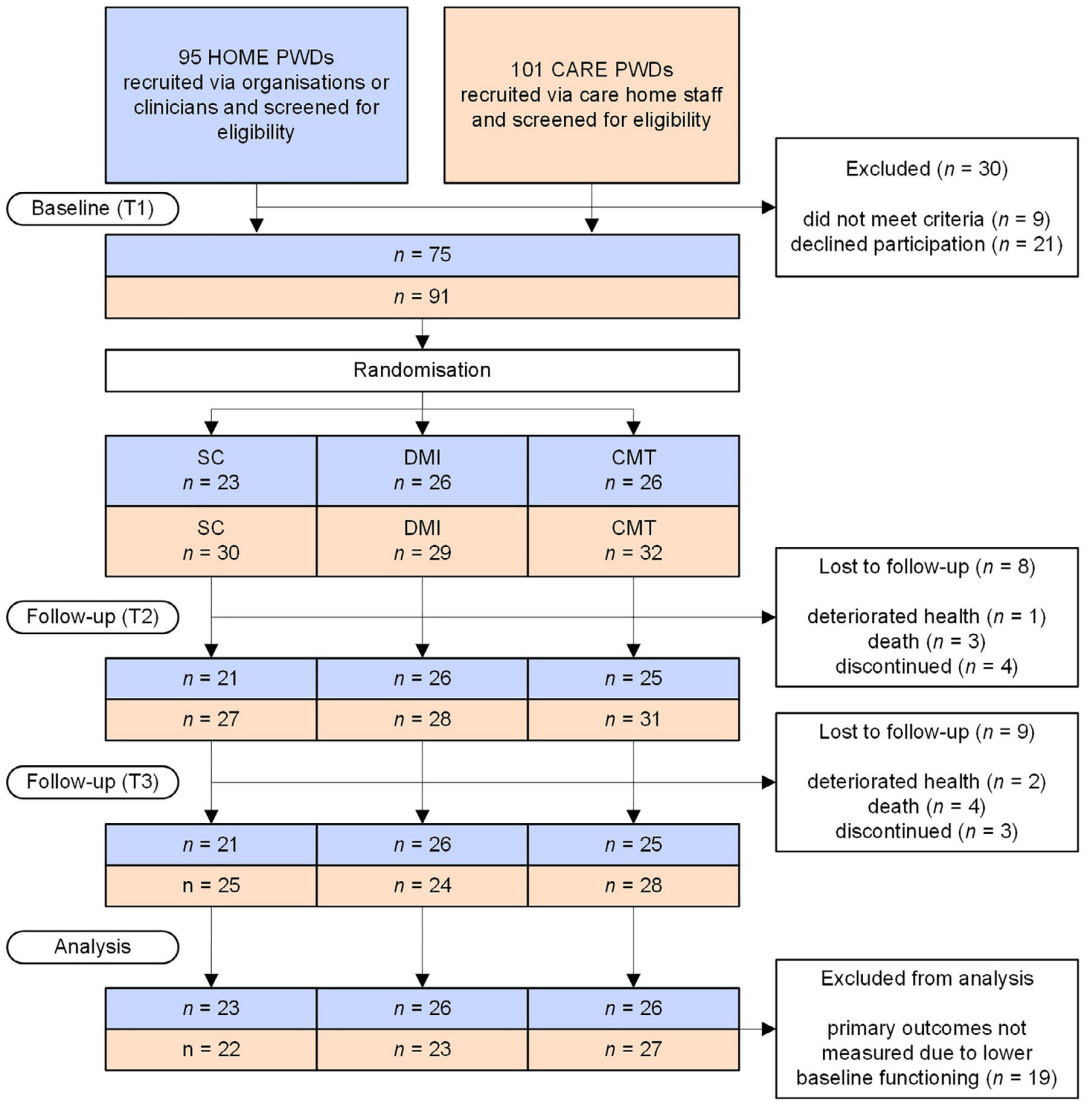
Study design from recruitment to analysis of primary outcome. CARE, participants living in care homes; CMT, conventional music therapy; DMI, digital music intervention; HOME, participants living at home; PWD, person with dementia; SC, standard care.

This exploratory trial followed a three‐arm parallel‐group RCT design (see Figure 1), registered at ClinicalTrials.gov (NCT05520268) on August 27, 2022. A priori calculation using G*Power^17^ indicated that sample size *N *= 156 was needed to detect estimated medium–large effect size (Cohen *f *= 0.28, specified as in Cohen,^18^ based on previous results^8^) with 80% power and 0.05 alpha level for a within–between time (3 measurements) x group (3 groups) interaction in a repeated‐measures analysis of variance. To achieve optimal intervention group sizes (5–8 participants), participants were recruited in blocks of 17 to 24 participants; four CARE blocks from four care homes (enrolled September 2022 to February 2023, January to March 2024), and four HOME blocks recruited from senior centers, outpatient geriatric clinics, and patient organizations (enrolled August to November 2023 and 2024). Participants within each block were randomized with equal probability (1:1:1) to one of three groups (CMT, DMI, SC) by a person not involved in data collection using a random number generator. Randomization was stratified for age, sex, dementia severity (CDR), and etiology. Outcome measures were performed at baseline (T1), end of treatment (T2: 3‐month follow‐up), and long term (T3: 6‐month follow‐up).

### 2.2 Interventions

Music interventions were implemented as group sessions (5–8 participants per group) over a 10‐week period, with two sessions/week and 60 minutes/session, separately for each of the eight blocks.

#### 2.2.1 CMT

Designed by two experienced professional music therapists (S.L. and M.H.), CMT followed a music therapy protocol adapted from a previous study,^8^ in which participants engaged in both receptive and expressive live musical activities using pleasant and autobiographically salient music. Receptive activities (≈ 57% of realized session time on average) included music listening coupled with cognitively stimulating activities (e.g., reminiscence, discussion, recalling song lyrics, painting to music) and relaxation. Expressive activities (43% of session time) included sing‐alongs with live accompaniment and musical movement (e.g., body percussion, dancing) and music making with percussive instruments (e.g., shakers, claves) or other props (e.g., scarves, fabric ring). Session structure was flexible and adapted to participant wishes and needs. CMT was implemented as live in‐person sessions by one music therapist (S.L. or M.H.) who led sessions, creating a safe and validating atmosphere and facilitating communication between participants through therapeutic interaction. A research assistant coordinated practicalities (e.g., liaising with caregivers, session logging/videoing for fidelity). To ensure consistency across facilitators, music therapists alternated facilitation between each block and provided each other peer supervision across the study.

#### 2.2.2 DMI

DMI was designed by an external service provider with extensive experience in geriatric care and in collaboration with the research team and music therapists (S.L. and M.H.), to enhance conformity with CMT. DMI sessions utilized pre‐recorded music‐based video exercises through an online audiovisual content service, which participants followed from a screen. Content aligned with typical music therapy components in dementia care, including both receptive (music listening, reminiscence, discussion, recalling song lyrics, music‐related trivia and song rating; 58% of session time) and expressive (communal singing alongside recorded music or karaoke, musical movement through guided light exercise to music; 42% of session time) musical activities (see Figure S1 in supporting information). DMI was implemented by a trained facilitator who encouraged participants to engage with content and each other, and a research assistant who coordinated practicalities (e.g., solving technical issues, session logging/videoing). DMI was implemented for CARE participants as in‐person sessions with participants and facilitator gathered around the same screen, and for HOME participants as virtual sessions using tablet computers and videoconferencing software, with caregiver assistance when needed. To ensure fidelity, DMI followed a predetermined, fixed structure across each session and each block.

A more detailed description of CMT and DMI is provided in the Supplementary Material in supporting information.

#### 2.2.3 SC

All participants received SC for dementia either through care homes or public/private health care. SC group participants did not receive any additional intervention activities and were instructed to continue their existing daily and leisure activities during the 6‐month study period. After T3, they were given the opportunity to partake in musical activity sessions using the DMI content service.

### 2.3 Outcome measures

General cognition was selected as the primary outcome, given that cognitive decline is a core feature of dementia and previous music intervention trials have demonstrated cognitive benefits for persons living with dementia.^8^, ^9^, ^11^, ^13^, ^14^, ^15^ The primary outcome was measured as changes from T1 to T2 in Montreal Cognitive Assessment (MoCA)^19^ total score and Consortium to Establish a Registry for Alzheimer's Disease (CERAD) Chandler total score.^20^ As standard outcome measures in dementia trials, MoCA and CERAD are feasible across varying dementia severities and provide comprehensive evaluation of the cognitive domains affected in dementia, with MoCA offering a brief screening assessment and CERAD providing more extensive assessment. Secondary outcomes were changes in MoCA and CERAD Chandler total scores from T1 to T3. In addition, secondary outcomes included changes (T1–T2 and T1–T3) in specific cognitive, emotional, and psychosocial measures listed below.

Test‐based assessments were conducted in a research laboratory or home setting (HOME) or in care home units (CARE) by trained research staff who were masked to group allocation. Masking was mostly successful; there were six accidental cases of unmasking by participants. Questionnaires were filled out at home/care home by participants and their caregivers (family members or nursing staff; *n *= 160) using either paper forms or electronic forms (implemented securely via REDCap^21^). If participants required assistance with questionnaires, caregivers were instructed to read questions and answer options without guiding the response.

#### 2.3.1 Cognitive measures

Standardized neuropsychological tests (duration: 1 hour) including MoCA, CERAD, Wechsler Memory Scale IV (WMS‐IV)^22^ subtests, and INECO Frontal Screening (IFS)^23^ were used to evaluate general cognition, verbal skills, visuospatial skills, learning, episodic memory, and executive function (see Table 1). Parallel versions of memory tests were used to minimize learning effects and order of test versions was randomized for each participant. Additionally, AM was evaluated with the Brief Autobiographical Recollection Test (B‐ART)^24^ and custom‐made AM questionnaire and interview (see Supplementary Material).

**TABLE 1:** Outcome measures.

Measure	Description	Outcome variable (min–max)
MoCA	Brief cognitive screening tool on general cognition	Sum (0–30)
CERAD Chandler total	Neuropsychological battery measuring cognitive status	Sum (0–100)
Phonemic fluency	Letter “S” words listed within 60 seconds	Sum (0–)
Categorical fluency	Animals listed within 60 seconds	Sum (0–)
CERAD naming	Naming 15 objects based on line drawings	Sum (0–15)
CERAD copying	Copying four shapes from line drawings	Sum (0–11)
CERAD WL immediate recall	Words recalled from a list of ten in first trial	Sum (0–10)
CERAD WL learning	Words learned by third trial	3rd – 1st trial sum (–10 to 10)
CERAD WL delayed recall	Words recalled after delay	Sum (0–10)
CERAD WL recognition	Word list words recognized from 20 words	Sum (0–20)
WMS‐IV LM immediate recall	Details of WMS‐IV short story recalled in first trial	Sum (0–14)
WMS‐IV LM learning	Details of WMS‐IV short story learned by second trial	2nd – 1st trial sum (–14 to 14)
WMS‐IV LM delayed recall	Details of WMS‐IV short story recalled after delay	Sum (0–14)
IFS total	Short set of cognitive tasks measuring frontal functions	Sum (0–30)
IFS executive function	Executive function task subscale	Sum of scores (0–18)
IFS working memory	Working memory task subscale	Sum (0–12)
AM interview	9 prompts for autobiographical recollection	Sum (0–15)
B‐ART	7 items on richness of recollection, self‐ and proxy ‐rated	Mean of self‐ and proxy‐rating (1–7)
Spontaneous AM	7 items on frequency of spontaneous recollection, self‐ and proxy rated	Mean of self‐ and proxy‐rating (7–35)
Prompted AM	7 items on frequency of prompted recollection, proxy rated	Sum (7–35)
MADRS	10 items on depression, proxy rated	Sum (0–60)
PROMIS Depression	8 items on depression, self‐rated	Standardized sum (37.1–81.1)
PROMIS Anxiety	8 items on anxiety, self‐rated	Standardized sum (37.1–83.1)
NPI‐Q total score	24 items on neuropsychiatric symptoms, proxy rated	Sum of severity x distress items (0–180)
QOL‐AD	15 items on disease‐specific quality of life, self‐ and proxy rated	Mean of self‐ and proxy rating (15–60)
EQ‐5D‐5L	5 items on quality of life, self‐ and proxy rated	Mean of self‐ and proxy rating (0–1)
EQ‐VAS	Self‐ and proxy‐rated visual analogue scale on current health	Mean of self‐ and proxy rating (0–100)
GHQ	12 questions on general well‐being	Sum (12–48)
ZBI	12 questions on caregiver burden	Sum (12–60)

Abbreviations: AM, autobiographical memory; B‐ART, Brief Autobiographical Recollection Test; CERAD, Consortium to Establish a Registry for Alzheimer's Disease; EQ‐5D‐5L, EuroQol 5‐level 5D version; EQ‐VAS, EuroQol Visual Analogue Scale; GHQ, General Health Questionnaire; IFS, INECO Frontal Screening; LM, logical memory; MADRS, Montgomery‐Åsberg Depression Rating Scale; MoCA, Montreal Cognitive Assessment; NPI‐Q, Neuropsychiatric Inventory Questionnaire; PROMIS, Patient‐Reported Outcomes Measurement Information System; QOL‐AD, Quality of Life in Alzheimer's Disease; WL, word list; WMS‐IV, Wechsler Memory Scale, Fourth Edition; ZBI, Zarit Burden Interview.

#### 2.3.2 Emotional and psychosocial measures

Mood, neuropsychiatric symptoms, and QOL were assessed via Montgomery‐Åsberg Depression Rating Scale (MADRS),^25^ Patient‐Reported Outcomes Measurement Information System (PROMIS)^26^ Depression 8b and Anxiety 8a short forms, Neuropsychiatric Inventory Questionnaire (NPI‐Q),^27^ Quality of Life in Alzheimer's Disease (QOL‐AD),^28^ and the EuroQol (EQ) 5‐level 5D version^29^ based on self‐rating (by participants) and/or proxy‐rating (by caregivers) responses (see Table 1). Caregivers also reported on their own well‐being using the General Health Questionnaire (GHQ)^30^ and Zarit Burden Interview (ZBI).^31^ Missing response items for all questionnaires (excluding EQ Visual Analogue Scale) were imputed with respondent mean value when at least 50% of items were present. To minimize missingness and preserve sample size, scores from measures with self‐ and proxy ratings available were averaged and used alongside original self‐ or proxy‐rating scores when only one was available.

#### 2.3.3 Subjective benefits

Participants and their caregivers reported subjective benefits of CMT and DMI via feedback questionnaires (see Supplementary Material) completed at T2. Questionnaires measured quantitative cognitive, emotional, and interpersonal benefits of interventions and benefits for caregiver well‐being and caregiver–participant interaction. Qualitative benefits and feedback on interventions was also collected.

### 2.4 Statistical analysis

All statistical analyses were performed with R version 4.5.1^32^ using base, lme4,^33^ and lmerTest^34^ packages. Graphics were computed using emmeans^35^ and ggplot2.^36^

#### 2.4.1 Primary analysis of intervention outcomes

Following the intention‐to‐treat principle,^37^ primary statistical analyses were conducted using longitudinal linear mixed‐effects models (LMM) including all participants with all available observations and regardless of intervention adherence. To compare CMT and DMI to SC, group x time interactions were analyzed in two separate models. The first model contained data from T1 and T2 timepoints for end of treatment effects and the second contained T1 and T3 data for long‐term effects. These models were fitted to the whole sample and separately to HOME and CARE samples to account for differences in DMI delivery mode in HOME and CARE. For some outcomes, analyses were limited to the whole and HOME samples as many CARE participants were unable or unwilling to complete demanding cognitive tests and complex questionnaire items, resulting in high rates of missingness. LMMs were conducted using timepoint (T1 and T2/T1 and T3) and intervention group (SC, DMI, CMT) as fixed effects and individual variance as a random intercept. T1 data was used as the reference timepoint, thus accounting for baseline differences. Residuals for LMMs were checked visually. Given the exploratory nature of analyses, we used unadjusted α = 0.05 to decrease probability of Type II errors.^38^, ^39^

#### 2.4.2 Sensitivity analysis of intervention outcomes

To evaluate efficacy of DMI and CMT as implemented, primary LMM analyses were repeated in a per‐protocol subsample comprising participants who completed all three timepoints, for DMI and CMT participants, attended ≥ 53.9% (mean – 1 standard deviation [SD]) of intervention sessions. These sensitivity analyses were interpreted as supportive of the primary analyses.

#### 2.4.3 Ancillary analyses comparing intervention groups

To directly compare effects of CMT and DMI, follow‐up analyses between DMI and CMT were conducted for outcomes that showed statistically significant results in primary and sensitivity analyses.

#### 2.4.4 Analysis of subjective benefits

To determine significant benefits experienced by participants and caregivers, quantitative feedback was analyzed with independent two‐way *t* tests, comparing mean benefit scores (Likert scores from 1–10) of each category to a universal mean of 5, which was considered the threshold for substantial benefit. Qualitative feedback was manually reviewed and categorized into recurring themes.

## 3 RESULTS

### 3.1 Group characteristics

Of 166 enrolled participants, 95% (*n *= 158) completed the 3‐month (T2) and 90% (*n *= 149) completed the 6‐month (T3) follow‐up assessment. Confounding variables such as group membership, age, sex, education level, and baseline general cognition did not differ significantly between participants who completed follow‐up and those who dropped out (*n *= 17; see Table S1 in supporting information). On average, dementia severity was higher for participants who dropped out than those who completed follow‐up (*P *= 0.035; see Table S1).

Groups were comparable in demographic and clinical characteristics at T1 (see Table 2) and hence these were not adjusted for in following analyses. In the CARE sample, current instrument playing was higher in CMT than other groups, but this concerned only four participants across three different blocks and was not considered relevant. At T2 and T3, groups were comparable in rehabilitative and leisure activities received outside the study protocol (see Table S2 in supporting information). Average intervention adherence was 79.1% (SD = 25.2%) and was comparable in CMT and DMI.

**TABLE 2:** Participant characteristics by group for the whole sample and subsamples.

Demographic information	Sample	SC	DMI	CMT	*p*
Age	All	80.15 (8.73)	81.25 (9.00)	80.93 (8.45)	.797 (*F*)
	HOME	75.96 (6.79)	75.81 (7.78)	75.69 (4.25)	.990 (*F*)
	CARE	83.37 (8.78)	86.14 (7.06)	85.19 (8.65)	.423 (*F*)
Sex Woman/man/other	All	31/22/0	29/26/0	31/27/0	.807 (*X^2^*)
	HOME	10/13/0	10/16/0	11/15/0	.931 (*X^2^*)
	CARE	21/9/0	19/10/0	20/12/0	.822 (*X^2^*)
Education level	All	3.10 (1.57)	3.04 (1.79)	3.04 (1.75)	.962 (*H*)
	HOME	3.39 (1.27)	3.72 (1.74)	3.46 (1.61)	.647 (*H*)
	CARE	2.86 (1.76)	2.38 (1.60)	2.67 (1.81)	.612 (*H*)
Clinical information					
Dementia etiology (AD/mixed AD/VD/other PND)	All	37/5/4/6	38/7/5/5	38/8/7/5	.966 (*X^2^*)
	HOME	16/4/2/0	17/5/1/3	15/5/4/2	.639 (*Fisher*)
	CARE	21/1/2/6	21/2/4/2	23/3/3/3	.731 (*Fisher*)
Years from symptom onset	All	5.78 (3.90)	6.64 (4.32)	5.82 (3.71)	.451 (*H*)
	HOME	4.71 (3.41)	5.80 (3.80)	4.25 (2.88)	.308 (*H*)
	CARE	6.68 (4.12)	7.61 (4.75)	7.22 (3.85)	.560 (*H*)
CDR	All	1.40 (0.80)	1.25 (0.80)	1.28 (0.77)	.595 (*H*)
	HOME	1.04 (0.69)	0.77 (0.55)	0.94 (0.64)	.325 (*H*)
	CARE	1.67 (0.79)	1.68 (0.72)	1.55 (0.77)	.706 (*H*)
Current leisure activities					
Physical exercise	All	3.02 (1.49)	3.53 (1.36)	3.5 (1.56)	.142(*H*)
	HOME	3.83 (1.23)	4.52 (0.51)	4.42 (0.86)	.055 (*H*)
	CARE	2.31 (1.35)	2.58 (1.24)	2.7 (1.60)	.654(*H*)
Social activity	All	2.92 (1.20)	3.06 (1.14)	2.93 (1.37)	.815 (*H*)
	HOME	3.23 (1.07)	3.35 (0.75)	3.73 (0.96)	.129 (*H*)
	CARE	2.67 (1.27)	2.76 (1.39)	2.23 (1.3)	.231 (*H*)
Reading & writing	All	2.40 (1.06)	2.64 (1.18)	2.48 (1.06)	.397(*H*)
	HOME	2.93 (0.97)	3.37 (1.06)	2.96 (0.68)	.069 (*H*)
	CARE	1.94 (0.92)	1.94 (0.81)	2.08 (1.16)	.926 (*H*)
Art & craft	All	1.48 (0.74)	1.74 (0.91)	1.55 (0.85)	.222 (*H*)
	HOME	1.73 (0.96)	2.00 (0.98)	1.77 (0.86)	.442 (*H*)
	CARE	1.28 (0.42)	1.48 (0.77)	1.34 (0.80)	.383 (*H*)
Music listening	All	3.56 (1.42)	3.62 (1.35)	3.41 (1.51)	.773 (*H*)
	HOME	4.00 (1.17)	4.23 (1.03)	4.19 (1.13)	.752 (*H*)
	CARE	3.19 (1.52)	3.00 (1.36)	2.73 (1.48)	.392 (*H*)
Singing	All	2.44 (1.47)	2.06 (1.22)	2.31 (1.27)	.387 (*H*)
	HOME	2.78 (1.62)	2.40 (1.26)	2.60 (1.35)	.810 (*H*)
	CARE	2.15 (1.29)	1.73 (1.12)	2.07 (1.16)	.256 (*H*)
Instrument playing	All	1.16 (0.42)	1.27 (0.83)	1.34 (0.83)	.659 (*H*)
	HOME	1.36 (0.58)	1.56 (1.12)	1.48 (1.05)	.930 (*H*)
	CARE	1.00 (0.00)	1.00 (0.00)	1.21 (0.57)	.017 (*H*)
Dancing	All	1.72 (1.07)	1.58 (0.94)	1.53 (0.90)	.704 (*H*)
	HOME	1.87 (1.14)	1.85 (1.05)	1.69 (0.84)	.922 (*H*)
	CARE	1.59 (1.01)	1.31 (0.74)	1.38 (0.94)	.407 (*H*)
Musical background					
Singing	All	2.49 (1.84)	2.1 (1.63)	2.25 (1.73)	.667 (*H*)
	HOME	2.48 (1.90)	2.19 (1.60)	2.28 (1.72)	.996 (*H*)
	CARE	2.50 (1.82)	2.00 (1.68)	2.23 (1.77)	.480 (*H*)
Instrument playing	All	1.90 (1.43)	1.80 (1.41)	2.09 (1.54)	.543 (*H*)
	HOME	2.50 (1.68)	2.44 (1.64)	2.16 (1.43)	.812 (*H*)
	CARE	1.43 (1.00)	1.19 (0.80)	2.03 (1.65)	.052 (*H*)
Dancing	All	2.02 (1.49)	2.39 (1.64)	2.14 (1.67)	.419 (*H*)
	HOME	1.86 (1.42)	2.58 (1.79)	2.50 (1.75)	.274 (*H*)
	CARE	2.15 (1.56)	2.20 (1.47)	1.83 (1.56)	.305 (*H*)
Musical sophistication	All	50.00 (12.19)	46.37 (9.94)	45.39 (11.63)	.142 (*H*)
	HOME	52.09 (11.68)	50.67 (9.46)	49.58 (11.58)	.799 (*H*)
	CARE	48.29 (12.54)	42.22 (8.67)	41.87 (10.62)	.093 (*H*)
Music reward sensitivity	All	63.78 (13.27)	66.01 (12.73)	62.83 (15.53)	.584 (*H*)
	HOME	64.8 (13.30)	69.06 (12.53)	67.35 (15.83)	.516 (*H*)
	CARE	62.97 (13.42)	63.07 (12.46)	59.05 (14.46)	.539 (*H*)
Emotional responsiveness to music	All	3.04 (0.83)	3.02 (0.91)	2.78 (0.93)	.301 (*H*)
	HOME	2.94 (0.85)	3.24 (0.98)	2.96 (0.95)	.500 (*H*)
	CARE	3.13 (0.82)	2.81 (0.80)	2.63 (0.91)	.055 (*H*)

*Notes*: Data are mean (standard deviation) unless otherwise stated. Education level was measured on a 6‐point scale from 1 (primary education) to 6 (doctoral education). CDR is scored on a 5‐point scale from 0 (no cognitive impairment) to 3 (severe dementia). Current leisure activities were measured on a 5‐point scale from 1 (never) to 5 (daily). Musical background variables were measured on a 5‐point scale from 1 (no experience) to 5 (10+ years of experience). Musical sophistication was measured with abbreviated versions of the Perceptual and Singing Abilities subscales of the Goldsmiths Musical Sophistication Index (Gold‐MSI) and an abbreviated version of the Goldsmiths Dance Sophistication Index (Gold‐DSI), 15 items were scored on a 5‐point scale from 1 (completely disagree) to 5 (completely agree) and summed. Music reward sensitivity was measured with the Barcelona Music Reward Questionnaire, 20 items were scored on a 5‐point scale from 1 (completely disagree) to 5 (completely agree) and summed. Emotional responsiveness to music was measured with an abbreviated version of the Brief Music in Mood Regulation (BMMR) scale, 14 items were scored on a 5‐point scale from 1 (completely disagree) to 5 (completely agree) and averaged. Abbreviated versions of Gold‐MSI/DSI and BMMR are included in the Supplementary Material in supporting information.

Abbreviations: AD, Alzheimer's disease; CARE, participants living in care homes; CDR, Clinical Dementia Rating; CMT, conventional music therapy; DMI, digital music intervention; HOME, participants living at home; PND, progressive neurocognitive disorder; SC, standard care; VD, vascular dementia.

### 3.2 Primary cognitive outcomes

In either primary (intention‐to‐treat) or sensitivity (per‐protocol) analyses, there were no group x time interactions in MoCA or CERAD Chandler total scores in T1–T2 or T1–T3 models (see Table 3). Primary and sensitivity analysis results for primary and secondary outcomes are reported in Supplementary Material and Tables S3 and S4 in supporting information.

**TABLE 3:** Results for primary cognitive outcomes from primary analyses.

		n, Mean (SD)			*p*	Interaction *β* [95% CI], *p*	
Variable	Group	T1	T2	T3	T1 diff.	T1–T2	T1–T3
All							
MoCA total	SC	45, 13.09 (5.88)	43, 12.67 (6.29)	40, 12.97 (6.70)	.642		
	DMI	49, 11.71 (7.28)	47, 11.98 (7.83)	44, 12.23 (7.95)		0.09 [–1.04, 1.21], .878	−0.35 [−1.61, 0.90], .579
	CMT	53, 12.28 (7.55)	50, 12.66 (7.60)	41, 12.54 (7.27)		0.40 [–0.71, 1.51], .475	−0.50 [−1.77, 0.77], .437
CERAD Chandler total	SC	36, 46.25 (14.03)	31, 46.42 (18.75)	29, 47.24 (17.45)	.773		
	DMI	35, 48.00 (19.80)	33, 48.55 (20.65)	33, 46.94 (20.92)		0.50 [–3.65, 4.65], .812	−0.94 [−5.07, 3.18], .650
	CMT	35, 49.00 (18.83)	33, 49.64 (17.42)	27, 48.89 (19.32)		0.83 [–3.32, 4.98], .692	−0.03 [−4.35, 4.30], .991
HOME							
MoCA total	SC	23, 15.87 (5.32)	21, 15.29 (6.37)	21, 16.19 (6.53)	.968		
	DMI	26, 16.00 (6.42)	26, 16.31 (7.58)	26, 15.92 (7.56)		1.05 [–0.74, 2.84], .247	−0.24 [−2.06, 1.58], .794
	CMT	26, 15.88 (6.45)	25, 16.08 (6.81)	23, 15.52 (6.50)		1.12 [–0.69, 2.93], .221	−0.63 [−2.50, 1.25], .506
CERAD Chandler total	SC	23, 51.13 (14.08)	21, 52.10 (19.40)	21, 51.10 (18.50)	.644		
	DMI	25, 54.12 (18.72)	25, 54.08 (19.58)	25, 53.20 (19.36)		−0.66 [−5.82, 4.50], .799	−0.54 [−5.56, 4.48], .831
	CMT	24, 54.58 (16.47)	23, 53.43 (16.55)	20, 54.45 (17.10)		−1.79 [−7.05, 3.47], .499	−0.69 [−5.98, 4.60], .795
CARE							
MoCA total	SC	22, 10.18 (5.05)	22, 10.18 (5.21)	19, 9.42 (4.94)	.125		
	DMI	23, 6.87 (4.73)	21, 6.62 (3.84)	18, 6.89 (4.96)		−1.03 [−2.36, 0.29], .124	−0.66 [−2.36, 1.04], .436
	CMT	27, 8.81 (6.96)	25, 9.24 (6.86)	18, 8.72 (6.51)		−0.36 [−1.63, 0.91], .575	−0.43 [−2.12, 1.27], .617

*Notes*: T1 = baseline; T2 = 3‐month follow‐up; T3 = 6‐month follow‐up; T1–T2 = change from T1 to T2; T1–T3 = change from T1 to T3.

Abbreviations: AD, Alzheimer's disease; CARE, participants living in care homes; CERAD, Consortium to Establish a Registry for Alzheimer's Disease; CI, confidence interval; CMT, conventional music therapy; DMI, digital music intervention; HOME, participants living at home; MoCA, Montreal Cognitive Assessment; SC, standard care.

### 3.3 Secondary cognitive, emotional, and psychosocial outcomes

**FIGURE 2:**
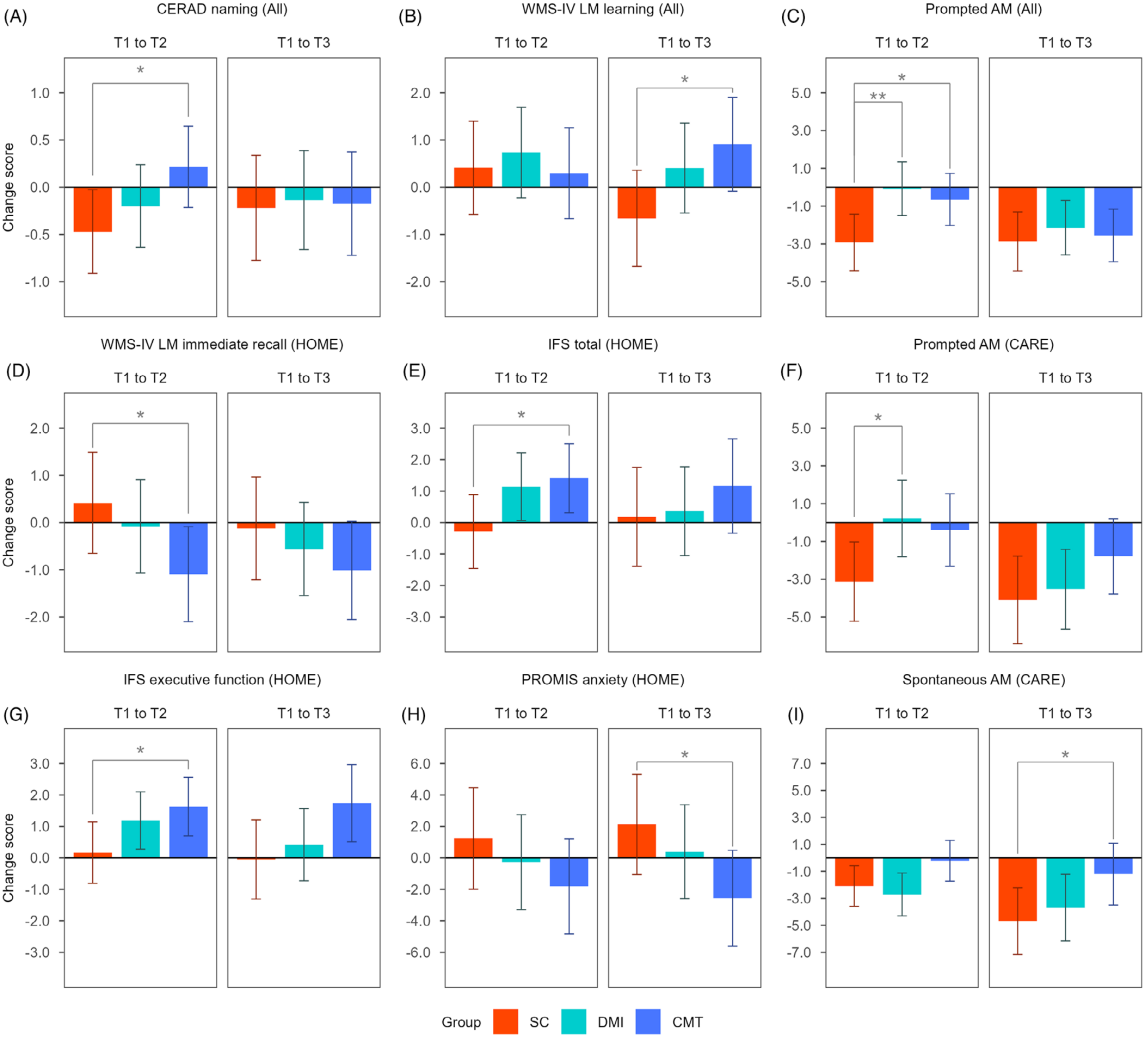
Changes in cognitive and emotional outcomes at end of treatment and long term. Note that the presented change scores were calculated solely for visualization purposes. The change score values are the estimated marginals means of change from T1 to T2/T3 ± standard error of mean according to the relevant linear mixed‐effects model. Estimated marginal means were used from primary analysis for (A), (B), (C), (D), (E), and (F) and from sensitivity analysis for (G), (H), and (I). * = *P <* 0.05, ** = *P <* 0.01. AM, autobiographical memory; CARE, participants living in care homes; CERAD, Consortium to Establish a Registry for Alzheimer's Disease; CMT, conventional music therapy; DMI, digital music intervention; HOME, participants living at home; IFS, INECO Frontal Screening; LM, logical memory task; PROMIS, Patient‐Reported Outcomes Measurement Information System; SC, standard care; WMS‐IV, Wechsler Memory Scale IV.

All statistically significant LMM results in secondary outcomes are illustrated in Figure 2. In primary analyses, there were group x time interactions in (1) CERAD naming (CMT > SC, T1–T2), WMS‐IV learning (CMT > SC, T1–T3), and prompted AM (CMT > SC and DMI > SC, T1–T2) in the whole sample; (2) IFS total (CMT > SC, T1–T2) and WMS‐IV immediate recall (CMT < SC, T1–T2) for HOME; and (3) prompted AM (DMI > SC, T1–T2) for CARE. Effects were approximately small/medium in size (Cohen *d *= 0.18–0.66; see Table S5 in supporting information).

In sensitivity analyses, the effects observed in primary analyses remained significant for CERAD naming, WMS‐IV immediate recall, IFS total, and prompted AM (whole sample), but not for WMS‐IV learning. In the CARE sample, the effect of DMI on prompted AM did not remain significant, but CMT reached significance (CMT > SC, T1–T2). Additionally, there were group x time interactions in (1) IFS executive (CMT > SC, T1–T2) and PROMIS Anxiety (CMT < SC, T1–T3) for HOME and (2) spontaneous AM (CMT > SC, T1–T3) for CARE. Effects were approximately small/medium in size (Cohen *d *= 0.11–0.62; see Table S5).

While most significant effects were observed in CMT compared to SC, ancillary follow‐up analyses showed that there were no significant differences between changes in DMI and CMT in any of the significant effects observed in the primary/sensitivity analyses (see Table S6 in supporting information).

#### 3.3.1 Subjective benefits

Based on significant effects observed in *t* tests for quantitative feedback (see Table 4), (1) participants reported substantial cognitive, emotional, and social benefits from both DMI and CMT in whole, HOME, and CARE samples; (2) caregivers reported emotional benefits from both DMI and CMT and social benefits from CMT in whole and HOME samples; and (3) caregivers reported emotional benefits for participants from CMT and benefits for communication with participants from DMI in the CARE sample.

**TABLE 4:** Results of subjective benefit *t* tests on quantitative feedback.

			DMI			CMT		
Respondent	Sample	Category	*n*	Mean [95% CI]	*p*	*n*	Mean [95% CI]	*p*
Persons living with dementia	All	Cognitive	41	6.8 [6.15, 7.46]	<.001	34	7.29 [6.56, 8.03]	<0.001
		Emotional	43	7.44 [6.85, 8.04]	<.001	35	8.63 [8.24, 9.02]	<.001
		Social	39	6.54 [5.79, 7.29]	<.001	32	8.06 [7.51, 8.61]	<.001
	HOME	Cognitive	23	6.35 [5.32, 7.38]	.013	18	7.11 [5.84, 8.39]	.003
		Emotional	23	7.13 [6.12, 8.14]	<.001	17	8.41 [7.75, 9.07]	<.001
		Social	23	5.83 [4.72, 6.93]	.135	18	7.94 [7.17, 8.72]	<.001
	CARE	Cognitive	18	7.39 [6.64, 8.14]	<.001	16	7.5 [6.7, 8.3]	<.001
		Emotional	20	7.8 [7.2, 8.4]	<.001	18	8.83 [8.34, 9.32]	<.001
		Social	16	7.56 [6.81, 8.32]	<.001	14	8.21 [7.33, 9.1]	<0.001
Caregivers	All	Cognitive	40	4.58 [3.61, 5.54]	.380	43	5.3 [4.39, 6.22]	.508
		Emotional	40	6.38 [5.37, 7.38]	.008	44	6.89 [6.06, 7.72]	<0.001
		Social	41	5.2 [4.18, 6.21]	.700	42	6.45 [5.63, 7.28]	.001
		Psychological	29	5.72 [4.45, 7]	.254	40	4.7 [3.69, 5.71]	.550
		Interaction	30	5.9 [4.75, 7.05]	.119	42	5.21 [4.24, 6.19]	.659
	HOME	Cognitive	36	4.58 [3.58, 5.59]	.406	31	5.58 [4.44, 6.72]	.308
		Emotional	36	6.14 [5.06, 7.22]	.039	32	6.91 [5.89, 7.93]	.001
		Social	36	5.06 [3.97, 6.14]	.918	30	6.73 [5.71, 7.76]	.002
		Psychological	25	5.84 [4.52, 7.16]	.201	29	5.14 [3.9, 6.37]	.821
		Interaction	25	5.88 [4.62, 7.14]	.161	31	5.77 [4.61, 6.94]	.184
	CARE	Cognitive	20	4.3 [2.69, 5.91]	.376	29	4.76 [3.57, 5.95]	.682
		Emotional	20	6.6 [4.92, 8.28]	.061	29	6.48 [5.33, 7.63]	.013
		Social	21	5.33 [3.69, 6.98]	.677	28	5.89 [4.76, 7.02]	.116
		Psychological	10	6.8 [4.35, 9.25]	.131	24	3.71 [2.31, 5.1]	.068
		Interaction	11	6.91 [5.02, 8.8]	.048	26	4.62 [3.3, 5.93]	.553

*Notes*: Means reported are the means of subjective benefits scored on a scale from 1 (no benefit) to 10 (very beneficial) and collected at T2 (3‐month follow‐up). *p* values indicate the statistical significance between the difference of the mean subjective benefit score to a universal mean of 5.

Abbreviations: CARE, participants living in care homes; CI, confidence interval; CMT, conventional music therapy; DMI, digital music intervention; HOME, participants living at home.

Qualitative feedback from participants and caregivers supported quantitative findings (see Supplementary Material). Participants in HOME and CARE samples appreciated music itself, communality, and novelty of the activity in both DMI and CMT, singing especially in CMT, and visual content and accessibility in DMI. In addition, some HOME participants experienced technical issues (low audio quality, connection issues) and lack of meaningful connection with other participants and facilitators in the online DMI. Care home staff found both interventions to enhance engagement with participants.

There were seven cases of unrelated serious adverse events during the study (three deaths in DMI, one death in CMT, three deaths in SC). Across the whole study, research assistants reported eight unrelated and one related non‐serious adverse events during group sessions (see Table S7 in supporting information).

## 4 DISCUSSION

This RCT investigated the efficacy of DMI across the dementia care continuum alongside CMT and SC. We did not observe an effect of DMI or CMT on the primary outcomes, compared to SC in either primary or sensitivity analyses. Concerning secondary outcomes, results from primary analyses showed that compared to SC, prompted AM was maintained by DMI and CMT in the whole sample and DMI in the CARE sample, and CMT enhanced naming and verbal learning in the whole sample and executive functioning in the HOME sample. These effects remained significant in sensitivity analyses, except for verbal learning and prompted AM. We also observed some effects only in sensitivity analyses, indicating dependency on data completeness and intervention adherence: compared to SC, CMT decreased anxiety in the HOME sample and maintained prompted and spontaneous AM in the CARE sample. Compared directly to each other, DMI and CMT effects did not differ significantly. Overall, our findings align with previous evidence of cognitive and emotional benefits from conventional and digital interventions involving music,^8^, ^9^, ^10^, ^11^, ^12^, ^13^, ^14^, ^15^, ^16^ but provide novel observations that persons living with dementia across the care continuum can benefit from music interventions.

While prior evidence shows cognitive benefits of music interventions in dementia,^8^, ^9^, ^11^ we did not observe significant change in general cognition after DMI or CMT. However, across primary and sensitivity analyses, we observed some improvement in verbal skills (naming) and executive function after CMT and maintenance of AM after DMI and CMT, aligning with prior evidence. This inconsistency in primary and secondary outcomes may be partially explained by characteristics of our primary outcome measures. Despite allowing clinically relevant interpretation and comparison to prior dementia studies, recent work has suggested that commonly used measures of general cognition (e.g., MoCA, Alzheimer's Disease Assessment Scale Cognitive subscale) may be less sensitive to change than domain‐specific measures in clinical trials,^40^, ^41^ especially in a broad dementia samples (ranging from mild cognitive impairment/mild to severe) and with relatively short follow‐up times. However, we observed an increase in verbal learning in primary but not sensitivity analyses and a decrease in immediate verbal recall after CMT, aligning with the null results on general cognition. As our sample comprises mostly persons living with AD (see Table 2) and all groups show long‐term decrease in immediate recall (see Figure 2), this result may reflect underlying disease progression and possibly an acute increase in cognitive load after therapy.

Concerning secondary emotional and psychosocial outcomes, we observed a decrease in anxiety after CMT long term in the sensitivity analyses. While not observed in primary analyses, this result aligns with the current literature demonstrating that music interventions can decrease anxiety or related neuropsychiatric symptoms (agitation, aggression) in dementia,^11^, ^12^, ^13^ with greater benefits of live over online interventions.^13^ Observing this effect specifically in the HOME sample aligns with studies demonstrating non‐pharmacological interventions to support emotional well‐being in early‐stage dementia.^2^ Despite aligning with existing evidence, this effect on anxiety requires further investigation due to dependency on receiving CMT as implemented.

The effects of music interventions on AM are promising. In both primary and sensitivity analyses, we observed maintenance of prompted AM at end of treatment in DMI and CMT compared to a decline in SC, likely reflecting subtle acute effects. In sensitivity analyses, we also observed maintenance of spontaneous AM long term in CMT compared to a decline in SC, possibly reflecting sustained effects of receiving CMT as implemented. These findings align with research suggesting that music can evoke AM even in neuropathological memory loss.^6^, ^7^, ^8^ Taken together with observations in anxiety and verbal skills, these findings also align with theory on music‐evoked AM, which suggests that reduced anxiety and enhanced verbal skills may mediate access to AM in dementia.^7^, ^42^, ^43^ Given the variability of AM effects across analyses/samples, future research should investigate AM further, as a potential mechanism for well‐being in dementia, especially considering the theoretical interplay of music‐evoked AM with other cognitive and emotional functions.

As most intervention effects were observed after CMT, the therapeutic characteristics of CMT may have led to a greater number of effects than DMI. This result aligns with research demonstrating that the therapeutic relationship is a crucial factor for the outcome^44^, ^45^, ^46^ and that therapist‐led music interventions offer more flexibility, individualization, and opportunities for social interaction than interventions using pre‐recorded music.^47^, ^48^ Importantly, although DMI was designed to include typical music therapy components used in dementia care, the music interventions differ in setting, structure, activities, and materials (see Supplementary Material), which complicate comparison. For example, expressive and receptive activities were similar in realized quantity, yet they may have engaged participants differently (e.g., painting to music vs. music trivia). In addition, due to differing settings within the DMI arm (in‐person for CARE, online for HOME), a lack of effects from DMI in the HOME sample may reflect the constraints of total online delivery. While we cannot deduce non‐inferiority from our study, we did not observe significant differences between DMI and CMT in an exploratory comparison across the effects. Quantitative and qualitative feedback was also mostly comparable for CMT and DMI, suggesting similar subjective benefits and supporting results of the primary/sensitivity analyses.

This study contains limitations. Although the missingness of data for CARE participants restricted analyses on subsamples, we conducted a substantial number of analyses, which increased risk of Type I error. Specifically, we analyzed 20 cognitive measures and found statistically significant effects on 7 (35%) measures. However, given the progressive nature of dementia, relatively short intervention and follow‐up periods are unlikely to yield substantive changes. Further increasing risk of Type II error, the analyses may be complicated by improvement in the SC group (see Tables S3–S4) possibly attributed to general supportive aspects of participation, as shown by prior studies in dementia.^49^, ^50^ Finally, without objective session‐level data (participation, time‐on‐task, technical failures), we cannot distinguish between intervention efficacy and differences in actual exposure, especially for HOME participants. Despite these limitations, our findings were theoretically coherent and consistent with prior evidence^8^, ^9^, ^10^, ^11^, ^12^, ^13^, ^14^, ^15^, ^16^ and, while modest and exploratory, can inform future hypotheses through potential meaning for persons living with dementia. However, how well our outcome measures translate to everyday life remains unclear, highlighting the need for ecologically valid functional assessments in future studies.

In conclusion, we did not observe benefits in general cognition after CMT or DMI, contrary to our expectations and previous research. However, our results from exploratory secondary outcomes provide novel observations across the dementia care continuum, which support existing theory and evidence on cognitive and emotional benefits of music for persons living with dementia. Considering the ecological value of different delivery methods, the approaches explored in this study seem to offer distinct advantages. CMT seems to show a greater number of intervention effects compared to SC, whereas the current DMI primarily offers availability and accessibility. To further investigate the efficacy of music interventions with different delivery modes, future studies should examine the relationship between demographic, clinical, and musical background factors and intervention outcomes, given that individual differences likely moderate the therapeutic outcome. Overall, our findings support existing evidence on utilizing music in dementia care yet warrant further investigation to optimize interventions.

## CONFLICT OF INTEREST STATEMENT

The authors declare no conflicts of interest for this article. Author disclosures are available in the Supporting Information.

## CONSENT STATEMENT

All participants gave informed consent, either in written format or orally (witnessed by researcher and care staff member). To ensure its maintenance, consent was verified orally before all outcome assessments.

## Supporting information



Supporting Information

Supporting Information
