# Supplementary material for: Digital music intervention and music therapy across the dementia care continuum: Randomized controlled trial
Source: Alzheimers Dement (N Y). 2026 Aug 31;12(3):e70317. doi: 10.1002/trc2.70317 (PMC13529809; doi:10.1002/trc2.70317)
Supplement: Supplementary file 2 [file TRC2-12-e70317-s002.docx]

**SUPPLEMENTARY MATERIAL**

| Figure S1. Visualisation of digital music intervention video exercises…………………… | 1 |
| --- | --- |
| Description of DMI and CMT………………………………………………………………………….. | 2 |
| Summary of main characteristics of CMT and DMI……………………………………………. | 4 |
| Reporting Guidelines for Music-Based Interventions checklist……………………………. | 5 |
| Custom-made autobiographical questionnaire for caregivers…………………………….. | 6 |
| Custom-made autobiographical interview questions/prompts…………………………… | 7 |
| Feedback questionnaire for participants…………………………………………………………. | 8 |
| Feedback questionnaire for family members of participants………………………………. | 9 |
| Feedback questionnaire for care home staff…………………………………………………….. | 10 |
| Table S1. Characteristics of participants who completed the study period versus participants who dropped out………………………………………………………………………… | 12 |
| Abbreviated Goldsmiths Musical and Dance Sophistication Indices……………………. | 13 |
| Abbreviated Brief Music in Mood Regulation scale……………………………………………. | 14 |
| Table S2. Leisure activity and rehabilitation for HOME and CARE participants across the study period………………………………………………………………………………… | 15 |
| Table S3. Primary analyses for all outcomes by subsample………………………………… | 16 |
| Table S4. Sensitivity analyses for all outcomes by subsample…………………………….. | 26 |
| Table S5. Approximate effect sizes for primary and sensitivity analyses ………………. | 36 |
| Table S6. Follow-up LMM analyses comparing DMI and CMT on statistically significant main analysis effects…………………………………………………………………….. | 41 |
| Description of qualitative feedback from participants and caregivers………………….. | 42 |
| Table S7. Adverse events in group sessions as reported by research assistants…….. | 43 |

**FIGURE S1.** Visualisation of digital music intervention video exercises.

NOTE. The top left image depicts a singing video exercise with accompaniment. The top right image depicts the beginning of a music rating competition exercise. The bottom left image depicts a guided musical time journey video. The bottom right image depicts a guided light exercise video.

**Description of DMI and CMT.**

**Conventional music therapy (CMT)**

CMT was implemented by professional music therapists as live group sessions for HOME and CARE blocks. To ensure consistency in facilitation, the two music therapists alternated facilitation between each block and provided peer supervision for each other across each block. The goal of the intervention was to enable participants to access and interact with familiar, autobiographically salient, and pleasant music through receptive and expressive musical activities. Receptive musical activities included music listening, reminiscing, and discussing, and expressive musical activities included singing, playing, and musical movement. The music was selected by the two music therapists based on their prior clinical experience and knowledge of old-time Finnish songs (e.g. folk songs, popular songs) that are commonly familiar, enjoyable, and often also autobiographically salient in the target population (elderly persons living with dementia), as well as in collaboration with the participants (see below).

While following the predefined protocol for the intervention, the structure of CMT sessions was flexible according to participant wishes, ideas, and thoughts. Fidelity was monitored with structured sessions logs and video recordings across three sessions at the start, middle, and end of each block (approximately sessions 3, 9, and 18). On average, each session started and ended with a greeting song. Otherwise, sessions included sing-alongs, musical movement (dancing, body percussion), and making music with instruments or other props (scarves, fabric ring), cognitive stimulation by listening/singing/playing/painting to music and reminiscing, discussing music, and recalling song lyrics. Occasionally sessions also included relaxation with light touch or dancing. The music therapist ensured a safe and validating atmosphere and facilitated contact and communication between participants throughout each session.

The first sessions (approximately sessions 1-9) of each block were focused on getting to know each other and creating a safe space. Mid-point sessions (approximately sessions 9-15) centred around each participant alternately, for which participants chose and made lists of music outside the group sessions and shared memories related to music during the session. If a participant was unable to access or choose music before the group session, music therapists facilitated choices and reminiscence during the sessions. The last sessions (approximately sessions 15-20) were focused on ending the intervention period, which comprised creating lists of autobiographically salient music and discussing themes chosen freely by the participants (e.g. the future, identity).

**Digital music intervention (DMI)**

DMI was implemented by a trained facilitator using an audiovisual content service aimed at helping e.g. care home staff organise accessible activities for elderly residents. The study included two facilitators across all blocks, one for block 1 who trained the second facilitator. The second facilitator implemented the intervention for blocks 2-8 to maintain facilitator consistency across blocks. DMI was implemented in-person for CARE blocks, and online for HOME blocks. CARE participants gathered with the facilitator in a private room around a TV screen, which was controlled by the facilitator. HOME participants were provided with tablet computers, which were called from a central control laptop computer at the beginning of each session. Participants responded to the call with one button to join the session. The goal and music-based content of DMI corresponded to the receptive and expressive musical elements used in CMT.

The DMI sessions followed a predefined, consistent structure within each block and across the blocks, with variation in specific video exercises used in every session. Fidelity was monitored with structured sessions logs and video recordings across three sessions at the start, middle, and end of each block (approximately sessions 3, 9, and 18). On average, each session included communal singing along recorded musical performance or karaoke, musical movement through guided light exercise to music, and cognitive stimulation through music-related trivia, discussing and reminiscing via music, and recalling song lyrics. The song material used in DMI was largely comparable to the material used in CMT. The content service included specific functionalities to aid music-related reminiscence and discussion. The service included a functionality in which the facilitator entered the birth year (up to 1959) of a participant, after which songs popular across different years in their youth were played alongside photographs from the same time. This functionality was used with communal years relevant to each block or the birth year of each participant in a block. The service also included a music rating competition, in which participants listened to and graded multiple songs. The winning song was revealed at the end. On a few occasions, sessions included more structured music listening with recorded concerts according to group preferences.

**Summary of main characteristics of CMT and DMI.**

|  | CMT | DMI |
| --- | --- | --- |
| Delivery mode | Live, therapist-led group sessions | Group sessions using pre-recorded video exercises |
| Facilitator | Professional music therapist: Two, alternating between blocks Research assistant: logistics & logging | Trained facilitator: One for block 1, one for blocks 2-8 Research assistant: logistics & logging |
| Setting | In-person, in a designated and private room at care home/senior centre | HOME: videoconferencing  CARE: In-person, in a designated and private room at care home/senior centre |
| Dose | 10 weeks, 2 sessions/week, 60 minutes/session | 10 weeks, 2 sessions/week, 60 minutes/session |
| Structure | Following flexible protocol, adapted to participant needs/preferences | Fixed session structure |
| Receptive activities | Music listening, reminiscence, recalling lyrics, relaxation, creative activities (painting to music) | Music listening, reminiscence, recalling lyrics, music trivia, song rating |
| Expressive activities | Communal singing with live accompaniment, musical movement or dance with instruments or props | Communal singing with recorded/karaoke tracks, guided musical movement |
| Materials | Live instruments, props managed by therapist | HOME: Tablet, CARE: Television screen  Audiovisual content service managed by facilitator in both settings |
| Fidelity | Monitored via session logs and videos. | Monitored via session logs and videos. |

NOTE. CMT = conventional music therapy. DMI = digital music intervention. HOME = persons living with dementia, living at home. CARE = persons living with dementia, living in care homes.

**Reporting Guidelines for Music-based Interventions Checklist**

| Item no. | Item | Location |
| --- | --- | --- |
| 1 | **Brief Name.**  Provide the name or phrase that describes the intervention. | Manuscript section 1  Supplementary Material page 2-3 |
| 2 | **Intervention Theory and/or Scientific Rationale.**  Provide a rationale for the music and/or music experience(s). Specify how essential features of the music and music experience(s) are expected to influence targeted outcomes. | Manuscript section 1, 2.2.1, 2.2.2 |
| 3 | **Intervention** **Content.** |  |
| 3a | **Music Selection.** Describe the process for how music was selected including who was involved in music selection. | Supplementary Material pages 2-3 |
| 3b | **Music.** Specify key details about the music that may be relevant to specified outcomes of interest. Characteristics may include compositional features of the music sound intensity or volume, lyrics, and/or how the music relates to the participants’ cultural identity and heritage. When using published music, provide reference for a sound recording or sheet music. | Supplementary Material pages 2-3 |
| 3c | **Music Delivery Method.** Provide details about how music was provided to or created with participants. Include any details necessary for replication. This might include size of performing group, use of playback equipment, person controlling volume. | Manuscript section 2.2.1, 2.2.2  Supplementary Material pages 2-4 |
| 3d | **Materials.** List all materials necessary for the music experience. Include music and non-music equipment and materials. | Manuscript section 2.2.1, 2.2.2  Supplementary Material page 2-4 |
| 3e | **Intervention Strategies.** Describe the music intervention strategy or strategies being studied. | Supplementary Material page 2-3 |
| 4 | **Interventionist.** Specify interventionist qualifications, credentials, training, and/or experience. Indicate how many interventionists delivered the music experience. | Manuscript section 2.2.1, 2.2.2  Supplementary Material page 2-4 |
| 5 | **Individual or Group Intervention.** Specify whether interventions were delivered to individuals or groups of individuals. For group interventions, specify the size of the group. | Manuscript section 2.2 |
| 6 | **Setting.** Describe where the intervention was delivered. Include location, privacy level, ambient sound, and/or any other factors that may have affected participants’ experiences. | Manuscript section 2.2.1, 2.2.2  Supplementary Material page 2-4 |
| 7 | **Intervention Delivery Schedule.** Report number of sessions, session length, frequency, time interval between sessions, and duration. Include practice, experiences, or tasks that are assigned to participants between intervention sessions. | Manuscript section 2.2. |
| 8 | **Treatment Fidelity.** Describe strategies and/or measures used to ensure that the music intervention was delivered and received as intended. | Manuscript section 2.2.1, 2.2.2  Supplementary Material page 2-4 |

NOTE. Reproduced from Robb, S. L., Story, K. M., Harman, E., Burns, D. S., Bradt, J., Edwards, E., ... & Springs, S. (2025). Reporting guidelines for music-based interventions checklist: explanation and elaboration guide. *Frontiers in Psychology*, *16*, 1552659, doi:[10.3389/fpsyg.2025.1552659](https://doi.org/10.3389/fpsyg.2025.1552659).

**Custom-made autobiographical questionnaire for caregivers.**

| Do they spontaneously produce memories related to… | Never | Rarely | Fairly rarely | Fairly often | Often |
| --- | --- | --- | --- | --- | --- |
| 1.       Childhood? | ☐ | ☐ | ☐ | ☐ | ☐ |
| 2.       Adolescence/youth? | ☐ | ☐ | ☐ | ☐ | ☐ |
| 3.       Adulthood? | ☐ | ☐ | ☐ | ☐ | ☐ |
| 4.       Experiences from the past year? | ☐ | ☐ | ☐ | ☐ | ☐ |
| 5.       People you/they know? | ☐ | ☐ | ☐ | ☐ | ☐ |
| 6.       Places related to your/their life? | ☐ | ☐ | ☐ | ☐ | ☐ |
| 7.       Events experienced at any time? | ☐ | ☐ | ☐ | ☐ | ☐ |
| Do they produce memories when asked related to… | Never | Rarely | Fairly rarely | Fairly often | Often |
| 8.       Childhood? | ☐ | ☐ | ☐ | ☐ | ☐ |
| 9.       Adolescence/youth? | ☐ | ☐ | ☐ | ☐ | ☐ |
| 10.    Adulthood? | ☐ | ☐ | ☐ | ☐ | ☐ |
| 11.    Experiences from the past year? | ☐ | ☐ | ☐ | ☐ | ☐ |
| 12.    People they know? | ☐ | ☐ | ☐ | ☐ | ☐ |
| 13.    Places related to their life? | ☐ | ☐ | ☐ | ☐ | ☐ |
| 14.    Events experienced at any time? | ☐ | ☐ | ☐ | ☐ | ☐ |

NOTE. Questions 1-7 refer to spontaneous autobiographical memory, and questions 8-14 refer to prompted autobiographical memory. Participants were asked questions 1-7 with prompt “How often are you able to recall memories related to…”. Questions were scored as “Never” = 1 point, “Rarely” = 2 points, “Fairly rarely” = 3 points, “Fairly often” = 4 points, and “Often” = 5 points. Total score for spontaneous autobiographical memory was calculated as the sum of items 1 to 7 and total score for prompted autobiographical memory was calculated as the sum of items 8 to 14. When both were available, the spontaneous autobiographical memory outcome variable was calculated as the mean of participant self-rated and caregiver proxy-rated score, alongside raw self- or proxy-rated scores when only one was available.

**Custom-made autobiographical interview questions/prompts.**

1. What is your place of birth (hometown)?

2. Where was your first school located?

3. Who was your best friend in childhood?

4. How did you usually spend Christmas when you were a child?

5. Describe your first job.

6. Tell me about a trip you have taken.

7. Describe something that happened last Christmas.

8. Tell me about your most recent birthday.

9. What were your childhood summers like?

Questions/prompts were read out loud to participants and their responses were recorded manually on paper. Participants were not given any further prompts or a time limit to respond. The interview was recorded using an audio recording device and recordings were transcribed by research assistants. The first minute of the participant response was considered for scoring. Responses to close-ended questions 1-3 were scored on a 2-point scale; 0 = participant was not able to remember the answer or did not provide relevant information to answer the question, 1 = participant provided a plausible response to the question. Responses to open-ended questions 4-9 were scored on a 3-point scale; 0 = participant was not able to remember the answer or did not provide relevant information to answer the question, 1 = participant response was lacking in detail, 2 = participant provided a detailed response. The autobiographical interview outcome variable was calculated as the sum of scores for individual questions. Question 9 was devised by a participant of the first block at T1 and added to the original eight items.

**Feedback questionnaire for participants.**

1. How beneficial was the music intervention for your **memory and concentration**?

|  | 1 | 2 | 3 | 4 | 5 | 6 | 7 | 8 | 9 | 10 |  |
| --- | --- | --- | --- | --- | --- | --- | --- | --- | --- | --- | --- |

2. How beneficial was the music intervention for your **mood and state of alertness**?

|  | 1 | 2 | 3 | 4 | 5 | 6 | 7 | 8 | 9 | 10 |  |
| --- | --- | --- | --- | --- | --- | --- | --- | --- | --- | --- | --- |

3. How beneficial was the music intervention to you in terms of **communication and interaction with others**?

|  | 1 | 2 | 3 | 4 | 5 | 6 | 7 | 8 | 9 | 10 |  |
| --- | --- | --- | --- | --- | --- | --- | --- | --- | --- | --- | --- |

4. What was the best thing about the music intervention?

5. What was the worst thing about the music intervention?

6. Other feedback or thoughts about the music intervention:

NOTE. Instruction for the questionnaire included a clarification that for questions 1-3, 1 = no benefit at all, 10 = very beneficial.

**Feedback questionnaire for family members of participants.**

1. How beneficial was the music intervention for the **memory and concentration** of the participant?

|  | 1 | 2 | 3 | 4 | 5 | 6 | 7 | 8 | 9 | 10 |  |
| --- | --- | --- | --- | --- | --- | --- | --- | --- | --- | --- | --- |

2. How beneficial was the music intervention for the **mood and state of alertness** of the participant?

|  | 1 | 2 | 3 | 4 | 5 | 6 | 7 | 8 | 9 | 10 |  |
| --- | --- | --- | --- | --- | --- | --- | --- | --- | --- | --- | --- |

3. How beneficial was the music intervention to the participant in terms of **communication and interaction with others**?

|  | 1 | 2 | 3 | 4 | 5 | 6 | 7 | 8 | 9 | 10 |  |
| --- | --- | --- | --- | --- | --- | --- | --- | --- | --- | --- | --- |

4. What kinds of benefits have you noticed in the aforementioned areas?

5. Have you noticed any other benefits of the music intervention for the participant?

6. How beneficial was the music intervention for **your own psychological wellbeing**?

|  | 1 | 2 | 3 | 4 | 5 | 6 | 7 | 8 | 9 | 10 |  |
| --- | --- | --- | --- | --- | --- | --- | --- | --- | --- | --- | --- |

7. How beneficial was the music intervention in terms of **communication and interaction between you and the participant**?

|  | 1 | 2 | 3 | 4 | 5 | 6 | 7 | 8 | 9 | 10 |  |
| --- | --- | --- | --- | --- | --- | --- | --- | --- | --- | --- | --- |

8.

9. What kinds of benefits have you noticed in the aforementioned areas?

10. Have you experienced any other benefits from your family member participating in the music intervention?

11. Other feedback or thoughts about the music intervention:

NOTE. Instruction for the questionnaire included a clarification that for questions 1-3 and 6-7, 1 = no benefit at all, 10 = very beneficial.

**Feedback questionnaire for care home staff.**

*Think of a resident you interact with who participated in the* ***digital music intervention*** *group and respond to the following questions.*

1. How beneficial was music intervention for the **cognitive functioning (e.g.** **memory and concentration)** of the participant?

|  | 1 | 2 | 3 | 4 | 5 | 6 | 7 | 8 | 9 | 10 |  |
| --- | --- | --- | --- | --- | --- | --- | --- | --- | --- | --- | --- |

2.

2. How beneficial was the music intervention for the **emotional well-being (e.g.** **mood and state of alertness)** of the participant?

|  | 1 | 2 | 3 | 4 | 5 | 6 | 7 | 8 | 9 | 10 |  |
| --- | --- | --- | --- | --- | --- | --- | --- | --- | --- | --- | --- |

3.

3. How beneficial was the music intervention to the participant in terms of **social functioning (e.g.** **communication and interaction with others)**?

|  | 1 | 2 | 3 | 4 | 5 | 6 | 7 | 8 | 9 | 10 |  |
| --- | --- | --- | --- | --- | --- | --- | --- | --- | --- | --- | --- |

4.

4. What kinds of benefits have you noticed in the aforementioned areas?

5. Have you noticed any other benefits of the music intervention for the participant?

*Think of a resident you interact with who participated in the* ***conventional music therapy*** *group and respond to the following questions.*

6. How beneficial was the music intervention for the **cognitive functioning (e.g.** **memory and concentration)** of the participant?

|  | 1 | 2 | 3 | 4 | 5 | 6 | 7 | 8 | 9 | 10 |  |
| --- | --- | --- | --- | --- | --- | --- | --- | --- | --- | --- | --- |

7. How beneficial was the music intervention for the **emotional well-being (e.g.** **mood and state of alertness)** of the participant?

|  | 1 | 2 | 3 | 4 | 5 | 6 | 7 | 8 | 9 | 10 |  |
| --- | --- | --- | --- | --- | --- | --- | --- | --- | --- | --- | --- |

8. How beneficial was the music intervention to the participant in terms of **social functioning (e.g.** **communication and interaction with others)**?

|  | 1 | 2 | 3 | 4 | 5 | 6 | 7 | 8 | 9 | 10 |  |
| --- | --- | --- | --- | --- | --- | --- | --- | --- | --- | --- | --- |

9. What kinds of benefits have you noticed in the aforementioned areas?

10. Have you noticed any other benefits of the music intervention for the participant?

11. How beneficial was the music intervention for **your own occupational and psychological wellbeing**?

|  | 1 | 2 | 3 | 4 | 5 | 6 | 7 | 8 | 9 | 10 |  |
| --- | --- | --- | --- | --- | --- | --- | --- | --- | --- | --- | --- |

12. How beneficial was the music intervention in terms of **communication and interaction between you and the participant**?

|  | 1 | 2 | 3 | 4 | 5 | 6 | 7 | 8 | 9 | 10 |  |
| --- | --- | --- | --- | --- | --- | --- | --- | --- | --- | --- | --- |

13.

14. What kinds of benefits have you noticed in the aforementioned areas?

15. Have you experienced any other benefits from residents participating in the music intervention?

16. Other feedback or thoughts about the music intervention:

NOTE. Instruction for the questionnaire included a clarification that for questions 1-3, 6-8, and 12-13, 1 = no benefit at all, 10 = very beneficial.

**TABLE S1.** Characteristics of participants who completed the study period versus participants who dropped out.

|  | Completed follow-up (*n*=149) | Dropped out (*n*=17) | *P* |
| --- | --- | --- | --- |
| Intervention group (SC/DMI/CMT) | 46/50/53 | 7/5/5 | .687 (*X^2^*) |
| Age | 80.90 (8.85) | 79.82 (7.23) | .577 (*T*) |
| Gender (Woman/Man/other) | 85/64/0 | 6/11/0 | .122 (*Fisher*) |
| Education level | 3.03 (1.72) | 3.29 (1.44) | .549 (*T*) |
| General cognition | 12.52 (6.93) | 9.90 (7.23) | .293 (*T*) |
| CDR | 1.26 (0.76) | 1.76 (0.89) | .035 (*T*) |

NOTE. CDR = Clinical Dementia Rating. CMT = conventional music therapy. DMI = digital music intervention. SC = standard care. Data are mean (SD) unless otherwise indicated. Education level was measured on a 6-point scale from 1 (primary education) to 6 (doctoral education). General cognition was measured by Montreal Cognitive Assessment scores at baseline.

**Abbreviated Goldsmiths Musical and Dance Sophistication Indices.**

| Perceptual abilities | Strongly disagree | Somewhat disagree | Neither disagree/agree | Somewhat agree | Strongly agree |
| --- | --- | --- | --- | --- | --- |
| 1. I can tell when people sing or play out of time with the beat. | 🞏 | 🞏 | 🞏 | 🞏 | 🞏 |
| 2. I can tell when people sing or play out of tune. | 🞏 | 🞏 | 🞏 | 🞏 | 🞏 |
| 3. I find it difficult to spot mistakes in a performance of a song even if I know the tune. | 🞏 | 🞏 | 🞏 | 🞏 | 🞏 |
| 4. When I sing, I have no idea whether I'm in tune or not. | 🞏 | 🞏 | 🞏 | 🞏 | 🞏 |
| 5. I have trouble recognizing a familiar song when played in a different way or by a different performer. | 🞏 | 🞏 | 🞏 | 🞏 | 🞏 |
| Singing |  |  |  |  |  |
| 6. If somebody starts singing a song I don't know, I can usually join in. | 🞏 | 🞏 | 🞏 | 🞏 | 🞏 |
| 7. I am able to hit the right notes when I sing along with a recording. | 🞏 | 🞏 | 🞏 | 🞏 | 🞏 |
| 8. I don’t like singing in public because I’m afraid that I would sing wrong notes. | 🞏 | 🞏 | 🞏 | 🞏 | 🞏 |
| 9. I can sing music from memory. | 🞏 | 🞏 | 🞏 | 🞏 | 🞏 |
| 10. After hearing a new song two or three times, I can usually sing it by myself. | 🞏 | 🞏 | 🞏 | 🞏 | 🞏 |
| Dancing |  |  |  |  |  |
| 11. I find it easy to control my movements. | 🞏 | 🞏 | 🞏 | 🞏 | 🞏 |
| 12. If someone asks me to dance, I usually say yes. | 🞏 | 🞏 | 🞏 | 🞏 | 🞏 |
| 13. When I dance, I feel better. | 🞏 | 🞏 | 🞏 | 🞏 | 🞏 |
| 14. I find it easy to learn or imitate other people's movements. | 🞏 | 🞏 | 🞏 | 🞏 | 🞏 |
| 15. When I hear a great track, it just makes me want to dance. | 🞏 | 🞏 | 🞏 | 🞏 | 🞏 |

NOTE. Adapted from Müllensiefen, D., Gingras, B., Musil, J., & Stewart, L. (2014). The musicality of non-musicians: An index for assessing musical sophistication in the general population. *PloS one*, *9*(2), e89642. doi:[10.1371/journal.pone.0089642](https://doi.org/10.1371/journal.pone.0089642) and Rose, D., Müllensiefen, D., Lovatt, P., & Orgs, G. (2022). The Goldsmiths Dance Sophistication Index (Gold-DSI): A psychometric tool to assess individual differences in dance experience. *Psychology of Aesthetics, Creativity, and the Arts*, *16*(4), 733. doi:[10.1037/aca0000340](https://doi.org/10.1037/aca0000340). Items were scored on a 5-point scale from 1 (strongly disagree) to 5 (strongly agree), with reverse coding for items 3, 4, 5, 8.

**Abbreviated Brief Music in Mood Regulation scale.**

| Entertainment | Strongly disagree | Somewhat disagree | Neither disagree/agree | Somewhat agree | Strongly agree |
| --- | --- | --- | --- | --- | --- |
| 1. I usually put background music on to make the atmosphere more pleasant. | 🞏 | 🞏 | 🞏 | 🞏 | 🞏 |
| 2. When I’m busy around the house and no one else is around, I like to have some music on the background. | 🞏 | 🞏 | 🞏 | 🞏 | 🞏 |
| Revival |  |  |  |  |  |
| 3. I listen to music to perk up after a rough day. | 🞏 | 🞏 | 🞏 | 🞏 | 🞏 |
| 4. When I’m tired out, I rest by listening to music. | 🞏 | 🞏 | 🞏 | 🞏 | 🞏 |
| Strong sensation |  |  |  |  |  |
| 5. Music has offered me magnificent experiences. | 🞏 | 🞏 | 🞏 | 🞏 | 🞏 |
| 6. I feel fantastic putting my soul fully into the music. | 🞏 | 🞏 | 🞏 | 🞏 | 🞏 |
| Diversion |  |  |  |  |  |
| 7. For me, music is a way to forget about my worries. | 🞏 | 🞏 | 🞏 | 🞏 | 🞏 |
| 8. When stressful thoughts keep going round and round in my head, I start to listen to music to get them off my mind. | 🞏 | 🞏 | 🞏 | 🞏 | 🞏 |
| Discharge |  |  |  |  |  |
| 9. When everything feels bad, it helps me to listen to music that expresses my bad feelings. | 🞏 | 🞏 | 🞏 | 🞏 | 🞏 |
| 10. When I’m really angry, I feel like listening to some aggressive music. | 🞏 | 🞏 | 🞏 | 🞏 | 🞏 |
| Mental work |  |  |  |  |  |
| 11. Music has helped me to get through hard experiences. | 🞏 | 🞏 | 🞏 | 🞏 | 🞏 |
| 12. Music helps me to recognize different feelings in myself. | 🞏 | 🞏 | 🞏 | 🞏 | 🞏 |
| Solace |  |  |  |  |  |
| 13. When I’m feeling sad, listening to music comforts me. | 🞏 | 🞏 | 🞏 | 🞏 | 🞏 |
| 14. I listen to music to find solace when worries overwhelm me. | 🞏 | 🞏 | 🞏 | 🞏 | 🞏 |

NOTE. Adapted from Saarikallio, S. (2012). Brief Music in Mood Regulation Scale. *Music Perception, 30*(1), 97-105. doi:[10.1525/mp.2012.30.1.97](https://doi.org/10.1525/mp.2012.30.1.97). Items were scored on a 5-point scale from 1 (strongly disagree) to 5 (strongly agree).

**TABLE S2.** Leisure activity and rehabilitation for HOME and CARE participants across the study period.

|  | SC | DMI | CMT | *P* |
| --- | --- | --- | --- | --- |
| HOME | | | | |
| Leisure activity (T1-T2) | 2.47 (0.51) | 2.67 (0.42) | 2.70 (0.57) | .365 (*H*) |
| Leisure activity (T2-T3) | 2.58 (0.60) | 2.61 (0.24) | 2.77 (0.54) | .547 (*H*) |
| Rehabilitation (T1-T2) | 0.57 (1.12) | 0.32 (0.56) | 0.71 (1.20) | .517 (*H*) |
| Rehabilitation (T2-T3) | 0.20 (0.41) | 0.46 (0.76) | 0.38 (1.10) | .322 (*H*) |
| CARE | | | | |
| Leisure activity (T1-T2) | 34.85 (28.99) | 36.33 (30.30) | 28.11 (32.33) | .548 (*H*) |
| Leisure activity (T2-T3) | 36.29 (28.87) | 38.76 (33.20) | 37.96 (33.32) | .993 (*H*) |
| Rehabilitation (T1-T2) | 6.58 (8.92) | 4.41 (11.88) | 6.16 (5.50) | .101 (*H*) |
| Rehabilitation (T2-T3) | 5.26 (6.14) | 3.44 (4.15) | 4.90 (4.78) | .713 (*H*) |

NOTE. CARE = persons living with dementia, living in care homes. CMT = conventional music therapy. DMI = digital music intervention. HOME = persons living with dementia, living at home. SC = standard care. Data are mean (SD). Leisure activity for HOME participants was measured on a 5-point scale from 1 (never) to 5 (daily) and averaged across physical exercise, social activity, reading, writing, craft, art, performed arts, music listening, solo singing, group singing, instrument playing, and dancing. Rehabilitation for HOME participants was measured as yes (1) or no (0) and summed across physiotherapy, neuropsychological rehabilitation, occupational therapy, speech therapy, and other therapy. For CARE participants, leisure activity and rehabilitation is measured as hours reported by care home staff.

**TABLE S3.** Primary analyses for secondary outcomes for all, HOME, and CARE participants.

|  |  | *n*, Mean (SD) | | | *P*-value | Interaction *β* [95% CI], *P* | |
| --- | --- | --- | --- | --- | --- | --- | --- |
| Variable | Group | T1 | T2 | T3 | T1 diff. | T1-T2 | T1-T3 |
| All |  |  |  |  |  |  |  |
| Phonemic fluency | SC | 47, 8.81 (5.39) | 43, 8.77 (5.47) | 40, 8.55 (5.25) | .844 |  |  |
|  | DMI | 49, 8.90 (7.14) | 48, 8.67 (6.93) | 46, 8.37 (6.97) |  | -0.10 [-1.61, 1.41], .896 | -0.13 [-1.72, 1.45], .869 |
|  | CMT | 53, 8.19 (5.74) | 50, 8.20 (6.23) | 44, 8.55 (6.39) |  | 0.28 [-1.21, 1.76], .714 | 0.96 [-0.63, 2.55], .234 |
| Categorical fluency | SC | 47, 8.53 (5.64) | 43, 8.77 (6.42) | 40, 8.57 (6.09) | .823 |  |  |
|  | DMI | 49, 9.00 (8.36) | 46, 9.22 (7.80) | 46, 9.59 (8.69) |  | -0.35 [-2.13, 1.43], .696 | 0.49 [-1.48, 2.46], .624 |
|  | CMT | 52, 8.19 (6.15) | 50, 7.98 (5.83) | 44, 7.80 (5.97) |  | -0.43 [-2.17, 1.32], .630 | -0.33 [-2.31, 1.66], .745 |
| CERAD naming | SC | 38, 11.42 (2.88) | 33, 11.21 (3.03) | 30, 11.70 (2.73) | .751 |  |  |
|  | DMI | 36, 10.50 (4.02) | 34, 10.41 (4.31) | 34, 10.47 (4.04) |  | 0.27 [-0.36, 0.90], .398 | 0.08 [-0.69, 0.86], .835 |
|  | CMT | 38, 10.61 (4.04) | 35, 11.09 (4.15) | 31, 10.97 (3.89) |  | 0.69 [0.06, 1.31], .032 | 0.04 [-0.75, 0.84], .911 |
| CERAD copying | SC | 36, 7.67 (2.44) | 31, 7.87 (2.38) | 29, 7.62 (2.69) | .065 |  |  |
|  | DMI | 35, 8.86 (2.26) | 33, 8.79 (2.51) | 33, 8.06 (2.52) |  | -0.10 [-0.88, 0.67], .794 | -0.49 [-1.37, 0.38], .265 |
|  | CMT | 37, 7.97 (2.87) | 35, 7.83 (3.13) | 28, 8.04 (2.67) |  | -0.19 [-0.95, 0.58], .631 | 0.29 [-0.61, 1.20], .523 |
| CERAD WL  immediate recall | SC | 37, 2.68 (1.47) | 33, 2.70 (1.70) | 30, 2.73 (1.36) | .595 |  |  |
|  | DMI | 36, 2.44 (1.76) | 34, 2.82 (1.59) | 34, 2.56 (1.67) |  | 0.28 [-0.44, 1.01], .441 | 0.02 [-0.73, 0.77], .955 |
|  | CMT | 36, 2.94 (1.80) | 34, 2.97 (1.80) | 28, 3.07 (1.86) |  | -0.03 [-0.76, 0.69], .924 | 0.07 [-0.71, 0.85], .855 |
| CERAD WL  learning | SC | 37, 1.51 (1.41) | 33, 1.30 (1.42) | 30, 1.40 (1.71) | .287 |  |  |
|  | DMI | 36, 2.03 (1.36) | 34, 1.74 (1.69) | 34, 1.53 (1.62) |  | -0.05 [-0.83, 0.74], .907 | -0.32 [-1.20, 0.56], .478 |
|  | CMT | 36, 1.61 (1.48) | 34, 1.53 (1.28) | 28, 1.43 (1.50) |  | 0.15 [-0.63, 0.94], .702 | -0.03 [-0.93, 0.88], .954 |
| CERAD WL  delayed recall | SC | 36, 1.58 (1.79) | 31, 1.58 (2.19) | 29, 1.66 (1.90) | .762 |  |  |
|  | DMI | 36, 1.92 (2.26) | 34, 1.82 (2.10) | 34, 2.03 (2.22) |  | -0.09 [-0.96, 0.79], .848 | 0.13 [-0.63, 0.89], .730 |
|  | CMT | 36, 2.19 (2.63) | 34, 2.06 (2.23) | 28, 2.18 (2.31) |  | -0.12 [-1.00, 0.76], .789 | -0.08 [-0.87, 0.72], .845 |
| CERAD WL  recognition | SC | 36, 14.72 (2.78) | 31, 14.55 (3.59) | 29, 14.52 (3.37) | .465 |  |  |
|  | DMI | 36, 15.28 (3.00) | 34, 15.12 (3.10) | 34, 15.65 (3.23) |  | 0.11 [-1.06, 1.29], .848 | 0.71 [-0.67, 2.08], .309 |
|  | CMT | 35, 15.54 (3.15) | 33, 16.09 (2.77) | 28, 15.25 (3.12) |  | 0.75 [-0.43, 1.93], .209 | -0.04 [-1.47, 1.39], .955 |
| WMS-IV LM  immediate recall | SC | 36, 4.17 (2.69) | 31, 4.58 (3.33) | 28, 4.18 (2.50) | .516 |  |  |
|  | DMI | 36, 4.69 (3.13) | 34, 4.71 (2.97) | 34, 4.38 (2.93) |  | -0.29 [-1.67, 1.10], .682 | -0.25 [-1.46, 0.96], .686 |
|  | CMT | 36, 4.97 (2.98) | 34, 4.29 (2.60) | 30, 4.20 (2.50) |  | -1.00 [-2.39, 0.38], .155 | -0.57 [-1.81, 0.67], .360 |
| WMS-IV LM  learning | SC | 36, 2.14 (2.74) | 31, 2.55 (2.47) | 28, 1.50 (2.24) | .461 |  |  |
|  | DMI | 36, 1.58 (2.37) | 34, 2.32 (1.87) | 34, 2.00 (2.24) |  | 0.32 [-1.07, 1.72], .647 | 1.07 [-0.34, 2.48], .136 |
|  | CMT | 36, 1.56 (2.17) | 34, 1.85 (1.81) | 30, 2.47 (2.33) |  | -0.11 [-1.51, 1.28], .871 | 1.57 [0.13, 3.00], .033 |
| WMS-IV LM  delayed recall | SC | 36, 0.89 (1.80) | 31, 1.45 (2.58) | 27, 1.52 (2.78) | .390 |  |  |
|  | DMI | 36, 2.28 (3.24) | 34, 2.47 (3.84) | 34, 2.56 (3.65) |  | -0.37 [-1.44, 0.71], .502 | -0.26 [-1.35, 0.83], .634 |
|  | CMT | 36, 2.03 (3.06) | 34, 2.21 (3.31) | 30, 1.73 (3.06) |  | -0.28 [-1.36, 0.79], .603 | -0.69 [-1.81, 0.43], .222 |
| IFS executive function | SC | 36, 5.12 (3.82) | 31, 5.79 (3.86) | 27, 5.76 (4.18) | .987 |  |  |
|  | DMI | 35, 5.14 (3.96) | 33, 6.17 (4.57) | 33, 5.71 (4.71) |  | 0.42 [-0.84, 1.68], .511 | 0.46 [-1.01, 1.92], .537 |
|  | CMT | 36, 5.07 (3.97) | 34, 5.88 (3.96) | 30, 5.53 (4.66) |  | 0.46 [-0.79, 1.72], .464 | 0.76 [-0.73, 2.26], .314 |
| IFS working memory | SC | 36, 4.58 (2.90) | 31, 4.68 (3.15) | 27, 5.22 (3.26) | .957 |  |  |
|  | DMI | 35, 4.71 (2.92) | 34, 4.59 (2.69) | 34, 4.76 (2.65) |  | -0.21 [-1.05, 0.64], .629 | -0.15 [-0.92, 0.63], .709 |
|  | CMT | 36, 4.61 (2.57) | 34, 4.79 (3.53) | 30, 4.43 (3.16) |  | 0.34 [-0.50, 1.18], .419 | -0.02 [-0.81, 0.77], .963 |
| IFS total | SC | 36, 9.71 (6.21) | 31, 10.47 (6.87) | 27, 10.98 (7.10) | .996 |  |  |
|  | DMI | 35, 9.86 (6.29) | 33, 10.74 (6.86) | 33, 10.47 (6.63) |  | 0.22 [-1.20, 1.64], .758 | 0.38 [-1.34, 2.11], .660 |
|  | CMT | 36, 9.68 (5.98) | 34, 10.68 (6.62) | 30, 9.97 (7.32) |  | 0.87 [-0.54, 2.28], .223 | 0.87 [-0.88, 2.63], .326 |
| Autobiographical  interview | SC | 44, 8.84 (3.18) | 37, 8.76 (3.23) | 35, 9.00 (3.40) | .642 |  |  |
|  | DMI | 48, 8.15 (3.84) | 42, 8.52 (3.34) | 42, 8.45 (3.94) |  | 0.11 [-0.85, 1.08], .815 | 0.04 [-1.05, 1.12], .949 |
|  | CMT | 48, 8.48 (3.33) | 45, 9.36 (3.32) | 39, 8.74 (3.80) |  | 0.87 [-0.09, 1.82], .075 | 0.21 [-0.90, 1.32], .706 |
| B-ART | SC | 51, 4.37 (1.21) | 45, 3.93 (1.31) | 42, 4.05 (1.27) | .115 |  |  |
|  | DMI | 54, 3.99 (1.20) | 52, 3.82 (1.37) | 50, 3.78 (1.41) |  | 0.23 [-0.18, 0.65], .263 | 0.14 [-0.31, 0.59], .538 |
|  | CMT | 56, 4.38 (1.29) | 53, 4.05 (1.37) | 51, 3.86 (1.34) |  | 0.12 [-0.29, 0.53], .572 | -0.16 [-0.60, 0.29], .489 |
| Spontaneous  AM | SC | 51, 23.33 (6.12) | 45, 21.39 (5.53) | 41, 19.70 (4.28) | .620 |  |  |
|  | DMI | 53, 22.97 (5.04) | 53, 21.07 (6.00) | 50, 19.61 (5.32) |  | 0.34 [-1.13, 1.81], .647 | 0.80 [-1.03, 2.63], .389 |
|  | CMT | 57, 22.87 (6.77) | 53, 21.68 (6.31) | 52, 20.09 (5.49) |  | 0.98 [-0.47, 2.44], .184 | 1.39 [-0.42, 3.20], .132 |
| Prompted  AM | SC | 50, 25.28 (6.10) | 43, 22.58 (7.05) | 39, 23.08 (5.89) | .342 |  |  |
|  | DMI | 52, 23.96 (5.35) | 48, 24.00 (6.38) | 46, 22.11 (6.29) |  | 2.85 [0.77, 4.93], .008 | 0.72 [-1.42, 2.87], .505 |
|  | CMT | 56, 24.80 (6.82) | 50, 24.24 (6.60) | 49, 22.43 (6.39) |  | 2.28 [0.23, 4.33], .030 | 0.32 [-1.79, 2.43], .766 |
| MADRS | SC | 49, 12.18 (7.55) | 43, 12.30 (8.29) | 40, 12.57 (7.31) | .986 |  |  |
|  | DMI | 52, 11.83 (6.53) | 49, 13.45 (8.36) | 46, 12.59 (8.10) |  | 0.94 [-1.30, 3.19], .408 | 0.54 [-1.67, 2.76], .629 |
|  | CMT | 56, 11.79 (6.49) | 50, 11.62 (8.06) | 49, 11.67 (8.24) |  | -0.56 [-2.79, 1.66], .617 | -0.39 [-2.57, 1.79], .724 |
| PROMIS Depression | SC | 35, 52.15 (6.24) | 32, 52.69 (6.81) | 31, 51.79 (9.27) | .962 |  |  |
|  | DMI | 35, 52.20 (8.02) | 35, 51.95 (6.98) | 36, 52.55 (7.74) |  | -1.10 [-4.39, 2.19], .509 | 0.81 [-3.00, 4.61], .674 |
|  | CMT | 35, 52.33 (7.77) | 32, 51.61 (8.49) | 31, 52.66 (7.26) |  | -1.23 [-4.56, 2.11], .468 | 0.82 [-3.08, 4.72], .678 |
| PROMIS Anxiety | SC | 35, 48.56 (7.55) | 32, 48.58 (8.56) | 31, 49.64 (8.21) | .464 |  |  |
|  | DMI | 36, 49.34 (9.33) | 35, 49.06 (9.34) | 36, 50.97 (7.46) |  | -0.12 [-4.12, 3.89], .954 | 0.58 [-3.43, 4.59], .774 |
|  | CMT | 34, 51.11 (9.23) | 33, 49.77 (9.49) | 30, 49.78 (9.97) |  | -1.42 [-5.48, 2.64], .489 | -2.66 [-6.80, 1.49], .207 |
| NPI-Q total | SC | 49, 15.84 (17.20) | 43, 16.44 (20.56) | 40, 14.57 (17.80) | .692 |  |  |
|  | DMI | 48, 13.69 (16.08) | 48, 15.50 (17.36) | 44, 13.20 (11.96) |  | -0.46 [-5.81, 4.88], .864 | 0.06 [-5.15, 5.27], .983 |
|  | CMT | 56, 13.16 (15.57) | 49, 12.02 (13.87) | 45, 12.38 (14.31) |  | -2.39 [-7.63, 2.85], .370 | 1.24 [-3.91, 6.38], .635 |
| QOL-AD | SC | 51, 33.91 (6.48) | 45, 33.78 (7.52) | 42, 33.39 (6.92) | .694 |  |  |
|  | DMI | 54, 33.28 (6.01) | 52, 32.38 (6.43) | 48, 32.70 (6.14) |  | -0.24 [-2.04, 1.56], .794 | -0.03 [-1.82, 1.76], .976 |
|  | CMT | 57, 34.15 (6.66) | 53, 34.16 (8.04) | 50, 33.93 (8.45) |  | 0.53 [-1.26, 2.31], .561 | 0.54 [-1.23, 2.31], .547 |
| EQ-5D-5L | SC | 50, 0.79 (0.19) | 45, 0.78 (0.17) | 42, 0.78 (0.18) | .612 |  |  |
|  | DMI | 54, 0.76 (0.20) | 52, 0.77 (0.20) | 48, 0.78 (0.20) |  | 0.03 [-0.03, 0.08], .331 | 0.02 [-0.03, 0.08], .440 |
|  | CMT | 57, 0.80 (0.18) | 52, 0.79 (0.20) | 51, 0.78 (0.21) |  | 0.01 [-0.04, 0.06], .715 | 0.00 [-0.06, 0.06], .981 |
| EQ VAS | SC | 51, 63.21 (20.93) | 45, 65.10 (18.93) | 42, 62.49 (20.33) | .693 |  |  |
|  | DMI | 54, 61.06 (18.55) | 52, 56.97 (22.35) | 46, 61.79 (20.75) |  | -5.16 [-12.48, 2.16], .166 | 1.36 [-5.13, 7.85], .679 |
|  | CMT | 56, 63.26 (19.20) | 53, 62.28 (22.38) | 49, 62.60 (22.04) |  | -2.96 [-10.22, 4.30], .422 | -1.22 [-7.62, 5.18], .707 |
| GHQ | SC | 41, 15.90 (9.14) | 34, 15.47 (8.93) | 33, 15.52 (7.28) | .815 |  |  |
|  | DMI | 47, 14.43 (6.67) | 44, 13.93 (6.25) | 42, 14.00 (7.54) |  | 0.26 [-1.73, 2.24], .799 | 0.08 [-2.11, 2.26], .944 |
|  | CMT | 48, 15.46 (7.67) | 43, 15.33 (7.44) | 42, 14.86 (6.80) |  | 0.55 [-1.44, 2.53], .587 | -0.17 [-2.36, 2.02], .877 |
| ZBI | SC | 41, 17.54 (11.87) | 34, 16.44 (11.78) | 32, 17.03 (12.49) | .715 |  |  |
|  | DMI | 45, 16.71 (10.45) | 44, 15.59 (8.74) | 41, 15.54 (8.67) |  | 0.68 [-2.05, 3.42], .622 | -0.36 [-3.07, 2.36], .796 |
|  | CMT | 48, 15.44 (10.91) | 43, 14.70 (10.78) | 42, 13.90 (10.55) |  | 0.55 [-2.17, 3.28], .688 | -1.32 [-4.02, 1.38], .334 |
| HOME |  |  |  |  |  |  |  |
| Phonemic fluency | SC | 23, 12.09 (4.87) | 21, 11.57 (5.26) | 21, 11.14 (4.90) | .602 |  |  |
|  | DMI | 25, 13.00 (6.85) | 26, 12.00 (7.23) | 26, 11.35 (7.29) |  | 0.31 [-2.23, 2.84], .809 | 0.07 [-2.43, 2.57], .954 |
|  | CMT | 26, 11.54 (5.01) | 25, 11.32 (6.32) | 24, 11.04 (6.66) |  | 0.86 [-1.67, 3.40], .498 | 1.26 [-1.26, 3.78], .321 |
| Categorical fluency | SC | 23, 11.70 (5.60) | 21, 12.14 (7.07) | 21, 11.52 (6.29) | .577 |  |  |
|  | DMI | 25, 13.32 (9.04) | 26, 12.77 (8.23) | 26, 13.46 (9.46) |  | -0.67 [-3.79, 2.45], .669 | 0.66 [-2.61, 3.92], .690 |
|  | CMT | 26, 10.58 (6.87) | 25, 10.48 (6.38) | 24, 9.83 (6.66) |  | -0.55 [-3.67, 2.57], .725 | -0.42 [-3.71, 2.88], .802 |
| CERAD naming | SC | 23, 12.17 (2.92) | 21, 11.95 (2.97) | 21, 11.95 (3.11) | .786 |  |  |
|  | DMI | 25, 11.52 (3.63) | 25, 11.20 (3.95) | 25, 11.28 (3.77) |  | 0.10 [-0.63, 0.82], .789 | 0.17 [-0.76, 1.10], .718 |
|  | CMT | 26, 11.27 (4.04) | 25, 11.36 (4.36) | 23, 11.57 (3.95) |  | 0.50 [-0.23, 1.22], .175 | 0.32 [-0.63, 1.27], .506 |
| CERAD copying | SC | 23, 8.70 (1.61) | 21, 8.38 (2.27) | 21, 8.48 (2.44) | .200 |  |  |
|  | DMI | 25, 9.40 (1.63) | 25, 9.32 (2.25) | 25, 9.00 (1.91) |  | 0.25 [-0.62, 1.12], .568 | -0.17 [-1.12, 0.79], .727 |
|  | CMT | 26, 8.42 (2.69) | 25, 8.04 (2.92) | 21, 8.57 (2.36) |  | 0.03 [-0.84, 0.89], .949 | 0.24 [-0.75, 1.23], .630 |
| CERAD WL  immediate recall | SC | 23, 2.83 (1.50) | 21, 3.29 (1.71) | 21, 2.95 (1.43) | .605 |  |  |
|  | DMI | 25, 2.96 (1.59) | 25, 3.20 (1.53) | 25, 2.96 (1.67) |  | -0.23 [-1.04, 0.58], .573 | -0.14 [-0.92, 0.65], .729 |
|  | CMT | 25, 3.32 (1.57) | 24, 3.21 (1.79) | 21, 3.48 (1.78) |  | -0.59 [-1.41, 0.23], .155 | 0.01 [-0.80, 0.82], .979 |
| CERAD WL  learning | SC | 23, 1.70 (1.22) | 21, 1.48 (1.63) | 21, 1.52 (1.78) | .877 |  |  |
|  | DMI | 25, 1.88 (1.39) | 25, 1.84 (1.72) | 25, 1.68 (1.63) |  | 0.21 [-0.70, 1.11], .645 | -0.01 [-1.09, 1.07], .988 |
|  | CMT | 25, 1.64 (1.60) | 24, 1.62 (1.21) | 21, 1.43 (1.72) |  | 0.22 [-0.69, 1.13], .628 | -0.04 [-1.15, 1.07], .940 |
| CERAD WL  delayed recall | SC | 23, 2.09 (1.90) | 21, 2.14 (2.39) | 21, 2.05 (2.04) | .790 |  |  |
|  | DMI | 25, 2.48 (2.38) | 25, 2.28 (2.23) | 25, 2.56 (2.29) |  | -0.22 [-1.33, 0.89], .697 | 0.16 [-0.80, 1.12], .738 |
|  | CMT | 25, 2.72 (2.67) | 24, 2.38 (2.45) | 21, 2.62 (2.40) |  | -0.36 [-1.48, 0.77], .529 | -0.19 [-1.19, 0.80], .699 |
| CERAD WL  recognition | SC | 23, 14.96 (2.95) | 21, 15.14 (3.44) | 21, 15.29 (2.83) | .170 |  |  |
|  | DMI | 25, 16.08 (2.47) | 25, 15.44 (3.18) | 25, 16.24 (3.10) |  | -0.73 [-2.03, 0.57], .268 | -0.09 [-1.56, 1.39], .907 |
|  | CMT | 24, 16.54 (2.64) | 23, 16.78 (2.35) | 21, 16.05 (2.73) |  | 0.20 [-1.13, 1.52], .770 | -0.82 [-2.35, 0.72], .292 |
| WMS-IV LM  immediate recall | SC | 23, 4.43 (2.74) | 21, 4.90 (3.46) | 20, 4.35 (2.70) | .343 |  |  |
|  | DMI | 25, 5.52 (3.03) | 25, 5.44 (2.77) | 25, 4.96 (2.85) |  | -0.50 [-1.98, 0.99], .507 | -0.44 [-1.93, 1.06], .562 |
|  | CMT | 25, 5.40 (2.74) | 24, 4.29 (2.35) | 22, 4.36 (2.44) |  | -1.51 [-3.00, -0.01], .048 | -0.89 [-2.43, 0.64], .249 |
| WMS-IV LM  learning | SC | 23, 2.35 (3.05) | 21, 2.48 (2.54) | 20, 1.95 (2.04) | .792 |  |  |
|  | DMI | 25, 1.88 (2.55) | 25, 2.40 (2.06) | 25, 2.40 (2.16) |  | 0.42 [-1.33, 2.16], .634 | 0.92 [-0.90, 2.74], .317 |
|  | CMT | 25, 1.96 (1.97) | 24, 1.71 (1.88) | 22, 2.82 (2.50) |  | -0.35 [-2.11, 1.40], .689 | 1.26 [-0.59, 3.11], .180 |
| WMS-IV LM  delayed recall | SC | 23, 1.35 (2.12) | 21, 1.81 (2.93) | 20, 1.90 (3.09) | .658 |  |  |
|  | DMI | 25, 2.76 (3.48) | 25, 2.72 (3.81) | 25, 2.92 (3.87) |  | -0.41 [-1.84, 1.02], .569 | -0.29 [-1.76, 1.19], .698 |
|  | CMT | 25, 2.24 (3.11) | 24, 2.58 (3.50) | 22, 1.86 (2.96) |  | 0.06 [-1.39, 1.50], .939 | -0.78 [-2.29, 0.73], .304 |
| IFS executive function | SC | 23, 6.24 (3.87) | 21, 6.69 (3.96) | 20, 6.78 (4.14) | .686 |  |  |
|  | DMI | 25, 6.26 (3.98) | 25, 7.64 (4.28) | 25, 6.82 (4.83) |  | 1.16 [-0.23, 2.55], .100 | 0.46 [-1.34, 2.25], .613 |
|  | CMT | 25, 5.34 (3.48) | 24, 6.62 (3.90) | 22, 6.30 (4.56) |  | 1.23 [-0.18, 2.63], .085 | 1.17 [-0.67, 3.01], .209 |
| IFS working memory | SC | 23, 5.74 (2.58) | 21, 5.24 (3.19) | 20, 6.05 (3.17) | .659 |  |  |
|  | DMI | 25, 5.64 (2.72) | 25, 5.40 (2.57) | 25, 5.44 (2.63) |  | 0.24 [-0.63, 1.11], .582 | -0.34 [-1.29, 0.61], .483 |
|  | CMT | 25, 5.20 (2.24) | 24, 5.08 (2.84) | 22, 4.95 (3.12) |  | 0.43 [-0.45, 1.30], .333 | -0.29 [-1.27, 0.69], .554 |
| IFS total | SC | 23, 11.98 (6.00) | 21, 11.93 (7.03) | 20, 12.82 (6.97) | .472 |  |  |
|  | DMI | 25, 11.90 (5.99) | 25, 13.04 (6.28) | 25, 12.26 (6.56) |  | 1.42 [-0.19, 3.04], .084 | 0.18 [-1.97, 2.33], .869 |
|  | CMT | 25, 10.54 (5.09) | 24, 11.71 (6.06) | 22, 11.25 (7.08) |  | 1.69 [0.06, 3.32], .042 | 0.98 [-1.23, 3.19], .380 |
| Autobiographical  interview | SC | 23, 10.48 (2.33) | 21, 9.90 (3.30) | 21, 9.90 (3.16) | .992 |  |  |
|  | DMI | 26, 10.04 (3.53) | 26, 9.62 (3.45) | 26, 9.92 (3.84) |  | 0.04 [-1.22, 1.30], .947 | 0.36 [-1.09, 1.80], .623 |
|  | CMT | 25, 10.28 (2.61) | 25, 10.80 (2.97) | 24, 10.54 (3.32) |  | 0.94 [-0.34, 2.22], .148 | 0.70 [-0.78, 2.18], .346 |
| B-ART | SC | 23, 4.71 (0.85) | 21, 4.25 (1.16) | 21, 4.44 (0.92) | 0.14 |  |  |
|  | DMI | 26, 4.24 (1.01) | 25, 4.13 (1.34) | 26, 4.11 (1.39) |  | 0.30 [-0.23, 0.84], .266 | 0.09 [-0.50, 0.69], .756 |
|  | CMT | 26, 4.74 (1.00) | 24, 4.59 (1.07) | 25, 4.41 (0.95) |  | 0.29 [-0.25, 0.83], .286 | -0.10 [-0.70, 0.50], .746 |
| Spontaneous  AM | SC | 23, 25.35 (3.97) | 21, 22.81 (4.22) | 21, 20.69 (3.15) | .088 |  |  |
|  | DMI | 26, 24.33 (4.62) | 25, 23.28 (5.40) | 26, 20.73 (4.84) |  | 1.45 [-0.46, 3.37], .135 | 1.03 [-0.83, 2.90], .272 |
|  | CMT | 26, 26.83 (3.77) | 24, 25.40 (4.13) | 25, 22.56 (3.52) |  | 1.00 [-0.93, 2.93], .306 | 0.29 [-1.59, 2.16], .762 |
| Prompted  AM | SC | 22, 27.36 (3.40) | 20, 24.60 (6.89) | 20, 24.80 (4.75) | .087 |  |  |
|  | DMI | 25, 26.36 (4.04) | 23, 25.91 (6.25) | 24, 24.25 (5.50) |  | 2.37 [-0.62, 5.35], .119 | 0.49 [-1.92, 2.89], .686 |
|  | CMT | 25, 28.52 (5.03) | 23, 27.35 (6.29) | 23, 24.91 (5.61) |  | 1.73 [-1.25, 4.72], .251 | -0.97 [-3.39, 1.46], .428 |
| MADRS | SC | 22, 11.73 (8.58) | 20, 11.85 (9.10) | 20, 11.30 (7.35) | .922 |  |  |
|  | DMI | 25, 11.08 (6.99) | 23, 11.26 (7.37) | 24, 11.54 (8.87) |  | 0.42 [-2.53, 3.36], .778 | 0.90 [-1.86, 3.66], .518 |
|  | CMT | 25, 11.44 (5.93) | 23, 9.96 (6.89) | 23, 10.00 (6.70) |  | -1.85 [-4.80, 1.09], .213 | -1.20 [-3.98, 1.59], .393 |
| PROMIS Depression | SC | 23, 51.98 (5.11) | 21, 53.55 (6.74) | 21, 51.55 (9.30) | .791 |  |  |
|  | DMI | 25, 52.70 (8.22) | 25, 52.03 (6.94) | 26, 51.80 (8.49) |  | -1.94 [-5.82, 1.94], .323 | -0.33 [-4.38, 3.71], .870 |
|  | CMT | 26, 52.15 (7.51) | 24, 51.44 (8.43) | 24, 51.75 (7.69) |  | -1.94 [-5.82, 1.94], .322 | 0.47 [-3.61, 4.55], .819 |
| PROMIS Anxiety | SC | 23, 48.00 (7.88) | 21, 49.11 (8.98) | 21, 50.00 (8.61) | .572 |  |  |
|  | DMI | 25, 49.26 (9.32) | 25, 48.17 (9.38) | 26, 49.26 (7.69) |  | -1.75 [-6.19, 2.68], .433 | -2.13 [-6.54, 2.28], .338 |
|  | CMT | 26, 50.40 (9.57) | 25, 48.30 (9.61) | 24, 48.34 (10.13) |  | -3.11 [-7.51, 1.29], .163 | -4.29 [-8.73, 0.15], .058 |
| NPI-Q total | SC | 22, 17.41 (20.15) | 20, 17.85 (24.56) | 20, 15.05 (19.04) | .837 |  |  |
|  | DMI | 25, 12.84 (17.24) | 23, 10.96 (9.77) | 24, 11.33 (10.45) |  | -4.21 [-11.48, 3.06], .252 | -0.94 [-8.18, 6.30], .796 |
|  | CMT | 25, 12.04 (13.94) | 23, 11.61 (14.64) | 22, 12.27 (13.71) |  | -2.77 [-10.04, 4.50], .450 | 1.85 [-5.52, 9.22], .617 |
| QOL-AD | SC | 23, 37.80 (5.91) | 21, 37.07 (7.73) | 21, 36.38 (6.94) | .774 |  |  |
|  | DMI | 26, 37.00 (5.27) | 25, 36.34 (5.70) | 26, 36.02 (5.47) |  | 0.02 [-2.53, 2.58], .986 | 0.48 [-1.84, 2.81], .679 |
|  | CMT | 26, 37.96 (5.96) | 24, 39.88 (6.68) | 25, 38.62 (7.26) |  | 2.47 [-0.11, 5.04], .060 | 2.00 [-0.35, 4.34], .093 |
| EQ-5D-5L | SC | 23, 0.92 (0.09) | 21, 0.88 (0.16) | 21, 0.90 (0.10) | .318 |  |  |
|  | DMI | 26, 0.90 (0.08) | 25, 0.90 (0.09) | 26, 0.90 (0.09) |  | 0.04 [-0.02, 0.09], .188 | 0.02 [-0.03, 0.07], .393 |
|  | CMT | 26, 0.91 (0.13) | 24, 0.90 (0.16) | 25, 0.91 (0.12) |  | 0.03 [-0.03, 0.08], .336 | 0.02 [-0.03, 0.07], .533 |
| EQ VAS | SC | 23, 72.85 (14.47) | 21, 71.21 (16.03) | 21, 69.26 (16.68) | .478 |  |  |
|  | DMI | 26, 69.69 (14.07) | 25, 68.86 (17.40) | 26, 71.29 (16.42) |  | 0.19 [-8.66, 9.03], .967 | 4.95 [-1.84, 11.75], .151 |
|  | CMT | 26, 73.05 (14.14) | 24, 71.79 (18.89) | 25, 74.02 (15.55) |  | -1.03 [-9.94, 7.88], .819 | 3.39 [-3.46, 10.24], .327 |
| GHQ | SC | 21, 16.38 (8.66) | 19, 14.26 (7.50) | 19, 15.21 (6.76) | .735 |  |  |
|  | DMI | 25, 14.52 (5.10) | 23, 13.35 (4.90) | 24, 13.75 (7.24) |  | 1.26 [-1.60, 4.13], .382 | 0.60 [-2.69, 3.89], .718 |
|  | CMT | 24, 16.08 (6.59) | 23, 15.61 (6.98) | 23, 15.35 (6.44) |  | 1.23 [-1.63, 4.10], .393 | 0.07 [-3.27, 3.41], .968 |
| ZBI | SC | 21, 20.00 (13.68) | 19, 18.11 (12.88) | 18, 21.11 (13.89) | .946 |  |  |
|  | DMI | 23, 17.26 (7.49) | 23, 15.74 (6.83) | 24, 16.67 (8.18) |  | 0.86 [-3.37, 5.08], .686 | -1.38 [-5.48, 2.71], .502 |
|  | CMT | 24, 17.79 (11.06) | 23, 17.52 (10.34) | 23, 18.13 (11.04) |  | 0.92 [-3.27, 5.10], .663 | -1.10 [-5.23, 3.03], .596 |
| CARE |  |  |  |  |  |  |  |
| Phonemic fluency | SC | 24, 5.67 (3.80) | 22, 6.09 (4.26) | 19, 5.68 (4.06) | .426 |  |  |
|  | DMI | 24, 4.62 (4.48) | 22, 4.73 (3.89) | 20, 4.50 (4.16) |  | -0.60 [-2.27, 1.07], .474 | -0.44 [-2.33, 1.45], .640 |
|  | CMT | 27, 4.96 (4.45) | 25, 5.08 (4.36) | 20, 5.55 (4.62) |  | -0.36 [-1.98, 1.26], .659 | 0.52 [-1.36, 2.41], .580 |
| Categorical fluency | SC | 24, 5.50 (3.76) | 22, 5.55 (3.56) | 19, 5.32 (3.87) | .358 |  |  |
|  | DMI | 24, 4.50 (4.36) | 20, 4.60 (3.90) | 20, 4.55 (3.72) |  | -0.25 [-1.97, 1.48], .777 | 0.13 [-1.97, 2.23], .903 |
|  | CMT | 26, 5.81 (4.24) | 25, 5.48 (3.95) | 20, 5.35 (3.92) |  | -0.32 [-1.97, 1.33], .702 | -0.24 [-2.34, 1.85], .816 |
| Autobiographical  interview | SC | 21, 7.05 (3.04) | 16, 7.25 (2.49) | 14, 7.64 (3.39) | .398 |  |  |
|  | DMI | 22, 5.91 (2.93) | 16, 6.75 (2.27) | 16, 6.06 (2.84) |  | 0.23 [-1.33, 1.78], .769 | -0.50 [-2.16, 1.17], .550 |
|  | CMT | 23, 6.52 (2.92) | 20, 7.55 (2.86) | 15, 5.87 (2.56) |  | 0.76 [-0.72, 2.25], .306 | -0.68 [-2.38, 1.03], .427 |
| B-ART | SC | 28, 4.09 (1.38) | 24, 3.65 (1.39) | 21, 3.66 (1.47) | .490 |  |  |
|  | DMI | 28, 3.76 (1.33) | 27, 3.53 (1.35) | 24, 3.42 (1.38) |  | 0.18 [-0.45, 0.81], .570 | 0.21 [-0.45, 0.87], .531 |
|  | CMT | 30, 4.06 (1.44) | 29, 3.61 (1.45) | 26, 3.33 (1.46) |  | -0.01 [-0.63, 0.60], .962 | -0.18 [-0.83, 0.47], .586 |
| Spontaneous  AM | SC | 28, 21.68 (7.08) | 24, 20.15 (6.28) | 20, 18.65 (5.09) | .429 |  |  |
|  | DMI | 27, 21.67 (5.18) | 28, 19.09 (5.90) | 24, 18.40 (5.64) |  | -0.66 [-2.88, 1.55], .553 | 0.56 [-2.63, 3.75], .728 |
|  | CMT | 31, 19.55 (6.98) | 29, 18.60 (6.19) | 27, 17.80 (6.03) |  | 0.99 [-1.18, 3.15], .367 | 2.30 [-0.80, 5.40], .144 |
| Prompted  AM | SC | 28, 23.64 (7.23) | 23, 20.83 (6.87) | 19, 21.26 (6.52) | .320 |  |  |
|  | DMI | 27, 21.74 (5.52) | 25, 22.24 (6.09) | 22, 19.77 (6.38) |  | 3.35 [0.38, 6.31], .028 | 0.95 [-2.66, 4.57], .600 |
|  | CMT | 31, 21.81 (6.65) | 27, 21.59 (5.74) | 26, 20.23 (6.33) |  | 2.73 [-0.16, 5.62], .064 | 1.51 [-1.98, 4.99], .392 |
| MADRS | SC | 27, 12.56 (6.74) | 23, 12.70 (7.71) | 20, 13.85 (7.23) | 0.88 |  |  |
|  | DMI | 27, 12.52 (6.12) | 26, 15.38 (8.84) | 22, 13.73 (7.21) |  | 1.49 [-1.86, 4.84], .379 | 0.20 [-3.26, 3.65], .910 |
|  | CMT | 31, 12.06 (6.99) | 27, 13.04 (8.82) | 26, 13.15 (9.28) |  | 0.53 [-2.76, 3.82], .750 | 0.15 [-3.18, 3.48], .927 |
| NPI-Q total | SC | 27, 14.56 (14.65) | 23, 15.22 (16.83) | 20, 14.10 (16.95) | .802 |  |  |
|  | DMI | 23, 14.61 (15.05) | 25, 19.68 (21.55) | 20, 15.45 (13.48) |  | 3.22 [-4.72, 11.16], .421 | 1.48 [-6.33, 9.29], .706 |
|  | CMT | 31, 14.06 (16.94) | 26, 12.38 (13.43) | 23, 12.48 (15.16) |  | -2.09 [-9.72, 5.54], .586 | 0.70 [-6.75, 8.16], .851 |
| QOL-AD | SC | 28, 30.71 (5.07) | 24, 30.90 (6.14) | 21, 30.40 (5.59) | .490 |  |  |
|  | DMI | 28, 29.82 (4.41) | 27, 28.70 (4.68) | 22, 28.77 (4.34) |  | -0.59 [-3.08, 1.90], .636 | -0.66 [-3.38, 2.07], .633 |
|  | CMT | 31, 30.95 (5.48) | 29, 29.43 (5.68) | 25, 29.24 (6.87) |  | -1.16 [-3.59, 1.27], .345 | -1.02 [-3.67, 1.63], .446 |
| EQ-5D-5L | SC | 27, 0.67 (0.18) | 24, 0.69 (0.13) | 21, 0.66 (0.15) | .452 |  |  |
|  | DMI | 28, 0.64 (0.20) | 27, 0.64 (0.20) | 22, 0.63 (0.18) |  | 0.01 [-0.08, 0.10], .798 | 0.02 [-0.09, 0.12], .771 |
|  | CMT | 31, 0.71 (0.17) | 28, 0.70 (0.19) | 26, 0.67 (0.22) |  | -0.01 [-0.10, 0.08], .807 | -0.02 [-0.12, 0.09], .748 |
| EQ VAS | SC | 28, 55.29 (22.28) | 24, 59.75 (19.96) | 21, 55.71 (21.74) | .939 |  |  |
|  | DMI | 28, 53.05 (18.81) | 27, 45.96 (20.93) | 20, 49.45 (19.52) |  | -10.16 [-21.74, 1.43], .085 | -3.30 [-14.81, 8.21], .569 |
|  | CMT | 30, 54.78 (19.16) | 29, 54.41 (22.26) | 24, 50.71 (21.73) |  | -4.40 [-15.75, 6.95], .443 | -5.70 [-16.77, 5.37], .307 |
| GHQ | SC | 20, 15.40 (9.82) | 15, 17.00 (10.55) | 14, 15.93 (8.19) | .948 |  |  |
|  | DMI | 22, 14.32 (8.23) | 21, 14.57 (7.54) | 18, 14.33 (8.11) |  | -1.18 [-3.84, 1.48], .378 | -0.77 [-3.42, 1.89], .564 |
|  | CMT | 24, 14.83 (8.72) | 20, 15.00 (8.12) | 19, 14.26 (7.35) |  | -0.37 [-3.03, 2.29], .780 | -0.55 [-3.17, 2.07], .674 |
| ZBI | SC | 20, 14.95 (9.28) | 15, 14.33 (10.26) | 14, 11.79 (8.20) | .663 |  |  |
|  | DMI | 22, 16.14 (13.02) | 21, 15.43 (10.62) | 17, 13.94 (9.34) |  | 0.48 [-3.04, 4.01], .785 | 1.10 [-2.17, 4.37], .503 |
|  | CMT | 24, 13.08 (10.45) | 20, 11.45 (10.59) | 19, 8.79 (7.36) |  | 0.20 [-3.32, 3.73], .908 | -1.32 [-4.52, 1.88], .410 |

NOTE. AM = autobiographical memory. B-ART = Brief Autobiographical Recollection Test. CARE = persons living with dementia, living in care homes. CERAD = Consortium to Establish a Registry for Alzheimer’s Disease. CMT = conventional music therapy. DMI = digital music intervention. GHQ = General Health Questionnaire. HOME = persons living with dementia, living at home. IFS = INECO Frontal Screening. LM = logical memory. MADRS = Montgomery-Åsberg Depression Rating Scale. MoCA = Montreal Cognitive Assessment. NPI-Q = Neuropsychiatric Inventory Questionnaire. PROMIS = Patient-Reported Outcomes Measurement Information System. SC = standard care. WL = word list. WMS-IV = Wechsler Memory Scale, Fourth Edition.

**TABLE S4.** Sensitivity analyses for secondary outcomes for all, HOME, and CARE participants.

|  |  | *n*, Mean (SD) | | | *P*-value | Interaction β [95% CI], *P* | |
| --- | --- | --- | --- | --- | --- | --- | --- |
| Variable | Group | T1 | T2 | T3 | T1 diff. | T1-T2 | T1-T3 |
| All |  |  |  |  |  |  |  |
| MoCA total | SC | 39, 13.44 (5.86) | 39, 13.28 (6.04) | 39, 13.31 (6.44) | .894 |  |  |
|  | DMI | 39, 12.92 (6.86) | 39, 13.08 (7.76) | 39, 12.41 (8.04) |  | 0.38 [-0.77, 1.53], .512 | -0.38 [-1.68, 0.91], .557 |
|  | CMT | 38, 13.00 (7.18) | 38, 13.24 (7.33) | 38, 12.42 (7.22) |  | 0.37 [-0.76, 1.50], .519 | -0.45 [-1.75, 0.85], .494 |
| CERAD  Chandler total | SC | 29, 47.97 (14.07) | 29, 47.76 (18.33) | 29, 47.24 (17.45) | .979 |  |  |
|  | DMI | 31, 48.13 (20.76) | 31, 48.26 (21.29) | 31, 46.90 (21.60) |  | 0.58 [-3.68, 4.84], .787 | -0.50 [-4.70, 3.69], .813 |
|  | CMT | 25, 49.32 (18.52) | 25, 49.88 (17.64) | 25, 49.24 (19.34) |  | 1.55 [-2.74, 5.84], .474 | 0.64 [-3.79, 5.08], .773 |
| Phonemic fluency | SC | 40, 9.43 (5.41) | 40, 9.12 (5.34) | 40, 8.55 (5.25) | .444 |  |  |
|  | DMI | 40, 9.95 (7.11) | 40, 9.43 (6.88) | 40, 8.88 (7.09) |  | -0.20 [-1.75, 1.35], .801 | -0.20 [-1.85, 1.45], .810 |
|  | CMT | 41, 8.10 (5.37) | 41, 8.29 (5.64) | 41, 7.95 (5.87) |  | 0.35 [-1.18, 1.87], .654 | 0.73 [-0.91, 2.37], .380 |
| Categorical fluency | SC | 40, 8.85 (5.53) | 40, 9.25 (6.38) | 40, 8.57 (6.09) | .761 |  |  |
|  | DMI | 40, 9.90 (8.34) | 40, 9.62 (7.75) | 40, 10.05 (9.00) |  | -0.52 [-2.34, 1.29], .569 | 0.42 [-1.54, 2.39], .669 |
|  | CMT | 41, 7.80 (5.52) | 41, 8.27 (5.61) | 41, 7.63 (6.07) |  | 0.04 [-1.75, 1.83], .965 | 0.10 [-1.85, 2.06], .916 |
| CERAD naming | SC | 30, 11.97 (2.37) | 30, 11.63 (2.68) | 30, 11.70 (2.73) | .956 |  |  |
|  | DMI | 32, 10.47 (4.17) | 32, 10.22 (4.36) | 32, 10.28 (4.09) |  | 0.23 [-0.40, 0.87], .465 | 0.08 [-0.72, 0.88], .845 |
|  | CMT | 29, 11.21 (3.81) | 29, 11.41 (3.71) | 29, 11.00 (3.85) |  | 0.77 [0.13, 1.40], .019 | 0.06 [-0.76, 0.88], .885 |
| CERAD copying | SC | 29, 7.93 (2.55) | 29, 7.97 (2.43) | 29, 7.62 (2.69) | .180 |  |  |
|  | DMI | 31, 8.71 (2.36) | 31, 8.65 (2.52) | 31, 8.06 (2.56) |  | -0.13 [-0.94, 0.68], .753 | -0.33 [-1.22, 0.55], .454 |
|  | CMT | 26, 7.81 (2.80) | 26, 7.92 (3.12) | 26, 7.96 (2.76) |  | -0.13 [-0.93, 0.68], .755 | 0.46 [-0.46, 1.39], .322 |
| CERAD WL  immediate recall | SC | 30, 2.70 (1.51) | 30, 2.80 (1.71) | 30, 2.73 (1.36) | .522 |  |  |
|  | DMI | 32, 2.53 (1.78) | 32, 2.94 (1.56) | 32, 2.66 (1.68) |  | 0.34 [-0.38, 1.07], .347 | 0.09 [-0.67, 0.86], .812 |
|  | CMT | 26, 2.92 (1.74) | 26, 3.12 (1.84) | 26, 3.12 (1.88) |  | 0.13 [-0.60, 0.86], .721 | 0.16 [-0.65, 0.96], .696 |
| CERAD WL  learning | SC | 30, 1.73 (1.39) | 30, 1.40 (1.45) | 30, 1.40 (1.71) | .387 |  |  |
|  | DMI | 32, 2.06 (1.39) | 32, 1.72 (1.73) | 32, 1.47 (1.65) |  | 0.13 [-0.60, 0.86], .721 | 0.16 [-0.65, 0.96], .696 |
|  | CMT | 26, 1.77 (1.58) | 26, 1.46 (1.14) | 26, 1.31 (1.46) |  | -0.06 [-0.85, 0.73], .875 | -0.26 [-1.17, 0.64], .569 |
| CERAD WL  delayed recall | SC | 29, 1.76 (1.88) | 29, 1.69 (2.22) | 29, 1.66 (1.90) | .947 |  |  |
|  | DMI | 32, 1.97 (2.32) | 32, 1.88 (2.14) | 32, 2.09 (2.26) |  | -0.03 [-0.92, 0.86], .948 | 0.23 [-0.56, 1.02], .566 |
|  | CMT | 26, 2.38 (2.74) | 26, 2.08 (2.28) | 26, 2.15 (2.29) |  | -0.06 [-0.97, 0.84], .887 | -0.13 [-0.96, 0.70], .761 |
| CERAD WL  recognition | SC | 29, 14.76 (2.97) | 29, 14.62 (3.50) | 29, 14.52 (3.37) | .531 |  |  |
|  | DMI | 32, 15.00 (3.03) | 32, 15.12 (3.16) | 32, 15.66 (3.21) |  | 0.25 [-0.97, 1.48], .682 | 0.90 [-0.55, 2.35], .221 |
|  | CMT | 26, 15.65 (3.29) | 26, 16.08 (2.64) | 26, 15.38 (3.05) |  | 0.73 [-0.52, 1.98], .249 | -0.03 [-1.55, 1.50], .971 |
| WMS-IV LM  immediate recall | SC | 28, 4.36 (2.45) | 28, 4.93 (3.31) | 28, 4.18 (2.50) | .694 |  |  |
|  | DMI | 32, 4.69 (3.20) | 32, 4.75 (3.05) | 32, 4.34 (3.01) |  | -0.07 [-1.47, 1.34], .925 | -0.17 [-1.42, 1.09], .794 |
|  | CMT | 28, 4.89 (2.90) | 28, 4.32 (2.51) | 28, 4.14 (2.56) |  | -0.77 [-2.19, 0.64], .281 | -0.57 [-1.87, 0.72], .383 |
| WMS-IV LM  learning | SC | 28, 2.21 (2.75) | 28, 2.61 (2.59) | 28, 1.50 (2.24) | .958 |  |  |
|  | DMI | 32, 1.69 (2.47) | 32, 2.34 (1.93) | 32, 2.06 (2.17) |  | 0.24 [-1.21, 1.68], .746 | 1.09 [-0.42, 2.60], .155 |
|  | CMT | 28, 1.68 (2.36) | 28, 1.89 (1.75) | 28, 2.46 (2.41) |  | -0.19 [-1.65, 1.26], .793 | 1.50 [-0.06, 3.06], .059 |
| WMS-IV LM  delayed recall | SC | 27, 1.11 (2.03) | 27, 1.67 (2.70) | 27, 1.52 (2.78) | .431 |  |  |
|  | DMI | 32, 2.34 (3.32) | 32, 2.50 (3.93) | 32, 2.56 (3.72) |  | -0.30 [-1.40, 0.81], .597 | -0.19 [-1.32, 0.94], .741 |
|  | CMT | 28, 1.82 (3.04) | 28, 2.11 (3.49) | 28, 1.75 (3.15) |  | -0.06 [-1.18, 1.05], .909 | -0.48 [-1.65, 0.69], .417 |
| IFS executive function | SC | 27, 6.07 (3.89) | 27, 6.48 (3.63) | 27, 5.76 (4.18) | .713 |  |  |
|  | DMI | 31, 5.42 (3.98) | 31, 6.08 (4.40) | 31, 5.56 (4.71) |  | 0.34 [-0.94, 1.62], .601 | 0.46 [-1.01, 1.93], .536 |
|  | CMT | 28, 4.71 (3.88) | 28, 5.57 (4.12) | 28, 5.71 (4.74) |  | 0.61 [-0.67, 1.89], .344 | 1.31 [-0.19, 2.82], .087 |
| IFS working memory | SC | 27, 5.30 (2.73) | 27, 5.33 (2.81) | 27, 5.22 (3.26) | .592 |  |  |
|  | DMI | 31, 4.97 (2.90) | 31, 4.61 (2.76) | 31, 4.84 (2.73) |  | -0.23 [-1.10, 0.65], .610 | -0.05 [-0.86, 0.75], .892 |
|  | CMT | 28, 4.43 (2.52) | 28, 4.54 (3.64) | 28, 4.39 (3.08) |  | 0.35 [-0.52, 1.23], .424 | 0.04 [-0.79, 0.86], .927 |
| IFS total | SC | 27, 11.37 (6.09) | 27, 11.81 (6.30) | 27, 10.98 (7.10) | .641 |  |  |
|  | DMI | 31, 10.39 (6.24) | 31, 10.69 (6.73) | 31, 10.40 (6.69) |  | 0.11 [-1.33, 1.55], .877 | 0.41 [-1.35, 2.16], .648 |
|  | CMT | 28, 9.14 (5.75) | 28, 10.11 (6.77) | 28, 10.11 (7.39) |  | 0.97 [-0.47, 2.41], .186 | 1.35 [-0.45, 3.16], .139 |
| Autobiographical  interview | SC | 34, 9.26 (2.81) | 34, 8.85 (3.35) | 34, 8.97 (3.44) | .778 |  |  |
|  | DMI | 37, 9.03 (3.53) | 37, 8.73 (3.44) | 37, 8.62 (3.97) |  | 0.05 [-0.96, 1.06], .917 | -0.10 [-1.22, 1.02], .858 |
|  | CMT | 33, 8.91 (3.00) | 33, 9.64 (3.01) | 33, 8.97 (3.83) |  | 0.92 [-0.09, 1.93], .073 | 0.50 [-0.65, 1.65], .389 |
| B-ART | SC | 40, 4.52 (1.00) | 40, 4.01 (1.30) | 40, 4.09 (1.27) | .326 |  |  |
|  | DMI | 44, 4.09 (1.24) | 44, 3.84 (1.37) | 44, 3.89 (1.44) |  | 0.19 [-0.20, 0.58], .338 | 0.23 [-0.24, 0.70], .343 |
|  | CMT | 43, 4.31 (1.25) | 43, 4.20 (1.31) | 43, 3.88 (1.34) |  | 0.31 [-0.08, 0.70], .121 | -0.04 [-0.51, 0.43], .857 |
| Spontaneous  AM | SC | 39, 24.82 (4.82) | 39, 22.44 (4.49) | 39, 20.01 (3.87) | .463 |  |  |
|  | DMI | 44, 23.41 (4.91) | 44, 21.62 (5.97) | 44, 19.82 (5.32) |  | 0.47 [-1.00, 1.94], .529 | 1.06 [-0.87, 2.98], .278 |
|  | CMT | 45, 23.06 (6.52) | 45, 22.19 (6.25) | 45, 20.31 (5.26) |  | 1.45 [-0.02, 2.91], .053 | 1.85 [-0.06, 3.75], .057 |
| Prompted  AM | SC | 37, 26.62 (4.38) | 37, 23.22 (6.74) | 37, 23.46 (5.78) | .471 |  |  |
|  | DMI | 41, 24.68 (5.15) | 41, 24.37 (6.56) | 41, 22.46 (6.32) |  | 2.71 [0.55, 4.87], .014 | 0.96 [-1.29, 3.22], .399 |
|  | CMT | 42, 24.50 (6.62) | 42, 24.29 (6.75) | 42, 22.10 (6.14) |  | 2.77 [0.60, 4.94], .013 | 0.67 [-1.56, 2.91], .552 |
| MADRS | SC | 38, 11.89 (7.70) | 38, 12.89 (8.49) | 38, 12.58 (7.43) | .945 |  |  |
|  | DMI | 41, 10.90 (6.74) | 41, 12.37 (7.47) | 41, 11.78 (7.36) |  | 0.51 [-1.76, 2.78], .658 | 0.21 [-2.01, 2.44], .851 |
|  | CMT | 42, 10.90 (6.54) | 42, 11.21 (7.83) | 42, 11.05 (7.89) |  | -0.60 [-2.88, 1.68], .603 | -0.64 [-2.84, 1.56], .564 |
| PROMIS Depression | SC | 29, 51.96 (6.40) | 29, 53.22 (6.81) | 29, 51.52 (9.53) | .937 |  |  |
|  | DMI | 31, 51.94 (8.05) | 31, 51.67 (7.10) | 31, 51.75 (7.89) |  | -1.02 [-4.42, 2.39], .554 | 1.05 [-2.92, 5.03], .600 |
|  | CMT | 26, 51.24 (7.90) | 26, 51.90 (8.60) | 26, 51.91 (7.69) |  | -0.92 [-4.38, 2.55], .600 | 1.46 [-2.65, 5.57], .483 |
| PROMIS Anxiety | SC | 29, 48.14 (7.88) | 29, 48.97 (8.88) | 29, 49.61 (8.50) | .331 |  |  |
|  | DMI | 32, 48.34 (9.05) | 32, 49.30 (9.43) | 32, 50.93 (7.88) |  | 0.76 [-3.26, 4.78], .707 | 1.33 [-2.70, 5.36], .513 |
|  | CMT | 26, 50.30 (8.83) | 26, 49.71 (9.86) | 26, 48.97 (10.10) |  | -1.18 [-5.26, 2.91], .569 | -2.46 [-6.66, 1.74], .247 |
| NPI-Q total | SC | 38, 15.00 (15.69) | 38, 17.08 (21.54) | 38, 15.16 (18.07) | .488 |  |  |
|  | DMI | 39, 13.49 (16.85) | 39, 15.08 (17.11) | 39, 12.62 (11.47) |  | -1.10 [-6.67, 4.48], .698 | -0.87 [-6.30, 4.55], .751 |
|  | CMT | 37, 10.92 (12.58) | 37, 11.19 (13.28) | 37, 12.62 (13.56) |  | -1.90 [-7.48, 3.67], .500 | 1.38 [-4.04, 6.81], .614 |
| QOL-AD | SC | 40, 34.85 (6.57) | 40, 34.02 (7.92) | 40, 33.45 (7.09) | .383 |  |  |
|  | DMI | 43, 34.50 (5.91) | 43, 33.40 (6.22) | 43, 33.20 (5.99) |  | -0.16 [-1.98, 1.65], .858 | -0.06 [-1.82, 1.70], .948 |
|  | CMT | 43, 35.07 (6.93) | 43, 35.15 (7.97) | 43, 34.93 (8.32) |  | 0.88 [-0.94, 2.71], .341 | 1.02 [-0.74, 2.77], .253 |
| EQ-5D-5L | SC | 40, 0.83 (0.15) | 40, 0.80 (0.17) | 40, 0.80 (0.17) | .234 |  |  |
|  | DMI | 43, 0.80 (0.18) | 43, 0.79 (0.20) | 43, 0.80 (0.17) |  | 0.02 [-0.03, 0.07], .342 | 0.03 [-0.02, 0.08], .216 |
|  | CMT | 44, 0.82 (0.18) | 44, 0.81 (0.20) | 44, 0.80 (0.21) |  | 0.01 [-0.04, 0.06], .567 | 0.01 [-0.04, 0.06], .660 |
| EQ VAS | SC | 40, 64.46 (20.93) | 40, 65.05 (19.95) | 40, 63.30 (20.48) | .355 |  |  |
|  | DMI | 42, 63.57 (17.60) | 42, 59.18 (21.75) | 42, 62.86 (20.47) |  | -5.18 [-12.64, 2.28], .172 | 0.60 [-5.87, 7.08], .854 |
|  | CMT | 42, 68.13 (15.90) | 42, 64.42 (20.66) | 42, 65.55 (20.11) |  | -3.17 [-10.67, 4.32], .404 | -0.81 [-7.25, 5.63], .804 |
| GHQ | SC | 31, 15.35 (9.13) | 31, 14.55 (7.95) | 31, 15.29 (7.10) | .882 |  |  |
|  | DMI | 37, 14.00 (5.95) | 37, 13.41 (5.93) | 37, 13.54 (7.18) |  | 0.03 [-1.95, 2.02], .974 | -0.46 [-2.64, 1.72], .676 |
|  | CMT | 36, 14.58 (7.38) | 36, 15.31 (7.70) | 36, 14.50 (7.02) |  | 0.83 [-1.16, 2.83], .409 | -0.08 [-2.28, 2.11], .940 |
| ZBI | SC | 30, 17.17 (11.75) | 30, 15.97 (11.62) | 30, 17.50 (12.56) | .438 |  |  |
|  | DMI | 36, 15.92 (8.96) | 36, 15.14 (8.19) | 36, 15.47 (8.13) |  | 0.39 [-2.32, 3.11], .774 | -0.73 [-3.59, 2.12], .611 |
|  | CMT | 36, 14.67 (11.45) | 36, 14.53 (11.09) | 36, 13.61 (10.86) |  | 0.82 [-1.90, 3.53], .552 | -1.35 [-4.20, 1.51], .353 |
| HOME |  |  |  |  |  |  |  |
| MoCA total | SC | 21, 16.05 (5.51) | 21, 15.29 (6.37) | 21, 16.19 (6.53) | .930 |  |  |
|  | DMI | 25, 15.84 (6.50) | 25, 16.24 (7.73) | 25, 15.80 (7.69) |  | 1.16 [-0.65, 2.98], .206 | -0.18 [-2.05, 1.69], .846 |
|  | CMT | 22, 15.91 (6.37) | 22, 16.18 (6.61) | 22, 15.36 (6.61) |  | 1.01 [-0.82, 2.84], .274 | -0.69 [-2.61, 1.24], .478 |
| CERAD  Chandler total | SC | 21, 51.52 (14.64) | 21, 52.10 (19.40) | 21, 51.10 (18.50) | .798 |  |  |
|  | DMI | 24, 54.08 (19.13) | 24, 54.12 (20.00) | 24, 53.38 (19.75) |  | -0.53 [-5.83, 4.78], .843 | -0.28 [-5.30, 4.75], .912 |
|  | CMT | 19, 54.42 (16.53) | 19, 53.68 (16.54) | 19, 54.00 (17.44) |  | -1.71 [-7.12, 3.71], .531 | 0.01 [-5.32, 5.33], .998 |
| Phonemic fluency | SC | 21, 12.67 (4.65) | 21, 11.57 (5.26) | 21, 11.14 (4.90) | .291 |  |  |
|  | DMI | 24, 12.88 (6.97) | 24, 12.25 (6.99) | 24, 11.71 (7.19) |  | 0.47 [-2.05, 2.99], .711 | 0.36 [-2.15, 2.87], .777 |
|  | CMT | 23, 10.74 (4.75) | 23, 10.61 (5.76) | 23, 10.43 (6.10) |  | 0.72 [-1.80, 3.24], .571 | 1.22 [-1.32, 3.76], .341 |
| Categorical fluency | SC | 21, 11.67 (5.43) | 21, 12.14 (7.07) | 21, 11.52 (6.29) | .321 |  |  |
|  | DMI | 24, 12.92 (9.00) | 24, 12.88 (8.00) | 24, 13.88 (9.49) |  | -0.52 [-3.69, 2.65], .745 | 1.10 [-2.00, 4.20], .481 |
|  | CMT | 23, 9.61 (6.25) | 23, 10.09 (6.33) | 23, 9.78 (6.80) |  | -0.27 [-3.44, 2.90], .866 | 0.32 [-2.82, 3.45], .841 |
| CERAD naming | SC | 21, 12.38 (2.52) | 21, 11.95 (2.97) | 21, 11.95 (3.11) | .738 |  |  |
|  | DMI | 24, 11.46 (3.69) | 24, 11.17 (4.03) | 24, 11.17 (3.81) |  | 0.14 [-0.60, 0.88], .714 | 0.14 [-0.81, 1.09], .775 |
|  | CMT | 22, 11.55 (3.81) | 22, 11.59 (3.81) | 22, 11.41 (3.97) |  | 0.51 [-0.23, 1.25], .173 | 0.29 [-0.68, 1.26], .550 |
| CERAD copying | SC | 21, 8.71 (1.68) | 21, 8.38 (2.27) | 21, 8.48 (2.44) | .227 |  |  |
|  | DMI | 24, 9.38 (1.66) | 24, 9.25 (2.27) | 24, 8.96 (1.94) |  | 0.21 [-0.68, 1.10], .641 | -0.18 [-1.15, 0.79], .714 |
|  | CMT | 20, 8.55 (2.42) | 20, 8.50 (2.61) | 20, 8.55 (2.42) |  | 0.08 [-0.80, 0.97], .852 | 0.24 [-0.78, 1.25], .641 |
| CERAD WL  immediate recall | SC | 21, 2.81 (1.54) | 21, 3.29 (1.71) | 21, 2.95 (1.43) | .759 |  |  |
|  | DMI | 24, 3.04 (1.57) | 24, 3.29 (1.49) | 24, 3.04 (1.65) |  | -0.23 [-1.06, 0.61], .591 | -0.14 [-0.95, 0.67], .725 |
|  | CMT | 20, 3.25 (1.68) | 20, 3.40 (1.79) | 20, 3.45 (1.82) |  | -0.56 [-1.41, 0.28], .188 | 0.06 [-0.79, 0.90], .893 |
| CERAD WL  learning | SC | 21, 1.76 (1.22) | 21, 1.48 (1.63) | 21, 1.52 (1.78) | .528 |  |  |
|  | DMI | 24, 1.96 (1.37) | 24, 1.88 (1.75) | 24, 1.67 (1.66) |  | 0.20 [-0.70, 1.11], .657 | -0.05 [-1.14, 1.03], .922 |
|  | CMT | 20, 1.75 (1.71) | 20, 1.50 (1.10) | 20, 1.30 (1.66) |  | 0.11 [-0.80, 1.03], .808 | -0.21 [-1.34, 0.92], .710 |
| CERAD WL  delayed recall | SC | 21, 2.14 (1.98) | 21, 2.14 (2.39) | 21, 2.05 (2.04) | .942 |  |  |
|  | DMI | 24, 2.58 (2.38) | 24, 2.38 (2.22) | 24, 2.67 (2.28) |  | -0.21 [-1.34, 0.93], .715 | 0.18 [-0.81, 1.16], .718 |
|  | CMT | 20, 2.90 (2.88) | 20, 2.45 (2.44) | 20, 2.50 (2.40) |  | -0.43 [-1.58, 0.71], .452 | -0.30 [-1.33, 0.72], .556 |
| CERAD WL  recognition | SC | 21, 15.10 (3.05) | 21, 15.14 (3.44) | 21, 15.29 (2.83) | .668 |  |  |
|  | DMI | 24, 16.12 (2.51) | 24, 15.54 (3.20) | 24, 16.42 (3.03) |  | -0.63 [-1.97, 0.71], .351 | 0.10 [-1.41, 1.61], .894 |
|  | CMT | 20, 16.60 (2.78) | 20, 16.50 (2.35) | 20, 16.00 (2.79) |  | 0.23 [-1.14, 1.59], .744 | -0.79 [-2.37, 0.79], .321 |
| WMS-IV LM  immediate recall | SC | 20, 4.50 (2.70) | 20, 5.05 (3.49) | 20, 4.35 (2.70) | .876 |  |  |
|  | DMI | 24, 5.58 (3.08) | 24, 5.46 (2.83) | 24, 5.00 (2.90) |  | -0.51 [-2.02, 1.01], .508 | -0.43 [-1.98, 1.11], .577 |
|  | CMT | 21, 5.38 (2.96) | 21, 4.43 (2.38) | 21, 4.29 (2.47) |  | -1.55 [-3.09, -0.02], .047 | -0.95 [-2.54, 0.65], .241 |
| WMS-IV LM  learning | SC | 20, 2.35 (3.13) | 20, 2.50 (2.61) | 20, 1.95 (2.04) | .744 |  |  |
|  | DMI | 24, 1.88 (2.61) | 24, 2.42 (2.10) | 24, 2.33 (2.18) |  | 0.54 [-1.25, 2.33], .547 | 0.86 [-1.05, 2.77], .372 |
|  | CMT | 21, 1.95 (2.16) | 21, 1.76 (1.76) | 21, 2.81 (2.56) |  | -0.09 [-1.89, 1.72], .924 | 1.26 [-0.71, 3.23], .207 |
| WMS-IV LM  delayed recall | SC | 20, 1.50 (2.24) | 20, 1.90 (2.97) | 20, 1.90 (3.09) | .322 |  |  |
|  | DMI | 24, 2.88 (3.51) | 24, 2.83 (3.85) | 24, 3.04 (3.91) |  | -0.38 [-1.81, 1.06], .604 | -0.23 [-1.73, 1.27], .757 |
|  | CMT | 21, 1.95 (3.12) | 21, 2.48 (3.71) | 21, 1.81 (3.03) |  | 0.32 [-1.13, 1.77], .662 | -0.54 [-2.09, 1.00], .486 |
| IFS executive function | SC | 20, 6.83 (3.71) | 20, 6.97 (3.83) | 20, 6.78 (4.14) | .397 |  |  |
|  | DMI | 24, 6.19 (4.05) | 24, 7.38 (4.16) | 24, 6.60 (4.81) |  | 1.02 [-0.34, 2.38], .138 | 0.47 [-1.27, 2.20], .593 |
|  | CMT | 21, 4.62 (3.28) | 21, 6.33 (4.08) | 21, 6.36 (4.67) |  | 1.46 [0.09, 2.84], .037 | 1.79 [-0.01, 3.58], .051 |
| IFS working memory | SC | 20, 5.95 (2.54) | 20, 5.50 (3.03) | 20, 6.05 (3.17) | .301 |  |  |
|  | DMI | 24, 5.62 (2.78) | 24, 5.38 (2.62) | 24, 5.46 (2.69) |  | 0.23 [-0.65, 1.10], .608 | -0.27 [-1.24, 0.71], .586 |
|  | CMT | 21, 4.95 (2.27) | 21, 4.76 (2.79) | 21, 4.76 (3.06) |  | 0.35 [-0.54, 1.23], .438 | -0.29 [-1.29, 0.71], .565 |
| IFS total | SC | 20, 12.78 (5.83) | 20, 12.47 (6.74) | 20, 12.82 (6.97) | .239 |  |  |
|  | DMI | 24, 11.81 (6.11) | 24, 12.75 (6.24) | 24, 12.06 (6.63) |  | 1.25 [-0.37, 2.86], .128 | 0.20 [-1.95, 2.35], .853 |
|  | CMT | 21, 9.57 (4.85) | 21, 11.10 (6.17) | 21, 11.12 (7.23) |  | 1.81 [0.18, 3.44], .030 | 1.50 [-0.72, 3.72], .182 |
| Autobiographical  interview | SC | 21, 10.33 (2.37) | 21, 9.90 (3.30) | 21, 9.90 (3.16) | .904 |  |  |
|  | DMI | 25, 10.00 (3.59) | 25, 9.60 (3.52) | 25, 9.92 (3.91) |  | 0.03 [-1.27, 1.33], .965 | 0.35 [-1.14, 1.83], .641 |
|  | CMT | 22, 10.14 (2.66) | 22, 10.59 (3.02) | 22, 10.45 (3.46) |  | 0.86 [-0.46, 2.19], .198 | 0.75 [-0.78, 2.28], .333 |
| B-ART | SC | 21, 4.62 (0.82) | 21, 4.25 (1.16) | 21, 4.44 (0.92) | .174 |  |  |
|  | DMI | 25, 4.22 (1.03) | 25, 4.13 (1.34) | 25, 4.12 (1.41) |  | 0.27 [-0.27, 0.82], .318 | 0.07 [-0.54, 0.68], .818 |
|  | CMT | 23, 4.65 (1.03) | 23, 4.56 (1.08) | 23, 4.43 (0.99) |  | 0.28 [-0.27, 0.84], .310 | -0.11 [-0.72, 0.51], .734 |
| Spontaneous  AM | SC | 21, 25.31 (4.16) | 21, 22.81 (4.22) | 21, 20.69 (3.15) | .053 |  |  |
|  | DMI | 25, 24.34 (4.71) | 25, 23.28 (5.40) | 25, 20.82 (4.92) |  | 1.44 [-0.51, 3.39], .144 | 1.10 [-0.81, 3.01], .254 |
|  | CMT | 23, 26.74 (3.84) | 23, 25.28 (4.18) | 23, 22.52 (3.64) |  | 1.04 [-0.94, 3.03], .298 | 0.35 [-1.58, 2.27], .719 |
| Prompted  AM | SC | 20, 27.35 (3.54) | 20, 24.60 (6.89) | 20, 24.80 (4.75) | .049 |  |  |
|  | DMI | 23, 26.26 (4.20) | 23, 25.91 (6.25) | 23, 24.48 (5.51) |  | 2.40 [-0.66, 5.47], .122 | 0.77 [-1.66, 3.20], .530 |
|  | CMT | 21, 27.95 (5.20) | 21, 27.10 (6.53) | 21, 24.43 (5.60) |  | 1.84 [-1.26, 4.94], .239 | -1.13 [-3.59, 1.32], .360 |
| MADRS | SC | 20, 11.45 (8.96) | 20, 11.85 (9.10) | 20, 11.30 (7.35) | .570 |  |  |
|  | DMI | 23, 10.35 (6.79) | 23, 11.26 (7.37) | 23, 10.48 (7.34) |  | 0.51 [-2.47, 3.50], .732 | 0.28 [-2.33, 2.89], .831 |
|  | CMT | 21, 11.43 (6.49) | 21, 10.14 (7.19) | 21, 10.10 (6.96) |  | -1.81 [-4.82, 1.21], .235 | -1.35 [-3.99, 1.29], .310 |
| PROMIS Depression | SC | 21, 52.19 (5.19) | 21, 53.55 (6.74) | 21, 51.55 (9.30) | .763 |  |  |
|  | DMI | 24, 52.25 (8.07) | 24, 51.90 (7.05) | 24, 51.64 (8.80) |  | -1.72 [-5.68, 2.24], .388 | 0.03 [-4.14, 4.19], .990 |
|  | CMT | 22, 51.18 (7.76) | 22, 51.21 (8.61) | 22, 51.19 (7.78) |  | -1.76 [-5.76, 2.24], .383 | 0.72 [-3.49, 4.93], .734 |
| PROMIS Anxiety | SC | 21, 47.88 (8.03) | 21, 49.11 (8.98) | 21, 50.00 (8.61) | .511 |  |  |
|  | DMI | 24, 48.54 (8.78) | 24, 48.27 (9.57) | 24, 48.94 (7.90) |  | -1.51 [-6.01, 2.99], .504 | -1.73 [-6.18, 2.71], .439 |
|  | CMT | 22, 49.82 (9.44) | 22, 48.19 (9.67) | 22, 47.19 (9.69) |  | -3.05 [-7.55, 1.45], .181 | -4.69 [-9.19, -0.20], .041 |
| NPI-Q total | SC | 20, 15.35 (17.46) | 20, 17.85 (24.56) | 20, 15.05 (19.04) | .901 |  |  |
|  | DMI | 23, 13.26 (17.88) | 23, 10.96 (9.77) | 23, 10.22 (9.11) |  | -4.80 [-12.14, 2.54], .196 | -2.74 [-9.84, 4.35], .442 |
|  | CMT | 20, 11.20 (14.13) | 20, 11.15 (15.17) | 20, 12.75 (14.25) |  | -3.23 [-10.64, 4.19], .388 | 1.35 [-5.90, 8.60], .711 |
| QOL-AD | SC | 21, 37.86 (6.08) | 21, 37.07 (7.73) | 21, 36.38 (6.94) | .763 |  |  |
|  | DMI | 25, 37.12 (5.34) | 25, 36.34 (5.70) | 25, 36.18 (5.52) |  | 0.01 [-2.59, 2.60], .997 | 0.54 [-1.83, 2.90], .653 |
|  | CMT | 23, 38.20 (6.25) | 23, 39.78 (6.82) | 23, 39.09 (7.36) |  | 2.37 [-0.27, 5.02], .078 | 2.16 [-0.23, 4.55], .075 |
| EQ-5D-5L | SC | 21, 0.92 (0.09) | 21, 0.88 (0.16) | 21, 0.90 (0.10) | .393 |  |  |
|  | DMI | 25, 0.90 (0.08) | 25, 0.90 (0.09) | 25, 0.91 (0.09) |  | 0.03 [-0.02, 0.09], .217 | 0.02 [-0.03, 0.07], .348 |
|  | CMT | 23, 0.91 (0.14) | 23, 0.89 (0.16) | 23, 0.90 (0.12) |  | 0.02 [-0.03, 0.08], .379 | 0.01 [-0.04, 0.06], .590 |
| EQ VAS | SC | 21, 72.52 (15.08) | 21, 71.21 (16.03) | 21, 69.26 (16.68) | .341 |  |  |
|  | DMI | 25, 70.48 (13.76) | 25, 68.86 (17.40) | 25, 71.24 (16.76) |  | -0.31 [-9.22, 8.59], .945 | 4.02 [-2.70, 10.75], .237 |
|  | CMT | 23, 75.22 (12.40) | 23, 71.33 (19.18) | 23, 74.70 (16.02) |  | -2.58 [-11.66, 6.50], .572 | 3.47 [-3.32, 10.26], .311 |
| GHQ | SC | 19, 16.11 (8.99) | 19, 14.26 (7.50) | 19, 15.21 (6.76) | .418 |  |  |
|  | DMI | 23, 13.83 (4.40) | 23, 13.35 (4.90) | 23, 12.96 (6.25) |  | 1.36 [-1.36, 4.09], .321 | 0.03 [-3.10, 3.15], .987 |
|  | CMT | 21, 16.10 (6.85) | 21, 16.29 (6.78) | 21, 15.33 (6.61) |  | 1.66 [-1.09, 4.41], .233 | 0.13 [-3.06, 3.33], .934 |
| ZBI | SC | 18, 19.67 (13.09) | 18, 18.44 (13.17) | 18, 21.11 (13.89) | .856 |  |  |
|  | DMI | 22, 16.45 (6.57) | 22, 16.18 (6.64) | 22, 16.64 (6.76) |  | 0.99 [-3.18, 5.16], .636 | -1.26 [-5.48, 2.96], .552 |
|  | CMT | 21, 17.43 (11.59) | 21, 17.71 (10.80) | 21, 17.67 (11.45) |  | 1.22 [-2.95, 5.39], .561 | -1.21 [-5.47, 3.06], .573 |
| CARE |  |  |  |  |  |  |  |
| MoCA total | SC | 18, 10.39 (4.78) | 18, 10.94 (4.82) | 18, 9.94 (4.50) | .673 |  |  |
|  | DMI | 14, 7.71 (3.75) | 14, 7.43 (3.46) | 14, 6.36 (4.27) |  | -0.50 [-1.85, 0.85], .462 | -0.91 [-2.67, 0.84], .301 |
|  | CMT | 16, 9.00 (6.41) | 16, 9.19 (6.42) | 16, 8.38 (6.10) |  | -0.29 [-1.56, 0.99], .655 | -0.18 [-1.87, 1.51], .831 |
| Phonemic fluency | SC | 19, 5.84 (3.69) | 19, 6.42 (4.03) | 19, 5.68 (4.06) | .683 |  |  |
|  | DMI | 16, 5.56 (4.77) | 16, 5.19 (4.00) | 16, 4.62 (4.38) |  | -0.79 [-2.55, 0.97], .373 | -0.78 [-2.85, 1.29], .453 |
|  | CMT | 18, 4.72 (4.16) | 18, 5.33 (3.93) | 18, 4.78 (3.73) |  | 0.07 [-1.62, 1.76], .935 | 0.21 [-1.80, 2.22], .832 |
| Categorical fluency | SC | 19, 5.74 (3.74) | 19, 6.05 (3.47) | 19, 5.32 (3.87) | .406 |  |  |
|  | DMI | 16, 5.38 (4.50) | 16, 4.75 (3.97) | 16, 4.31 (3.74) |  | -0.63 [-2.25, 0.98], .436 | -0.64 [-2.83, 1.55], .559 |
|  | CMT | 18, 5.50 (3.35) | 18, 5.94 (3.46) | 18, 4.89 (3.55) |  | 0.35 [-1.19, 1.90], .648 | -0.19 [-2.31, 1.93], .858 |
| Autobiographical  interview | SC | 13, 7.54 (2.67) | 13, 7.15 (2.76) | 13, 7.46 (3.45) | .566 |  |  |
|  | DMI | 12, 7.00 (2.45) | 12, 6.92 (2.50) | 12, 5.92 (2.54) |  | 0.14 [-1.58, 1.86], .872 | -0.92 [-2.66, 0.82], .289 |
|  | CMT | 11, 6.45 (2.02) | 11, 7.73 (1.95) | 11, 6.00 (2.68) |  | 1.01 [-0.64, 2.67], .223 | 0.08 [-1.70, 1.85], .930 |
| B-ART | SC | 19, 4.40 (1.19) | 19, 3.76 (1.42) | 19, 3.71 (1.51) | .847 |  |  |
|  | DMI | 19, 3.91 (1.48) | 19, 3.47 (1.37) | 19, 3.60 (1.45) |  | 0.08 [-0.50, 0.65], .788 | 0.37 [-0.37, 1.11], .325 |
|  | CMT | 20, 3.92 (1.38) | 20, 3.79 (1.46) | 20, 3.25 (1.43) |  | 0.33 [-0.25, 0.90], .259 | -0.00 [-0.73, 0.73], .992 |
| Spontaneous  AM | SC | 18, 24.25 (5.57) | 18, 22.00 (4.87) | 18, 19.22 (4.53) | .367 |  |  |
|  | DMI | 19, 22.18 (5.01) | 19, 19.45 (6.11) | 19, 18.50 (5.67) |  | -0.63 [-2.86, 1.61], .577 | 1.00 [-2.56, 4.56], .576 |
|  | CMT | 22, 19.20 (6.57) | 22, 18.95 (6.49) | 22, 18.00 (5.75) |  | 1.87 [-0.31, 4.05], .092 | 3.48 [0.04, 6.92], .047 |
| Prompted  AM | SC | 17, 25.76 (5.18) | 17, 21.59 (6.37) | 17, 21.88 (6.59) | .793 |  |  |
|  | DMI | 18, 22.67 (5.66) | 18, 22.39 (6.59) | 18, 19.89 (6.50) |  | 2.99 [-0.18, 6.15], .064 | 1.11 [-2.94, 5.16], .585 |
|  | CMT | 21, 21.05 (6.14) | 21, 21.48 (5.85) | 21, 19.76 (5.88) |  | 3.70 [0.53, 6.87], .023 | 2.60 [-1.30, 6.51], .187 |
| MADRS | SC | 18, 12.39 (6.23) | 18, 14.06 (7.85) | 18, 14.00 (7.47) | .452 |  |  |
|  | DMI | 18, 11.61 (6.82) | 18, 13.78 (7.55) | 18, 13.44 (7.24) |  | 0.58 [-2.86, 4.02], .737 | 0.31 [-3.40, 4.01], .869 |
|  | CMT | 21, 10.38 (6.72) | 21, 12.29 (8.46) | 21, 12.00 (8.80) |  | 0.72 [-2.72, 4.16], .676 | 0.09 [-3.47, 3.66], .959 |
| NPI-Q total | SC | 18, 14.61 (13.95) | 18, 16.22 (18.29) | 18, 15.28 (17.47) | .339 |  |  |
|  | DMI | 16, 13.81 (15.82) | 16, 21.00 (23.20) | 16, 16.06 (13.80) |  | 3.26 [-5.37, 11.89], .453 | 1.93 [-6.78, 10.65], .658 |
|  | CMT | 17, 10.59 (10.91) | 17, 11.24 (11.12) | 17, 12.47 (13.14) |  | -0.54 [-9.05, 7.98], .900 | 1.46 [-6.99, 9.91], .730 |
| QOL-AD | SC | 19, 31.53 (5.51) | 19, 30.66 (6.83) | 19, 30.21 (5.84) | .169 |  |  |
|  | DMI | 18, 30.86 (4.66) | 18, 29.31 (4.39) | 18, 29.06 (3.82) |  | -0.35 [-2.91, 2.20], .784 | -0.81 [-3.51, 1.90], .553 |
|  | CMT | 20, 31.48 (5.95) | 20, 29.82 (5.54) | 20, 30.15 (6.74) |  | -0.61 [-3.13, 1.92], .632 | -0.32 [-2.96, 2.31], .806 |
| EQ-5D-5L | SC | 19, 0.72 (0.14) | 19, 0.70 (0.14) | 19, 0.68 (0.14) | .090 |  |  |
|  | DMI | 18, 0.66 (0.19) | 18, 0.64 (0.21) | 18, 0.65 (0.14) |  | 0.02 [-0.07, 0.10], .716 | 0.03 [-0.06, 0.12], .442 |
|  | CMT | 21, 0.73 (0.17) | 21, 0.72 (0.19) | 21, 0.69 (0.23) |  | 0.01 [-0.08, 0.09], .891 | 0.01 [-0.08, 0.09], .894 |
| EQ VAS | SC | 19, 55.55 (23.17) | 19, 58.24 (21.98) | 19, 56.71 (22.64) | .418 |  |  |
|  | DMI | 17, 53.41 (18.04) | 17, 44.94 (19.90) | 17, 50.53 (19.52) |  | -10.24 [-22.53, 2.04], .101 | -3.61 [-15.82, 8.60], .556 |
|  | CMT | 19, 59.55 (15.69) | 19, 56.05 (19.69) | 19, 54.47 (19.27) |  | -3.59 [-15.73, 8.56], .557 | -5.80 [-17.66, 6.05], .331 |
| GHQ | SC | 12, 14.17 (9.63) | 12, 15.00 (8.94) | 12, 15.42 (7.90) | .790 |  |  |
|  | DMI | 14, 14.29 (8.07) | 14, 13.50 (7.53) | 14, 14.50 (8.66) |  | -1.78 [-4.64, 1.09], .218 | -1.09 [-3.91, 1.73], .438 |
|  | CMT | 15, 12.47 (7.80) | 15, 13.93 (8.88) | 15, 13.33 (7.63) |  | -0.28 [-3.14, 2.59], .847 | -0.44 [-3.22, 2.33], .749 |
| ZBI | SC | 12, 13.42 (8.58) | 12, 12.25 (7.92) | 12, 12.08 (8.01) | .362 |  |  |
|  | DMI | 14, 15.07 (12.05) | 14, 13.50 (10.23) | 14, 13.64 (9.90) |  | -0.42 [-3.68, 2.84], .797 | -0.12 [-3.52, 3.28], .943 |
|  | CMT | 15, 10.80 (10.42) | 15, 10.07 (10.22) | 15, 7.93 (6.94) |  | 0.27 [-2.99, 3.53], .869 | -1.56 [-4.91, 1.79], .352 |

NOTE. AM = autobiographical memory. B-ART = Brief Autobiographical Recollection Test. CARE = persons living with dementia, living in care homes. CERAD = Consortium to Establish a Registry for Alzheimer’s Disease. CMT = conventional music therapy. DMI = digital music intervention. GHQ = General Health Questionnaire. HOME = persons living with dementia, living at home. IFS = INECO Frontal Screening. LM = logical memory. MADRS = Montgomery-Åsberg Depression Rating Scale. MoCA = Montreal Cognitive Assessment. NPI-Q = Neuropsychiatric Inventory Questionnaire. PROMIS = Patient-Reported Outcomes Measurement Information System. SC = standard care. WL = word list. WMS-IV = Wechsler Memory Scale, Fourth Edition.

**TABLE S5.** Approximate effect sizes for primary and sensitivity analyses.

|  |  | Primary analysis  Cohen's *d* [95% CI] | | Sensitivity analysis  Cohen's *d* [95% CI] | |
| --- | --- | --- | --- | --- | --- |
| Variable | Group | T1-T2 | T1-T3 | T1-T2 | T1-T3 |
| All | | | | | |
| MoCA total | DMI | 0.01 [-0.14, 0.17] | -0.05 [-0.22, 0.12] | 0.05 [-0.11, 0.22] | -0.06 [-0.24, 0.13] |
|  | CMT | 0.06 [-0.10, 0.21] | -0.07 [-0.24, 0.11] | 0.05 [-0.11, 0.21] | -0.06 [-0.25, 0.12] |
| CERAD  Chandler total | DMI | 0.03 [-0.20, 0.26] | -0.05 [-0.28, 0.17] | 0.03 [-0.20, 0.26] | -0.03 [-0.25, 0.20] |
|  | CMT | 0.05 [-0.18, 0.28] | -0.00 [-0.24, 0.24] | 0.08 [-0.15, 0.32] | 0.03 [-0.20, 0.27] |
| Phonemic fluency | DMI | -0.02 [-0.26, 0.23] | -0.02 [-0.28, 0.23] | -0.03 [-0.29, 0.22] | -0.03 [-0.30, 0.24] |
|  | CMT | 0.04 [-0.19, 0.28] | 0.15 [-0.10, 0.41] | 0.06 [-0.19, 0.31] | 0.12 [-0.15, 0.39] |
| Categorical fluency | DMI | -0.05 [-0.32, 0.21] | 0.07 [-0.21, 0.36] | -0.08 [-0.35, 0.20] | 0.06 [-0.22, 0.35] |
|  | CMT | -0.06 [-0.32, 0.20] | -0.05 [-0.33, 0.24] | 0.01 [-0.26, 0.28] | 0.02 [-0.27, 0.30] |
| CERAD naming | DMI | 0.07 [-0.10, 0.24] | 0.02 [-0.19, 0.23] | 0.06 [-0.11, 0.23] | 0.02 [-0.20, 0.25] |
|  | CMT | **0.18 [0.02, 0.35]** | 0.01 [-0.20, 0.23] | **0.20 [0.03, 0.37]** | 0.02 [-0.21, 0.25] |
| CERAD copying | DMI | -0.04 [-0.34, 0.26] | -0.19 [-0.53, 0.15] | -0.05 [-0.35, 0.26] | -0.13 [-0.47, 0.21] |
|  | CMT | -0.07 [-0.37, 0.22] | 0.11 [-0.24, 0.46] | -0.05 [-0.35, 0.26] | 0.18 [-0.18, 0.53] |
| CERAD WL  immediate recall | DMI | 0.17 [-0.26, 0.60] | 0.01 [-0.44, 0.46] | 0.20 [-0.22, 0.63] | 0.06 [-0.40, 0.51] |
|  | CMT | -0.02 [-0.45, 0.41] | 0.04 [-0.42, 0.51] | 0.08 [-0.35, 0.51] | 0.10 [-0.39, 0.58] |
| CERAD WL  learning | DMI | -0.03 [-0.58, 0.51] | -0.21 [-0.79, 0.37] | -0.04 [-0.59, 0.50] | -0.17 [-0.76, 0.42] |
|  | CMT | 0.11 [-0.44, 0.65] | -0.02 [-0.62, 0.58] | -0.03 [-0.58, 0.52] | -0.08 [-0.70, 0.54] |
| CERAD WL  delayed recall | DMI | -0.04 [-0.44, 0.36] | 0.06 [-0.29, 0.41] | -0.01 [-0.41, 0.39] | 0.10 [-0.25, 0.45] |
|  | CMT | -0.05 [-0.45, 0.34] | -0.04 [-0.40, 0.33] | -0.03 [-0.43, 0.38] | -0.06 [-0.43, 0.31] |
| CERAD WL  recognition | DMI | 0.04 [-0.35, 0.42] | 0.23 [-0.22, 0.68] | 0.08 [-0.31, 0.48] | 0.28 [-0.17, 0.74] |
|  | CMT | 0.25 [-0.14, 0.63] | -0.01 [-0.48, 0.45] | 0.24 [-0.17, 0.64] | -0.01 [-0.49, 0.48] |
| WMS-IV LM  immediate recall | DMI | -0.10 [-0.56, 0.37] | -0.09 [-0.51, 0.34] | -0.02 [-0.50, 0.46] | -0.06 [-0.51, 0.39] |
|  | CMT | -0.34 [-0.81, 0.13] | -0.20 [-0.64, 0.23] | -0.26 [-0.75, 0.22] | -0.20 [-0.67, 0.26] |
| WMS-IV LM  learning | DMI | 0.14 [-0.47, 0.76] | 0.45 [-0.15, 1.05] | 0.10 [-0.53, 0.73] | 0.45 [-0.17, 1.08] |
|  | CMT | -0.05 [-0.67, 0.56] | **0.66 [0.06, 1.27]** | -0.08 [-0.72, 0.55] | 0.62 [-0.02, 1.27] |
| WMS-IV LM  delayed recall | DMI | -0.12 [-0.48, 0.24] | -0.09 [-0.46, 0.28] | -0.10 [-0.45, 0.26] | -0.06 [-0.43, 0.31] |
|  | CMT | -0.09 [-0.45, 0.26] | -0.23 [-0.61, 0.14] | -0.02 [-0.38, 0.34] | -0.16 [-0.53, 0.22] |
| IFS executive function | DMI | 0.10 [-0.21, 0.42] | 0.11 [-0.24, 0.46] | 0.08 [-0.23, 0.40] | 0.11 [-0.24, 0.45] |
|  | CMT | 0.12 [-0.20, 0.43] | 0.18 [-0.17, 0.54] | 0.15 [-0.17, 0.47] | 0.31 [-0.05, 0.66] |
| IFS working memory | DMI | -0.07 [-0.35, 0.22] | -0.05 [-0.32, 0.22] | -0.08 [-0.37, 0.22] | -0.02 [-0.30, 0.26] |
|  | CMT | 0.12 [-0.17, 0.40] | -0.01 [-0.28, 0.27] | 0.12 [-0.18, 0.42] | 0.01 [-0.27, 0.30] |
| IFS total | DMI | 0.03 [-0.19, 0.25] | 0.06 [-0.20, 0.32] | 0.02 [-0.21, 0.24] | 0.06 [-0.21, 0.33] |
|  | CMT | 0.13 [-0.08, 0.35] | 0.13 [-0.13, 0.40] | 0.15 [-0.07, 0.38] | 0.21 [-0.07, 0.48] |
| Autobiographical  interview | DMI | 0.03 [-0.24, 0.31] | 0.01 [-0.29, 0.31] | 0.02 [-0.30, 0.33] | -0.03 [-0.34, 0.29] |
|  | CMT | 0.25 [-0.03, 0.52] | 0.06 [-0.25, 0.36] | 0.29 [-0.03, 0.60] | 0.14 [-0.18, 0.46] |
| B-ART | DMI | 0.18 [-0.14, 0.50] | 0.11 [-0.24, 0.45] | 0.15 [-0.16, 0.46] | 0.18 [-0.19, 0.55] |
|  | CMT | 0.09 [-0.22, 0.41] | -0.12 [-0.46, 0.22] | 0.25 [-0.07, 0.56] | -0.03 [-0.41, 0.34] |
| Spontaneous  AM | DMI | 0.06 [-0.19, 0.30] | 0.14 [-0.18, 0.46] | 0.08 [-0.17, 0.34] | 0.20 [-0.17, 0.57] |
|  | CMT | 0.16 [-0.08, 0.40] | 0.24 [-0.07, 0.56] | 0.25 [-0.00, 0.51] | 0.36 [-0.01, 0.72] |
| Prompted  AM | DMI | **0.44 [0.12, 0.77]** | 0.12 [-0.23, 0.46] | **0.43 [0.09, 0.77]** | 0.16 [-0.22, 0.55] |
|  | CMT | **0.35 [0.04, 0.67]** | 0.05 [-0.29, 0.39] | **0.44 [0.09, 0.78]** | 0.11 [-0.27, 0.50] |
| MADRS | DMI | 0.12 [-0.17, 0.42] | 0.07 [-0.23, 0.38] | 0.07 [-0.24, 0.38] | 0.03 [-0.28, 0.34] |
|  | CMT | -0.07 [-0.37, 0.22] | -0.05 [-0.35, 0.24] | -0.08 [-0.39, 0.23] | -0.09 [-0.39, 0.21] |
| PROMIS depression | DMI | -0.15 [-0.59, 0.30] | 0.11 [-0.39, 0.60] | -0.14 [-0.60, 0.32] | 0.13 [-0.37, 0.64] |
|  | CMT | -0.17 [-0.62, 0.28] | 0.11 [-0.40, 0.61] | -0.12 [-0.59, 0.34] | 0.18 [-0.34, 0.70] |
| PROMIS anxiety | DMI | -0.01 [-0.46, 0.44] | 0.07 [-0.40, 0.53] | 0.09 [-0.37, 0.54] | 0.16 [-0.31, 0.63] |
|  | CMT | -0.16 [-0.61, 0.30] | -0.31 [-0.79, 0.17] | -0.13 [-0.60, 0.33] | -0.29 [-0.78, 0.20] |
| NPI-Q total | DMI | -0.03 [-0.34, 0.29] | 0.00 [-0.32, 0.33] | -0.07 [-0.42, 0.28] | -0.06 [-0.43, 0.31] |
|  | CMT | -0.14 [-0.45, 0.17] | 0.08 [-0.25, 0.40] | -0.12 [-0.47, 0.23] | 0.09 [-0.27, 0.46] |
| QOL-AD | DMI | -0.03 [-0.30, 0.23] | -0.00 [-0.27, 0.26] | -0.02 [-0.29, 0.24] | -0.01 [-0.27, 0.25] |
|  | CMT | 0.08 [-0.18, 0.34] | 0.08 [-0.18, 0.34] | 0.13 [-0.14, 0.39] | 0.15 [-0.11, 0.41] |
| EQ-5D-5L | DMI | 0.13 [-0.14, 0.41] | 0.11 [-0.18, 0.40] | 0.13 [-0.14, 0.40] | 0.17 [-0.10, 0.44] |
|  | CMT | 0.05 [-0.22, 0.32] | 0.00 [-0.28, 0.29] | 0.08 [-0.19, 0.35] | 0.06 [-0.21, 0.33] |
| EQ VAS | DMI | -0.25 [-0.61, 0.11] | 0.07 [-0.25, 0.39] | -0.26 [-0.63, 0.11] | 0.03 [-0.31, 0.37] |
|  | CMT | -0.14 [-0.50, 0.21] | -0.06 [-0.37, 0.25] | -0.16 [-0.53, 0.22] | -0.04 [-0.38, 0.29] |
| GHQ | DMI | 0.03 [-0.22, 0.29] | 0.01 [-0.27, 0.29] | 0.00 [-0.26, 0.26] | -0.06 [-0.36, 0.23] |
|  | CMT | 0.07 [-0.18, 0.33] | -0.02 [-0.31, 0.26] | 0.11 [-0.15, 0.37] | -0.01 [-0.31, 0.29] |
| ZBI | DMI | 0.06 [-0.19, 0.31] | -0.03 [-0.28, 0.21] | 0.04 [-0.22, 0.29] | -0.07 [-0.34, 0.20] |
|  | CMT | 0.05 [-0.20, 0.30] | -0.12 [-0.37, 0.13] | 0.08 [-0.18, 0.33] | -0.13 [-0.40, 0.14] |
| HOME | | | | | |
| MoCA total | DMI | 0.16 [-0.11, 0.44] | -0.04 [-0.32, 0.24] | 0.17 [-0.10, 0.45] | -0.03 [-0.31, 0.26] |
|  | CMT | 0.17 [-0.11, 0.45] | -0.10 [-0.38, 0.19] | 0.15 [-0.12, 0.43] | -0.10 [-0.40, 0.19] |
| CERAD  Chandler total | DMI | -0.04 [-0.33, 0.26] | -0.03 [-0.32, 0.26] | -0.03 [-0.33, 0.27] | -0.02 [-0.30, 0.27] |
|  | CMT | -0.10 [-0.40, 0.20] | -0.04 [-0.34, 0.27] | -0.10 [-0.40, 0.21] | 0.00 [-0.30, 0.30] |
| Phonemic fluency | DMI | 0.05 [-0.37, 0.47] | 0.01 [-0.40, 0.42] | 0.08 [-0.35, 0.51] | 0.06 [-0.36, 0.49] |
|  | CMT | 0.14 [-0.28, 0.56] | 0.21 [-0.21, 0.62] | 0.12 [-0.31, 0.55] | 0.21 [-0.22, 0.64] |
| Categorical fluency | DMI | -0.09 [-0.52, 0.33] | 0.09 [-0.35, 0.52] | -0.07 [-0.52, 0.37] | 0.15 [-0.27, 0.57] |
|  | CMT | -0.08 [-0.50, 0.35] | -0.06 [-0.49, 0.38] | -0.04 [-0.48, 0.41] | 0.04 [-0.38, 0.46] |
| CERAD naming | DMI | 0.03 [-0.17, 0.22] | 0.05 [-0.21, 0.30] | 0.04 [-0.16, 0.24] | 0.04 [-0.23, 0.31] |
|  | CMT | 0.13 [-0.06, 0.33] | 0.09 [-0.17, 0.34] | 0.14 [-0.06, 0.34] | 0.08 [-0.19, 0.36] |
| CERAD copying | DMI | 0.11 [-0.27, 0.49] | -0.08 [-0.52, 0.36] | 0.09 [-0.29, 0.47] | -0.08 [-0.55, 0.38] |
|  | CMT | 0.01 [-0.37, 0.39] | 0.11 [-0.34, 0.56] | 0.04 [-0.34, 0.42] | 0.11 [-0.37, 0.60] |
| CERAD WL  immediate recall | DMI | -0.14 [-0.65, 0.36] | -0.09 [-0.58, 0.41] | -0.14 [-0.65, 0.38] | -0.09 [-0.59, 0.41] |
|  | CMT | -0.37 [-0.87, 0.14] | 0.01 [-0.51, 0.52] | -0.35 [-0.87, 0.17] | 0.04 [-0.49, 0.56] |
| CERAD WL  learning | DMI | 0.14 [-0.47, 0.75] | -0.01 [-0.70, 0.69] | 0.14 [-0.47, 0.75] | -0.03 [-0.72, 0.65] |
|  | CMT | 0.15 [-0.47, 0.77] | -0.03 [-0.74, 0.68] | 0.08 [-0.54, 0.69] | -0.13 [-0.85, 0.58] |
| CERAD WL  delayed recall | DMI | -0.09 [-0.57, 0.38] | 0.07 [-0.35, 0.49] | -0.09 [-0.57, 0.39] | 0.08 [-0.34, 0.50] |
|  | CMT | -0.15 [-0.63, 0.33] | -0.08 [-0.52, 0.35] | -0.18 [-0.67, 0.30] | -0.13 [-0.57, 0.31] |
| CERAD WL  recognition | DMI | -0.26 [-0.72, 0.20] | -0.03 [-0.56, 0.50] | -0.22 [-0.68, 0.25] | 0.04 [-0.50, 0.57] |
|  | CMT | 0.07 [-0.40, 0.54] | -0.29 [-0.84, 0.26] | 0.08 [-0.39, 0.55] | -0.28 [-0.84, 0.28] |
| WMS-IV LM  immediate recall | DMI | -0.17 [-0.69, 0.35] | -0.16 [-0.70, 0.38] | -0.18 [-0.70, 0.35] | -0.15 [-0.70, 0.39] |
|  | CMT | **-0.53 [-1.05, -0.00]** | -0.32 [-0.88, 0.23] | **-0.54 [-1.07, -0.01]** | -0.34 [-0.90, 0.23] |
| WMS-IV LM  learning | DMI | 0.18 [-0.56, 0.91] | 0.38 [-0.37, 1.14] | 0.23 [-0.52, 0.98] | 0.35 [-0.43, 1.12] |
|  | CMT | -0.15 [-0.89, 0.59] | 0.52 [-0.25, 1.29] | -0.04 [-0.79, 0.72] | 0.51 [-0.29, 1.31] |
| WMS-IV LM  delayed recall | DMI | -0.13 [-0.57, 0.32] | -0.09 [-0.56, 0.38] | -0.12 [-0.56, 0.33] | -0.07 [-0.54, 0.39] |
|  | CMT | 0.02 [-0.43, 0.47] | -0.25 [-0.73, 0.23] | 0.10 [-0.35, 0.54] | -0.17 [-0.65, 0.31] |
| IFS executive function | DMI | 0.30 [-0.06, 0.65] | 0.11 [-0.32, 0.54] | 0.26 [-0.09, 0.61] | 0.11 [-0.31, 0.53] |
|  | CMT | 0.31 [-0.04, 0.67] | 0.28 [-0.16, 0.72] | **0.38 [0.02, 0.73]** | 0.43 [-0.00, 0.86] |
| IFS working memory | DMI | 0.09 [-0.23, 0.41] | -0.12 [-0.47, 0.23] | 0.08 [-0.24, 0.41] | -0.10 [-0.45, 0.26] |
|  | CMT | 0.16 [-0.17, 0.49] | -0.11 [-0.46, 0.25] | 0.13 [-0.20, 0.45] | -0.11 [-0.47, 0.26] |
| IFS total | DMI | 0.24 [-0.03, 0.50] | 0.03 [-0.31, 0.37] | 0.21 [-0.06, 0.47] | 0.03 [-0.31, 0.37] |
|  | CMT | **0.28 [0.01, 0.55]** | 0.16 [-0.20, 0.51] | **0.30 [0.03, 0.57]** | 0.24 [-0.11, 0.59] |
| Autobiographical  interview | DMI | 0.01 [-0.40, 0.43] | 0.11 [-0.34, 0.57] | 0.01 [-0.41, 0.43] | 0.11 [-0.35, 0.56] |
|  | CMT | 0.31 [-0.11, 0.73] | 0.22 [-0.24, 0.69] | 0.28 [-0.15, 0.70] | 0.23 [-0.24, 0.70] |
| B-ART | DMI | 0.28 [-0.22, 0.77] | 0.09 [-0.48, 0.66] | 0.25 [-0.25, 0.75] | 0.07 [-0.51, 0.65] |
|  | CMT | 0.27 [-0.23, 0.77] | -0.09 [-0.67, 0.48] | 0.26 [-0.25, 0.77] | -0.10 [-0.68, 0.48] |
| Spontaneous  AM | DMI | 0.33 [-0.11, 0.77] | 0.26 [-0.21, 0.72] | 0.32 [-0.11, 0.76] | 0.27 [-0.20, 0.73] |
|  | CMT | 0.23 [-0.21, 0.67] | 0.07 [-0.39, 0.54] | 0.23 [-0.21, 0.68] | 0.08 [-0.38, 0.55] |
| Prompted  AM | DMI | 0.44 [-0.12, 0.99] | 0.10 [-0.40, 0.61] | 0.43 [-0.12, 0.99] | 0.16 [-0.34, 0.66] |
|  | CMT | 0.32 [-0.23, 0.88] | -0.20 [-0.71, 0.31] | 0.33 [-0.23, 0.89] | -0.23 [-0.74, 0.27] |
| MADRS | DMI | 0.06 [-0.34, 0.45] | 0.12 [-0.25, 0.50] | 0.07 [-0.32, 0.46] | 0.04 [-0.32, 0.40] |
|  | CMT | -0.25 [-0.65, 0.15] | -0.16 [-0.54, 0.21] | -0.24 [-0.63, 0.16] | -0.19 [-0.55, 0.18] |
| PROMIS depression | DMI | -0.27 [-0.80, 0.27] | -0.04 [-0.56, 0.48] | -0.24 [-0.78, 0.31] | 0.00 [-0.52, 0.53] |
|  | CMT | -0.27 [-0.80, 0.27] | 0.06 [-0.46, 0.58] | -0.24 [-0.79, 0.31] | 0.09 [-0.44, 0.62] |
| PROMIS anxiety | DMI | -0.19 [-0.67, 0.29] | -0.24 [-0.73, 0.26] | -0.17 [-0.66, 0.33] | -0.20 [-0.71, 0.31] |
|  | CMT | -0.34 [-0.82, 0.14] | -0.48 [-0.98, 0.02] | -0.33 [-0.83, 0.16] | **-0.54 [-1.05, -0.02]** |
| NPI-Q total | DMI | -0.24 [-0.67, 0.18] | -0.06 [-0.50, 0.39] | -0.28 [-0.72, 0.15] | -0.18 [-0.64, 0.28] |
|  | CMT | -0.16 [-0.58, 0.26] | 0.11 [-0.34, 0.57] | -0.19 [-0.63, 0.25] | 0.09 [-0.38, 0.56] |
| QOL-AD | DMI | 0.00 [-0.41, 0.42] | 0.08 [-0.30, 0.46] | 0.00 [-0.41, 0.41] | 0.09 [-0.29, 0.46] |
|  | CMT | 0.40 [-0.02, 0.82] | 0.33 [-0.06, 0.71] | 0.38 [-0.04, 0.79] | 0.35 [-0.04, 0.73] |
| EQ-5D-5L | DMI | 0.30 [-0.15, 0.76] | 0.21 [-0.27, 0.69] | 0.28 [-0.17, 0.73] | 0.23 [-0.26, 0.72] |
|  | CMT | 0.22 [-0.24, 0.68] | 0.15 [-0.33, 0.64] | 0.20 [-0.26, 0.66] | 0.13 [-0.36, 0.62] |
| EQ VAS | DMI | 0.01 [-0.54, 0.57] | 0.32 [-0.12, 0.77] | -0.02 [-0.58, 0.54] | 0.26 [-0.18, 0.71] |
|  | CMT | -0.06 [-0.62, 0.49] | 0.22 [-0.23, 0.67] | -0.16 [-0.74, 0.41] | 0.23 [-0.22, 0.68] |
| GHQ | DMI | 0.19 [-0.24, 0.62] | 0.09 [-0.39, 0.57] | 0.21 [-0.21, 0.62] | 0.00 [-0.46, 0.47] |
|  | CMT | 0.18 [-0.24, 0.61] | 0.01 [-0.48, 0.50] | 0.25 [-0.17, 0.67] | 0.02 [-0.46, 0.50] |
| ZBI | DMI | 0.08 [-0.31, 0.47] | -0.12 [-0.50, 0.25] | 0.10 [-0.31, 0.50] | -0.12 [-0.51, 0.28] |
|  | CMT | 0.09 [-0.30, 0.48] | -0.10 [-0.47, 0.27] | 0.12 [-0.29, 0.52] | -0.11 [-0.51, 0.28] |
| CARE | | | | | |
| MoCA total | DMI | -0.18 [-0.42, 0.05] | -0.12 [-0.41, 0.18] | -0.10 [-0.35, 0.16] | -0.18 [-0.52, 0.17] |
|  | CMT | -0.06 [-0.29, 0.16] | -0.07 [-0.37, 0.22] | -0.05 [-0.30, 0.19] | -0.04 [-0.37, 0.30] |
| Phonemic fluency | DMI | -0.14 [-0.54, 0.25] | -0.10 [-0.55, 0.34] | -0.19 [-0.62, 0.24] | -0.19 [-0.69, 0.31] |
|  | CMT | -0.09 [-0.47, 0.30] | 0.12 [-0.32, 0.57] | 0.02 [-0.39, 0.43] | 0.05 [-0.44, 0.54] |
| Categorical fluency | DMI | -0.06 [-0.49, 0.37] | 0.03 [-0.49, 0.56] | -0.17 [-0.59, 0.26] | -0.17 [-0.75, 0.41] |
|  | CMT | -0.08 [-0.49, 0.33] | -0.06 [-0.59, 0.46] | 0.09 [-0.31, 0.50] | -0.05 [-0.61, 0.51] |
| Autobiographical  interview | DMI | 0.08 [-0.46, 0.62] | -0.17 [-0.72, 0.39] | 0.05 [-0.63, 0.74] | -0.33 [-0.94, 0.29] |
|  | CMT | 0.27 [-0.25, 0.78] | -0.23 [-0.79, 0.34] | 0.40 [-0.26, 1.07] | 0.03 [-0.60, 0.65] |
| B-ART | DMI | 0.13 [-0.32, 0.57] | 0.15 [-0.32, 0.62] | 0.06 [-0.37, 0.49] | 0.26 [-0.27, 0.79] |
|  | CMT | -0.01 [-0.45, 0.43] | -0.13 [-0.59, 0.34] | 0.24 [-0.18, 0.67] | -0.00 [-0.53, 0.52] |
| Spontaneous  AM | DMI | -0.10 [-0.45, 0.24] | 0.09 [-0.42, 0.60] | -0.10 [-0.47, 0.26] | 0.18 [-0.46, 0.82] |
|  | CMT | 0.15 [-0.18, 0.49] | 0.37 [-0.13, 0.87] | 0.31 [-0.05, 0.67] | **0.62 [0.01, 1.24]** |
| Prompted  AM | DMI | **0.52 [0.06, 0.98]** | 0.15 [-0.41, 0.70] | 0.48 [-0.03, 0.99] | 0.18 [-0.49, 0.85] |
|  | CMT | 0.42 [-0.03, 0.87] | 0.23 [-0.30, 0.76] | **0.59 [0.09, 1.10]** | 0.43 [-0.22, 1.08] |
| MADRS | DMI | 0.19 [-0.24, 0.63] | 0.03 [-0.45, 0.50] | 0.08 [-0.40, 0.56] | 0.04 [-0.47, 0.55] |
|  | CMT | 0.07 [-0.36, 0.50] | 0.02 [-0.44, 0.48] | 0.10 [-0.38, 0.58] | 0.01 [-0.48, 0.51] |
| NPI-Q total | DMI | 0.19 [-0.28, 0.66] | 0.09 [-0.40, 0.58] | 0.21 [-0.35, 0.78] | 0.14 [-0.48, 0.75] |
|  | CMT | -0.12 [-0.58, 0.33] | 0.04 [-0.42, 0.51] | -0.04 [-0.60, 0.53] | 0.10 [-0.49, 0.70] |
| QOL-AD | DMI | -0.11 [-0.58, 0.36] | -0.12 [-0.64, 0.39] | -0.07 [-0.54, 0.41] | -0.15 [-0.64, 0.35] |
|  | CMT | -0.22 [-0.68, 0.24] | -0.19 [-0.69, 0.31] | -0.11 [-0.58, 0.36] | -0.06 [-0.54, 0.42] |
| EQ-5D-5L | DMI | 0.06 [-0.43, 0.55] | 0.08 [-0.48, 0.65] | 0.09 [-0.40, 0.57] | 0.20 [-0.32, 0.72] |
|  | CMT | -0.06 [-0.54, 0.42] | -0.09 [-0.63, 0.46] | 0.03 [-0.45, 0.52] | 0.03 [-0.47, 0.53] |
| EQ VAS | DMI | -0.49 [-1.06, 0.07] | -0.16 [-0.72, 0.40] | -0.50 [-1.10, 0.10] | -0.18 [-0.80, 0.43] |
|  | CMT | -0.21 [-0.77, 0.34] | -0.28 [-0.82, 0.26] | -0.17 [-0.77, 0.42] | -0.29 [-0.89, 0.31] |
| GHQ | DMI | -0.13 [-0.43, 0.17] | -0.09 [-0.39, 0.22] | -0.20 [-0.52, 0.12] | -0.13 [-0.47, 0.21] |
|  | CMT | -0.04 [-0.34, 0.26] | -0.06 [-0.36, 0.24] | -0.03 [-0.35, 0.29] | -0.05 [-0.38, 0.28] |
| ZBI | DMI | 0.04 [-0.28, 0.37] | 0.11 [-0.21, 0.42] | -0.04 [-0.34, 0.26] | -0.01 [-0.37, 0.35] |
|  | CMT | 0.02 [-0.31, 0.34] | -0.13 [-0.44, 0.18] | 0.02 [-0.27, 0.32] | -0.17 [-0.52, 0.19] |

NOTE. AM = autobiographical memory. B-ART = Brief Autobiographical Recollection Test. CARE = persons living with dementia, living in care homes. CERAD = Consortium to Establish a Registry for Alzheimer’s Disease. CMT = conventional music therapy. DMI = digital music intervention. GHQ = General Health Questionnaire. HOME = persons living with dementia, living at home. IFS = INECO Frontal Screening. LM = logical memory. MADRS = Montgomery-Åsberg Depression Rating Scale. MoCA = Montreal Cognitive Assessment. NPI-Q = Neuropsychiatric Inventory Questionnaire. PROMIS = Patient-Reported Outcomes Measurement Information System. SC = standard care. WL = word list. WMS-IV = Wechsler Memory Scale, Fourth Edition. Reported Cohen’s *d* values represent the standardised difference relative to standard care, calculated by dividing interaction estimates outlined in Table S4 and S5 with each model’s pooled random and residual variance. These values serve as approximations of standardised differences rather than exact effect sizes. Values for statistically significant Time x Group interactions are marked in bold.

**TABLE S6.** Follow-up LMM analyses comparing DMI and CMT on statistically significant main analysis effects.

| Variable | Analysis | Subgroup | Timepoint | DMI vs. CMT Interaction β [95% CI], *P* |
| --- | --- | --- | --- | --- |
| CERAD naming | Primary | All | T1-T2 | -0.41 [-0.98, 0.15], 0.151 |
|  | Sensitivity | All | T1-T2 | 0.53 [-0.05, 1.11], 0.070 |
| WMS-IV LM immediate recall | Primary | HOME | T1-T2 | 1.01 [-0.37, 2.39], 0.147 |
|  | Sensitivity | HOME | T1-T2 | -1.05 [-2.48, 0.39], 0.148 |
| WMS-IV LM learning | Primary | All | T1-T3 | -0.50 [-1.83, 0.83], 0.456 |
| IFS executive function | Sensitivity | HOME | T1-T2 | 0.44 [-1.02, 1.90], 0.544 |
| IFS total | Primary | HOME | T1-T2 | -0.26 [-1.94, 1.41], 0.752 |
|  | Sensitivity | HOME | T1-T2 | 0.56 [-1.13, 2.25], 0.506 |
| Spontaneous AM | Sensitivity | CARE | T1-T3 | 2.48 [-1.27, 6.23], 0.189 |
| Prompted AM | Primary | All | T1-T2 | 0.56 [-1.18, 2.31], 0.523 |
|  | Primary | CARE | T1-T2 | 0.61 [-1.96, 3.19], 0.635 |
|  | Sensitivity | All | T1-T2 | 0.06 [-1.81, 1.93], 0.947 |
|  | Sensitivity | CARE | T1-T2 | 0.71 [-2.18, 3.61], 0.620 |
| PROMIS Anxiety | Sensitivity | HOME | T1-T3 | -2.96 [-7.36, 1.44], 0.182 |

NOTE. AM = autobiographical memory. CARE = persons living with dementia, living in care homes. CERAD = Consortium to Establish a Registry for Alzheimer’s Disease. CMT = conventional music therapy. DMI = digital music intervention. HOME = persons living with dementia, living at home. IFS = INECO Frontal Screening. LM = logical memory. PROMIS = Patient-Reported Outcomes Measurement Information System. WMS-IV = Wechsler Memory Scale, Fourth Edition.

**Description of qualitative feedback from participants and caregivers.**

Qualitative feedback from participants was categorised into positive and negative feedback. The positive feedback from participants in the DMI group most frequently addressed the music used in the intervention, community, and cognitive/emotional benefits of the intervention, whereas for CMT, positive feedback most frequently addressed community, singing, and emotional benefits. Concerning negative feedback, DMI and CMT participants most frequently addressed shortness of the intervention. DMI participants also addressed music not being to taste, lack of connection with other participants, and technical problems (in the HOME blocks).

Caregiver feedback was categorised into positive and negative feedback separately for comments concerning benefits for participants and caregivers. The positive feedback concerning benefits for participants in the DMI group most frequently addressed cognitive and emotional benefits, change of pace in daily life, and increased interest in music, whereas for CMT, feedback most frequently addressed emotional, social, and cognitive benefits. Concerning benefits for caregivers, feedback for both DMI and CMT most frequently addressed enhanced interaction with participants and emotional benefits. Care home staff mentioned learning new ways to form deeper connections with persons living with dementia. Negative feedback for DMI mirrored feedback from participants. CMT received only three negative comments concerning the lack of participant benefits and neither intervention received negative comments concerning caregiver benefits. See below examples of qualitative feedback.

| Respondent | Group | Category | Examples of feedback |
| --- | --- | --- | --- |
| Participants | DMI | Positive | “Old, beautiful songs that brought back good memories.”  “Every day spent here was the best day. I always left feeling happy.” |
|  | DMI | Negative | “Old music was not to my taste.”  “Technical problems, the sound quality was not the best.” |
|  | CMT | Positive | “Warm atmosphere, time and space for everyone. Everyone was polite.” “I discovered a singer in myself. I can join communal singing and maintain this new skill.” |
|  | CMT | Negative | “Sometimes it felt like the hour went by too fast.” “Long journey and the session time was short.” |
| Caregivers | DMI | Positive | “He/she always looked forward to the sessions and considered them important, seemed more determined than before.” “We got a new, interesting topic of conversation. It was great to see how enthusiastic he/she was about the study.” |
|  | DMI | Negative | “Remote group activity is challenging with severe dementia.”  “The chosen music seemed too old, worn-out and simple.” |
|  | CMT | Positive | “Mood and sociability have increased, with it interest in other participants. He/she has enjoyed participation and singing.” “Overall, I feel that this has helped me cope better.” |
|  | CMT | Negative | “Benefits would be longer lasting if the intervention was longer.”  “I think he/she felt that he/she didn’t belong to the group.” |

NOTE. DMI = digital music intervention. CMT = conventional music therapy.

**TABLE S7.** All adverse events in group sessions as reported by research assistants.

| Block | Intervention | Session no. | Description |
| --- | --- | --- | --- |
| Block 1 | DMI | 1 | Participant A was anxious upon arrival to session and continued to express anxiety loudly, disrupting the group session. RA expressed doubt of participant meeting inclusion criteria due to significant difficulties with hearing, seeing, and ability to participate in group activities. |
| Block 1 | CMT | 1 | Participant B appeared significantly restless and unable to focus on group activities, instead moving items around or constantly making sound. RA expressed doubt of participant meeting inclusion criteria due to significant difficulty with participating in group activities. |
| Block 1 | DMI | 2 | Participant A expressed anxiety with similar disruptive effects as previously, indicating that inclusion criteria for study were not met. Participation was discontinued by mutual decision by care home staff and research team. |
| Block 1 | CMT | 2 | Participant B appeared significantly restless, unable to focus on group activities, and unwilling to participate in group activities. Participation was discontinued by mutual agreement of care home staff and research team. |
| Block 2 | DMI | 11 | Participant C suddenly expressed feeling dizzy and unwell and hence was escorted back to group home with help of care home staff. |
| Block 2 | DMI | 12 | Participant C had an episode resembling a seizure. RA immediately seeked help for the situation from care home staff. Care home staff indicated participant C had experienced these episodes before. The episode lasted for a few minutes, after which participant C appeared to be feeling well again. Care home staff escorted participant C to rest. One participant appeared restless after episode and hence left group session, but other participants did not appear disturbed. RA and DMI facilitator went to check on participant C after conclusion of group session and C was feeling well. |
| Block 2 | DMI | 17 | Participant C experienced another episode at the very end of group session. RA received help from care home staff swiftly. Participant C recovered quickly and was escorted to rest by care home staff, who later reported participant C to be feeling well. |
| Block 3 | CMT | 19 | Participant D suddenly expressed feeling unwell and an urgent need to use a restroom. Participant D was escorted back to group home by care home staff. |
| Block 4 | DMI | 7 | Participant E was asked to reminisce on youth with the help of music and photographs. The caregiver of participant E helped in answering questions, but participant E appeared frustrated and turned camera away from self. RA and the caregiver came to a mutual agreement to avoid triggering frustration by simplifying questions aimed at participant E in future group sessions. |

NOTE. DMI = digital music intervention. CMT = conventional music therapy. RA= research assistant.
